# Unveiling Molecular Dynamics of MeCp2, CDKL5 and BDNF in the Hippocampus of Individuals With Intractable Mesial Temporal Lobe Epilepsy

**DOI:** 10.1111/jcmm.70373

**Published:** 2025-01-31

**Authors:** NoorMohammad Meshkinkhood, Parastoo Barati Dowom, Farshid Noorbakhsh, Masoud Ghadipasha, Jaber Gharehdaghi, Christoph Kellinghaus, Erwin‐Joseph Speckmann, Maryam Khaleghi Ghadiri, Walter Stummer, Ali Gorji

**Affiliations:** ^1^ Shefa Neuroscience Research Center Khatam Alanbia Hospital Tehran Iran; ^2^ Legal Medicine Research Center Legal Medicine Organization Tehran Iran; ^3^ Department of Neurology Klinikum Osnabrück Osnabrück Germany; ^4^ Department of Neurosurgery University of Münster Münster Germany; ^5^ Epilepsy Research Center University of Münster Münster Germany; ^6^ Neuroscience Research Center Mashhad University of Medical Sciences Mashhad Iran

**Keywords:** brain, drug‐refractory, epilepsies, hyperexcitability, seizure

## Abstract

Mutations occurring in the MeCp2, CDKL5 and BDNF genes have been linked to epileptogenesis in various epilepsy syndromes. This study employed bioinformatics analysis of transcriptomic data to examine the interrelationship among these genes in both epileptic and healthy individuals. Moreover, we assessed the expression of MeCp2, CDKL5 and BDNF at both mRNA and protein levels in human hippocampal tissues obtained from 22 patients undergoing epilepsy surgery for mesial temporal lobe epilepsy (MTLE) as well as from 25 autopsied specimens. Bioinformatics findings suggest that MeCp2, CDKL5 and BDNF genes play a role in regulating genes associated with epilepsy and disruptions in these genes may contribute to epilepsy development. Furthermore, the study reveals significantly lower MeCp2 and CDKL5 protein levels in the epileptic hippocampus compared to controls. Positive correlations are observed between MeCp2 and CDKL5 mRNA expression in autopsied samples and between CDKL5 and BDNF mRNA expression in epileptic hippocampal tissues. Differences in mRNA expression correlation patterns of MeCp2 and CDKL5 with BDNF are found between epileptic and control hippocampal tissues. Moreover, a significant positive correlation between MeCp2 and CDKL5 protein expression is noted in control hippocampal tissues. Our data suggest that altered expression of MeCp2, CDKL5 and BDNF within the hippocampus may contribute to epileptogenic processes in MTLE, impacting seizure characteristics, surgical outcomes and responses to antiepileptic drugs. Alterations in the expression of MeCp2, CDKL5 and BDNF within the hippocampus might contribute to the epileptogenic processes in MTLE. These changes could be linked to distinct functional consequences in epilepsy.

## 1 Introduction

Epilepsy accounts for approximately 0.5% of the global disease burden, affecting about 50 million people worldwide [1]. Seizures in about 30% to 40% of individuals with epilepsy fail to respond to currently available anticonvulsants [2, 3]. Therapeutic approaches still face limitations due to the lack of identification of the precise mechanisms involved in epileptogenesis [4]. Improving our understanding of the molecular mechanisms governing seizure initiation and propagation could determine novel potential diagnostic and therapeutic targets. Among different pathological mechanisms, epigenetic alterations in gene expression affect neuronal network excitability and synaptic plasticity through DNA methylation and histone modifications and contribute to epileptogenesis [5, 6].

Methyl CpG binding protein 2 (MeCp2), an X‐linked epigenetic factor binding to the methylated DNA sites, modulates gene expression, modifies histones and chromatin architecture and regulates RNA splicing [7, 8, 9, 10]. MeCp2 is a DNA‐binding protein, which is implicated in transcriptional modulation [11] and plays a role in neurodevelopment, maturation and synaptogenesis in the central nervous system [12, 13]. MeCp2 protein dysregulation leads to developmental delay associated with a wide variety of neurological symptoms, including seizures [14]. Patients with Rett syndrome or MeCp2 duplication syndrome develop various neurological symptoms, such as absent speech, gait disturbance, cognitive impairments and epilepsy [15]. *MeCp2* mutations have been observed in more than 95% of patients with typical Rett syndrome and about 75% of individuals with atypical Rett syndrome [16]. Furthermore, mutations within the cyclin‐dependent kinase‐like 5 (*CDKL5*) gene result in various neurological disorders with epilepsy, like Rett syndrome and West syndrome [17]. *CDKL5* is a MeCp2 target gene in the brain [18] Previous studies have demonstrated that CDKL5 and MeCp2 possess some interactions during neural maturation and synaptogenesis [19]. Therefore, it is suggested that CDKL5 and MeCp2 might share the same molecular pathway in both physiological and pathological processes in CNS [20]. Furthermore, mutations in MeCp2 alter the expression of brain‐derived neurotrophic factor (BDNF), which plays a pivotal role in neuronal development, plasticity and survival. BDNF appears to be involved in the initiation and progression of the Rett syndrome phenotype in mice. *MeCp2* knockout mice have lower levels of BDNF, and conditional deletion of BDNF in *MeCp2* knockout mice expedites the onset of Rett syndrome‐like symptoms [21]. On the contrary, BDNF overexpression in the brain of *MeCp2* knockout mice leads to ameliorating epileptiform potentials [21, 22].

Alternations of MeCP2 expression are associated with changes in the hippocampal neural circuit excitability and synaptic plasticity that lead to the occurrence of epileptiform burst discharges and seizure‐like behaviours in animal studies [23, 24]. Furthermore, the changes in MeCp2 expression have been identified in the temporal neocortex tissues of patients with mesial temporal lobe epilepsy (MTLE) and the hippocampus of an animal model of MTLE [25]. The present study aimed to (i) analyse existing transcriptomic data and identify the differential expressed genes (DEGs) between epileptic and healthy individuals, and identify co‐expressed genes linked to MeCp2, CDKL5 and BDNF in the temporal lobe of healthy individuals, (ii) evaluate the expression values of MeCp2, CDKL5 and BDNF at both mRNA and protein levels in human epileptic hippocampal tissues; which were systematically compared to findings from autoptic specimens, and (iii) explore the potential correlation between the expression values of MeCp2, CDKL5 and BDNF in the epileptic hippocampus as well as their correlation with demographic and clinical characteristics of patients undergoing surgical interventions.

## 2 Materials and Methods

### 2.1 Differentially Expressed Genes (DEGs) Analysis

The RNA sequencing gene expression dataset with the accession number GSE139914 underwent analysis using the GEO2R online tool to detect DEGs between the “Epileptic” and “Control” groups. This dataset contains transcriptomic analysis outcomes from 56 samples, including post‐mortem epileptic, resected epileptic and post‐mortem healthy samples. Our analysis focused on batch 1 samples, consisting of 12 post‐mortem healthy samples as controls and 12 pharmacoresistant resected epilepsy samples obtained from Brodmann area 38. We established cut‐off criteria of |Log2 fold change| (|LogFC|) > 0.5 and an adjusted *p*‐value < 0.05. Then, a Volcano plot was generated using the negative Log10 of the adjusted *p*‐value against Log2FC. The plot was created utilising the ggplot2 package in R.

### 2.2 RNA‐Seq Data Acquisition and MeCp2, CDKL5 and BDNF Genes Co‐Expression Network Analysis

GSE139914 data was normalised using the DESeq2 package. We employed the weighted gene co‐expression network analysis (WGCNA) package in the R language to generate a co‐expression network for normalised genes. We aimed to identify co‐expressed genes in the physiological state; hence, we generated the co‐expression network for the transcriptomic data derived from autopsy (and not epileptic) samples. The co‐expression network was generated using parameters that have been described previously [26]. Briefly, we used the “blockwiseModules” function in the WGCNA package, setting TOMType = “unsigned”, power = 9 and mergeCutHeight = 0.25. The membership p‐value for each gene within the modules was calculated using the “cor” function in R. Next, we identified genes co‐expressed with MeCp2, CDKL5 and BDNF in the co‐expression network. We then examined whether any of the genes co‐expressed with MeCp2, CDKL5, or BDNF are differentially expressed between epileptic and control samples.

### 2.3 Gene Ontology (GO) Term Enrichment Analysis

We performed GO term enrichment analysis focusing on biological processes enriched for DEGs that were also co‐expressed with MeCp2, CDKL5 and BDNF. Genes were analysed utilising Cytoscape 3.9.1 with ClueGO version 2.5.9 plugin [27, 28]. The ClueGO options were set as GO biological process with a p‐value cut off = 0.05, the number of genes cut off = 3, kappa score cut off = 0.4 and percent of genes cut off = 4%. Moreover, the Enrich/depletion (two‐sided) hypergeometric test, Bonferroni step‐down *p*‐value correction and ClueGO grouping method were used.

### 2.4 Protein–Protein Interaction (PPI) Network Analysis

A PPI network was drawn for DEGs that were co‐expressed with MeCp2, CDKL5 and BDNF. To do this, we used String version 2.0.1 plugin in Cytoscape with a PPI score of > 0.4, Network type of “Full string Network” and “no additional interactors”. CytoHubba plugin version 0.1 in Cytoscape software which is a plug‐in that uses the Maximal Clique Centrality (MCC) algorithm to screen the hub genes was utilised to identify hub genes.

### 2.5 Human Brain Tissue Samples

Human epileptic hippocampal tissues were a portion of the brain tissues that were resected during the surgical intervention in Khatam Hospital, Tehran, Iran. All patients suffered from uncontrolled seizures and were considered intractable to antiepileptic drugs according to the definition of the International League Against Epilepsy (Table 1) [29]. All patients were evaluated by presurgical assessments and surgical resection of the temporal lobe was suggested to achieve seizure control. Informed consent was obtained from all patients. The study adhered to the ethical standards outlined in the 1964 Declaration of Helsinki, along with subsequent amendments. The local ethics committee of Shefa Neuroscience Research Center, Tehran, Iran approved the experimental protocol. Human autoptic hippocampal specimens were obtained during autopsies carried out on bodies from the body donor program of the Legal Medicine Organisation, Tehran, Iran.

**TABLE 1:** History and clinical features of patients with medically intractable epilepsy who underwent surgical intervention.

Code	Gender	Age	AEDs	Age of onset seizure (year)	Epilepsy duration (year)	Seizure frequency	Psychologic disorders	Engle	ILAE	Familial factor	Temporal lobe lesion side	Seizure duration
E1	Male	24	CBMZ+VPA + LEV	13	11	Weekly	No	4	5	No	Dominant	1–2 min
E2	Male	48	CBMZ+PHT + LTG + LEV	28	20	Monthly	No	1	1	No	Dominant	Less than 1 min
E3	Female	29	CBMZ+LEV+LTG	11	18	Weekly	No	1	1	No	Dominant	1–2 min
E4	Male	42	CBMZ+VPA	10	32	Weekly	No	1	1	Yes	Dominant	1–2 min
E5	Male	48	CBMZ+PHT + PHB	17	31	Weekly	Yes	4	5	No	Nondominant	1–2 min
E6	Female	39	CBMZ+LEV+LTG	4	35	Monthly	Yes	1	1	No	Dominant	1–2 min
E7	Male	34	CBMZ+VPA + TPA + PHB	9	26	Monthly	No	1	1	No	Dominant	Less than 1 min
E8	Female	41	LEV+PHB	8	33	Weekly	No	1	1	No	Dominant	Less than 1 min
E9	Male	30	CBMZ+VPA + PHT + PHB	12	18	Weekly	No	1	1	No	Dominant	Less than 1 min
E10	Male	52	CBMZ+VPA + TPA	10	42	Monthly	Yes	1	2	No	Dominant	Less than 1 min
E11	Female	33	LEV+LTG	0.5	33	Weekly	No	1	1	No	Dominant	1–2 min
E12	Female	39	CBMZ+LTG	11	28	Weekly	Yes	1	1	No	Nondominant	Less than 1 min
E13	Male	48	CBMZ+PHT + PHB	11	37	Monthly	No	1	1	No	Nondominant	Less than 1 min
E14	Male	33	CBMZ+LTG+ BZD	10	23	Weekly	Yes	3	4	No	Nondominant	1–2 min
E15	Female	13	CBMZ+VPA + TPA	3	10	Weekly	No	1	1	No	Dominant	Less than 1 min
E16	Male	28	CBMZ+PHT + PHB	10	18	Weekly	No	1	2	No	Dominant	Less than 1 min
E17	Male	24	CBMZ+VPA + TPA + PHB	0.5	25	Weekly	Yes	2	3	Yes	Nondominant	More than 5 min
E18	Female	29	LEV+PHT	12	17	Weekly	Yes	1	1	No	Nondominant	1–2 min
E19	Male	46	VPA + LTG	30	16	Monthly	Yes	3	4	No	Nondominant	2–5 min
E20	Female	18	CBMZ+LEV+ BZD + PHT	6	12	Weekly	No	1	1	No	Nondominant	Less than 1 min
E21	Female	33	LEV+TPA	1	32	Monthly	Yes	1	1	No	Nondominant	Less than 1 min
E22	Female	27	CBMZ+VPA + LEV+TPA	13	14	Weekly	Yes	3	4	No	Nondominant	Less than 1 min

The hippocampal tissues were obtained from 22 patients (34.5 ± 2.2 years) with intractable MTLE during surgical interventions. The patient's clinical features and history are summarised in Table 1. The control hippocampal tissues were obtained from the body donor program of the Legal Medicine Organisation, Tehran, Iran. The control subjects (*n* = 25; 34.9 ± 2.1 years) had no history of neurological or psychiatric diseases. The primary causes of their death were cardiac arrest (*n* = 8), multiple organ failure (*n* = 6), abdominal trauma (*n* = 6) and cardiorespiratory failure (*n* = 5). There were no significant differences in age and gender between epileptic patients and control subjects.

### 2.6 Total RNA Extraction and cDNA Synthesis

Total RNA was extracted from the epileptic hippocampal tissues and autoptic specimens via RNeasy Lipid Tissue Mini Kit (Qiagen, Germany) according to the manufacturer's instructions. DNase treatment was performed to eliminate the contamination of genomic DNA using RNase‐Free DNase Set (Qiagen, Germany). The total RNA was identified and quantified by a NanoDrop instrument (Thermo Scientific, USA). For cDNA synthesis, 500 ng of total RNA was used using oligo dT and random primers by reverse transcriptase of RevertAid First Strand cDNA Synthesis Kit (Thermo Fisher Scientific, Germany) and gel electrophoresis.

### 2.7 Quantitative Real‐Time PCR (qRT‐PCR)

The qRT‐PCR primers have been designed using Oligo7 and Primer Blast. The efficiency of qRT‐PCR was measured utilising LinRegPCR software. The primer information of the target genes and internal control are shown in Table 2. qRT‐PCR was conducted using HOT FIREPol EvaGreen qPCR Mix Plus (Solis BioDyne; Estonia) on the Bio‐Rad CFX96 System (Germany). To determine product specificity, a melting curve analysis was conducted at 65 to 95°C, by steps of 0.5°C every 5 s. Finally, data were analysed using the 2^−ΔΔCt^ (Livak) relative expression method. The competitive templates of the genes were normalised using the geomean of β‐Actin and HPRT genes as internal controls. PCR results from two patients were excluded from the final analyses because of the poor quality of the extracted RNA.

**TABLE 2:** List of primers for real‐time PCR analysis.

Gene	Sequence	PCR product length
CDKL5	5ˊ‐ACAGAGTACGTTGCCACCAG‐3ˊ	99 bp
	5ˊ‐AAGAATACAGCCCACCGACC‐3ˊ	
MeCp2	5ˊ‐GACCCATGTATGATGACCCCAC‐3ˊ	146 bp
	5ˊ‐CGCAATCAACTCCACTTTAGAGC‐3ˊ	
BDNF	5ˊ‐TCCCAAGGTCTAGGTGGAGG‐3ˊ	132 bp
	5ˊ‐GTAGGCACTTAAAGCACGAGG‐3ˊ	
β‐Actin	5ˊ‐AGGCGGACTATGACTTAGTTGCGTTACACC‐3ˊ	220 bp
	5ˊ‐AAGTCCTCGGCCACATTGTGAACTTTG‐3ˊ	
HPRT1	5ˊ‐GGACTAATTATGGACAGGACTG‐3ˊ	195 bp
	5ˊ‐GCTCTTCAGTCTGATAAAATCTAC‐3ˊ	

### 2.8 Western Blotting

Tissues were lysed with lysis buffer (ProteoJET Mammaalian Cell Lysis Reagent, and protease inhibitor) and mechanical digestion. Then, the solution was harvested through centrifugation (at 15,000 RPM, for 15 min at 4°C), and total protein concentrations were assessed according to the standard Bradford method. Samples (30 μg protein concentration) were separated utilising 12.5% polyacrylamide gel at 150 V and transferred to polyvinylidene difluoride (PVDF) membrane by a semidry tank. PVDF membranes were blocked and were incubated by anti‐MeCp2 (Abcam, USA), anti‐CDKL5 (Santa Cruz, Germany), anti‐BDNF (Santa Cruz, Germany) and anti‐β‐Actin (Cell Signalling, USA) antibodies as well as the secondary antibody (HRP conjugate) according to the manufacturer's instructions. Eventually, PVDF membranes were treated with 3,3′‐diaminobenzidine substrate solution, imidazole and cobalt chloride to detect target proteins [30]. Due to the low concentration of extracted protein, Western blots could not be performed for sample E20. Likewise, for sample E12 only CDKL5 and MeCp2 Western blots could be carried out (Table 1). Due to the low concentration of extracted protein, Western blots could not be performed for sample E20. Likewise, for sample E12 only CDKL5 and MeCp2 Western blots could be carried out (Table 1). Data analyses were performed using GelAnalyzer 2010a.

### 2.9 Statistical Analysis

GraphPad Prism 9 was utilised for statistical assays. Data outliers were identified based on the ROUT method. The Shapiro–Wilk test was used to test the normality of the data. Mann–Whitney *U*‐ or *t*‐test was used to compare means between two groups. Spearman's correlation test was performed to evaluate a relationship between expression values and Fisher's *R* to *Z* transformation test was carried out to compare the expression correlation between epileptic patients and control subjects [31]. Data are represented as mean ± SEM. *p* ≤ 0.05 was assumed as significant.

## 3 Results

### 3.1 Gene Expression in Control and Epileptic Samples

**FIGURE 1:**
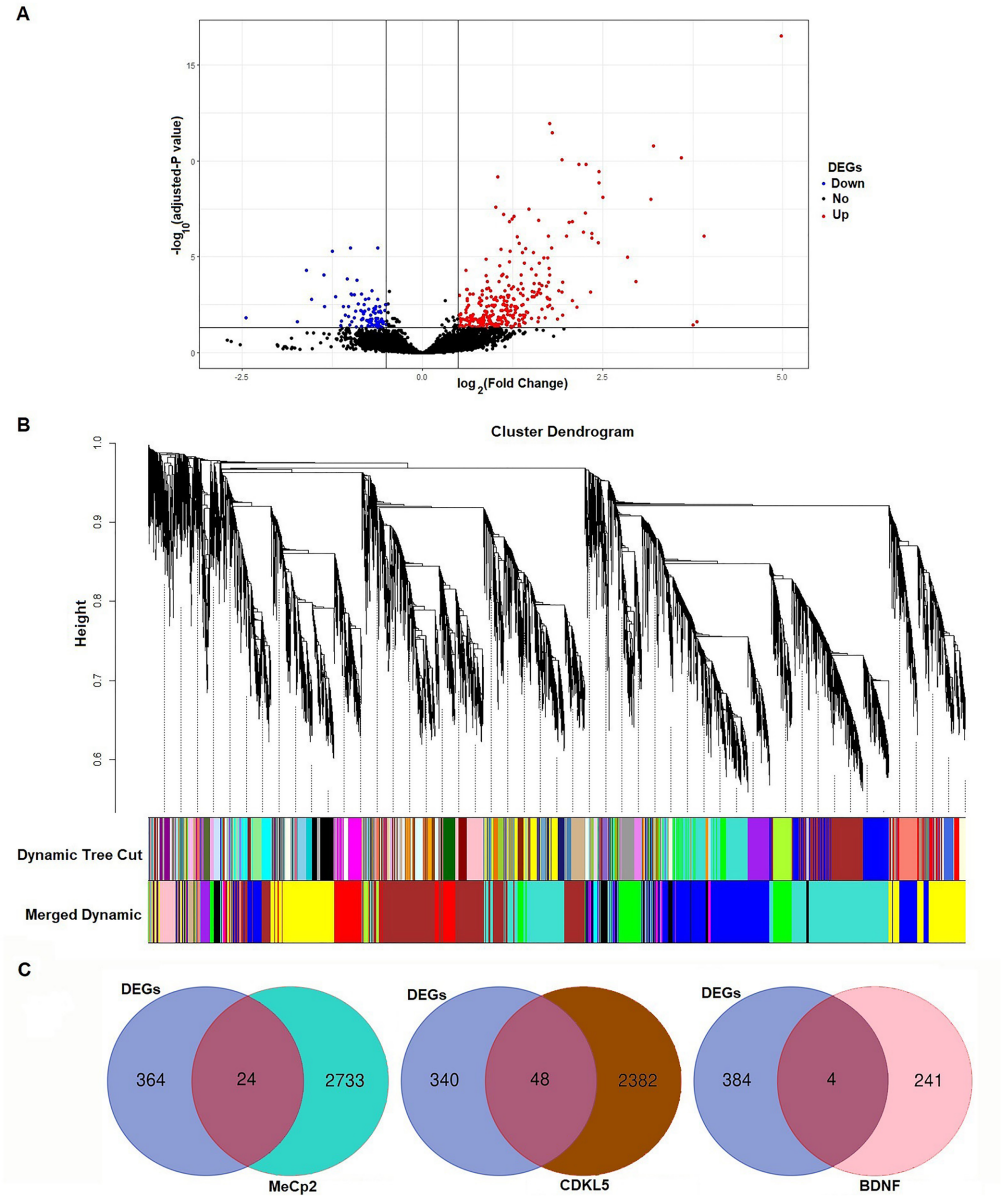
Deferential expression genes and co‐expression analysis. (A) Volcano plot of differentially expressed genes (DEGs). Red, upregulation; blue, downregulation; non‐altered genes: Black. The horizontal axis stands for log2‐fold change, and the vertical axis stands for adjusted p‐value (Adjusted *p*‐value < 0.05, |fold change| > 0.5). (B) Clustering dendrograms of gene expression of autoptic samples showing 14 modules that contain a group of co‐expressed genes. Genes co‐expressed with MeCp2, CDKL5, and BDNF were placed in the turquoise, brown, and pink modules, respectively. Each black vertical line corresponds to one gene. The colour‐coded rows below the dendrogram represent module membership (C) Venn diagrams show the count of co‐existing genes within the turquoise module (MeCp2 co‐expression genes), brown module (CDKL5 co‐expression genes), and pink module (BDNF co‐expression genes), alongside DEGs (*n* = 12).

Various strategies have been proposed to investigate the relationship between specific genes and clinical conditions, such as analysing the frequency of gene variants in affected individuals or performing gene expression analyses. Given that MeCP2, CDKL5 and BDNF regulate a broad array of genes, we focused on identifying whether genes co‐expressed with these regulators show altered expression in epilepsy by intersecting them with genes exhibiting differential expression. This approach allows us to identify potential genes that may be involved in the epileptic phenotype through shared molecular pathways with MeCP2, CDKL5 and BDNF. We used batch 1 of the GSE139914 dataset, including 12 epileptic and 12 healthy autoptic samples, to obtain DEGs (Figure S1). In the total of 388 DEGs, 299 upregulated and 89 downregulated DEGs were identified between epilepsy patients and controls (Tables S1 and S2). A volcano plot of the expression values is shown in Figure 1A.

Furthermore, to identify co‐expressed genes with MeCp2, CDKL5 and BDNF in the temporal lobe, we constructed a co‐expression network utilising autoptic sample data and the WGCNA package. Following the merging process, all genes were sorted into 14 modules. However, 24 genes were not assigned to any specific module and were categorised under the grey module (Figure 1B; Table S3). We conducted a more in‐depth analysis of gene co‐expression within each module. Genes exhibiting co‐expression with MeCp2, CDKL5 and BDNF were assigned to the turquoise, brown and pink modules, respectively (Figure 1C). There are 24, 48 and 4 common genes between MeCp2, CDKL5 and BDNF with DEGs, respectively (Figure 1C), which makes a total of 76 genes (Table S4).

### 3.2 GO Term Enrichment Analysis

**FIGURE 2:**
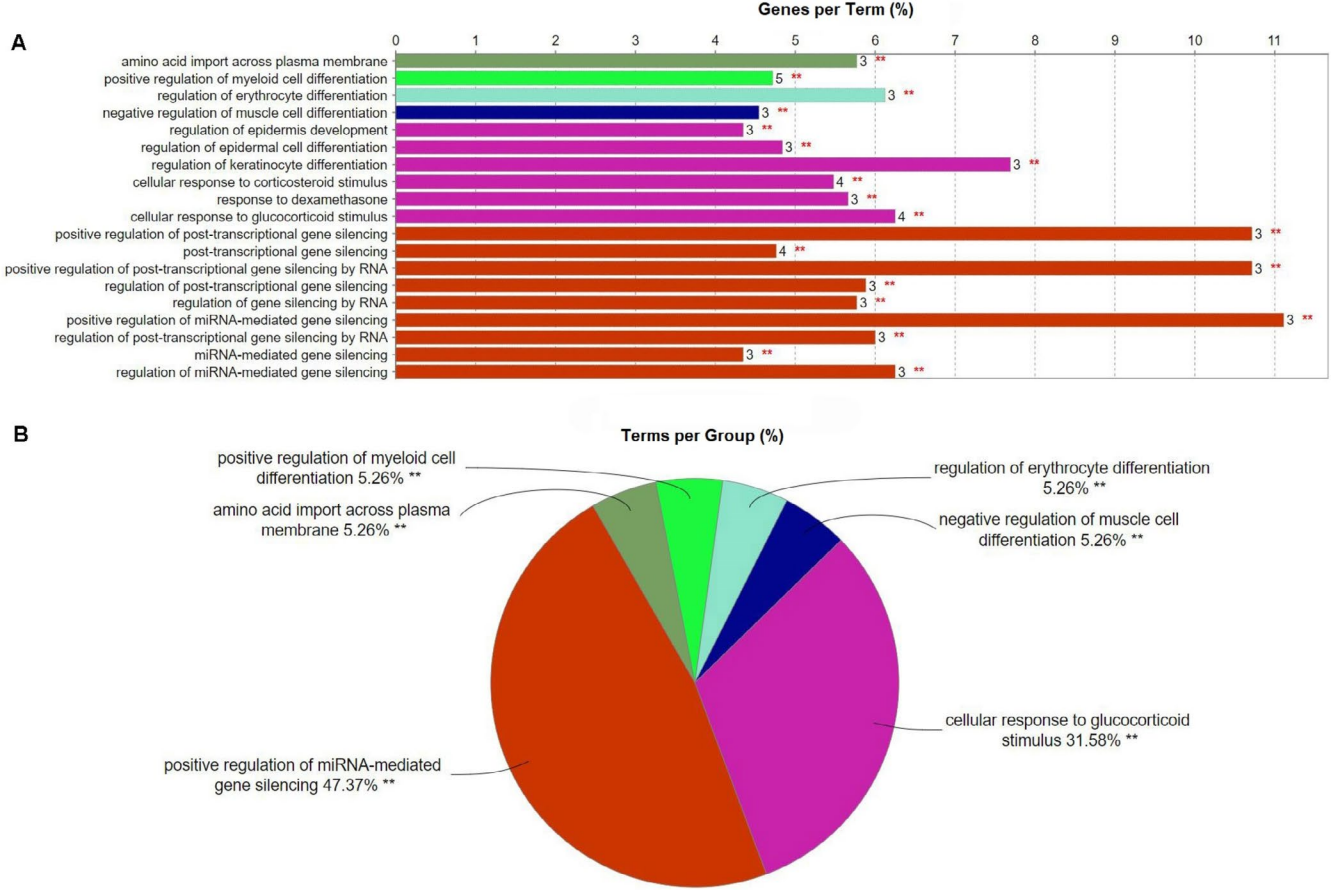
(A) Gene Ontology (GO) biological process enrichment analysis depicting the percentage of terms per ClueGO group for co‐existing MeCp2, CDKL5, and BDNF co‐expression genes and DEGs. Significance level: **p < 0.05 for group cluster test. (B) Percentage of genes per term (; **p < 0.05 for each single pathway test).

GO term enrichment analysis was used to identify biological processes associated with MeCp2, CDKL5 and BDNF genes. Building upon these genes along with their co‐expressed and DEGs, we performed analysis utilising the ClueGO plugin within the Cytoscape software. GO term enrichment analysis with a *p*‐value threshold of less than 0.05 revealed several significant biological process association (Figure 2A,B and Figure S2). Among the enriched processes, the positive regulation of myeloid cell differentiation contributes to immune and haematopoietic regulation and potentially influences the pathogenesis of chronic temporal lobe epilepsy [32]. Moreover, processes related to amino acid import across the plasma membrane highlight potential roles in cellular nutrient uptake and metabolic regulation. In an epileptic brain, dysregulation of amino acid transport can disrupt the balance of excitatory and inhibitory signals, leading to neuronal hyperexcitability and contributing to epileptogenesis and seizure propagation [33]. These genes were also associated with cellular responses to corticosteroid and glucocorticoid stimuli, including dexamethasone, indicating their involvement in cognitive function, inflammatory signalling and the regulation of brain excitability. In addition, the regulation of post‐transcriptional gene silencing was also enriched, indicating a potential role in RNA‐based regulatory mechanisms that control gene expression [34]. These findings suggest that disturbances in the aforementioned biological processes are likely contributing to intractable epilepsy, potentially due to the dysregulation of MeCp2, CDKL5 and/or BDNF.

### 3.3 PPI Analysis of Co‐Expressed Genes Involving MeCp2, CDKL5 and BDNF

**FIGURE 3:**
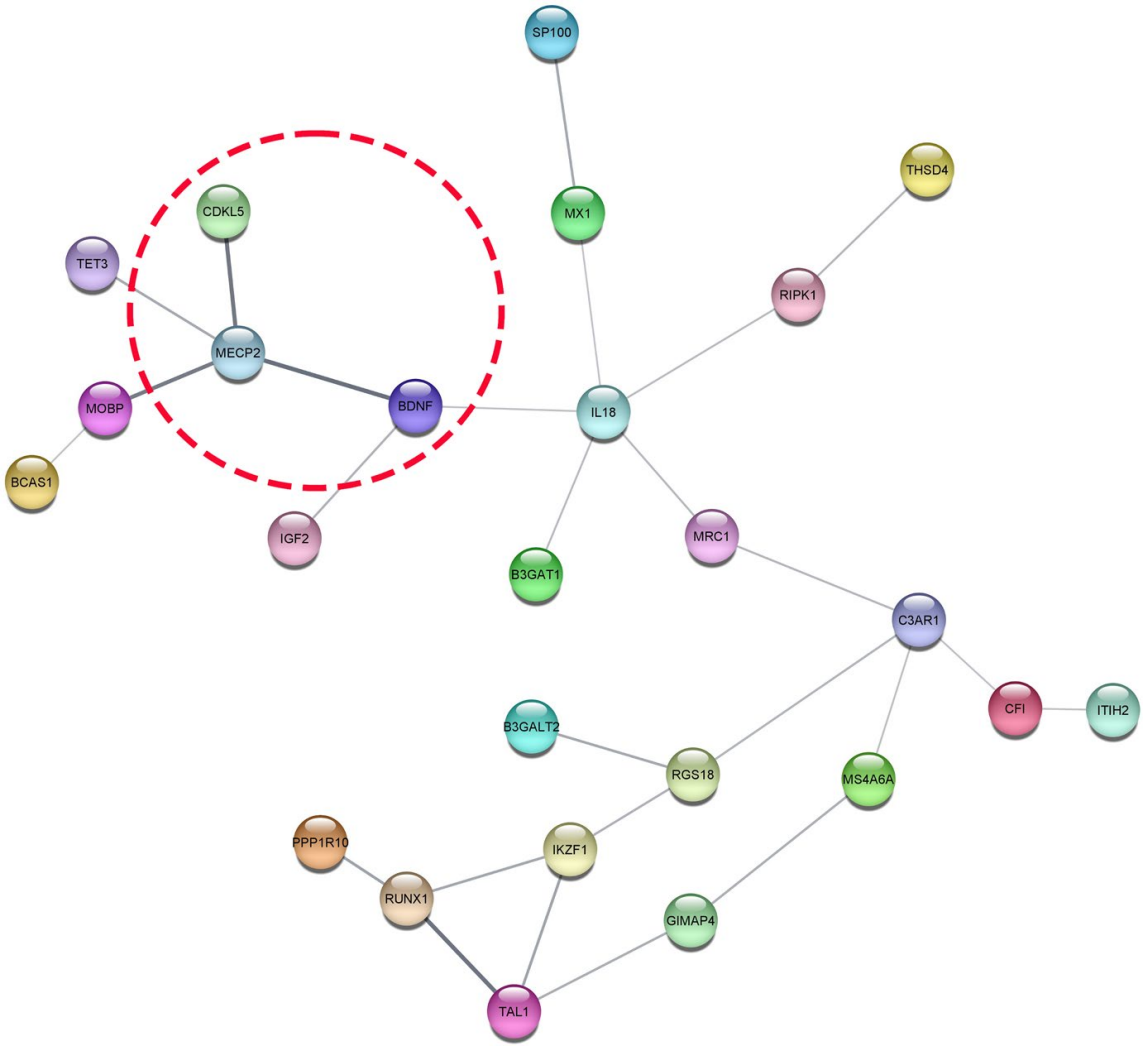
Protein–protein interaction networks depicting the co‐existing of MeCp2, CDKL5, and BDNF co‐expression genes and DEGs, Nodes disconnected from the main network are not shown.

The protein–protein interaction (PPI) network of MeCP2, CDKL5, BDNF and their co‐expressed DEGs was analysed using Cytoscape. Figure 3 illustrates a network of 25 nodes and 26 edges, showing the interactions and relationships among the identified genes. Key interactions include *MeCP2* with *CDKL5*, *BDNF*, *MOBP* and *TET3*, while *BDNF* interacts with *MeCP2*, *interleukin (IL)‐18* and *IGF2*. Hub genes were identified using MCC scores in the CytoHubba plugin. The analysis highlighted *IL‐18* and *MeCP2* as top hub genes with MCC scores of 5 and 4, respectively (Table S5). It has been suggested that the pro‐inflammatory effects of *IL‐18* contribute to the development and progression of epilepsy, as well as the associated structural damage [35]. Furthermore, the hub genes *C3AR1*, *RUNX1*, *IKZF1* and *TAL1* were identified as significant nodes, each with MCC scores of 3. These genes are potentially pivotal in immune regulation, haematopoiesis and transcriptional control, processes that are increasingly recognised for their influence on epileptogenesis [36, 37, 38, 39].

### 3.4 The mRNA Levels of MeCp2, CDK5 and BDNF

**FIGURE 4:**
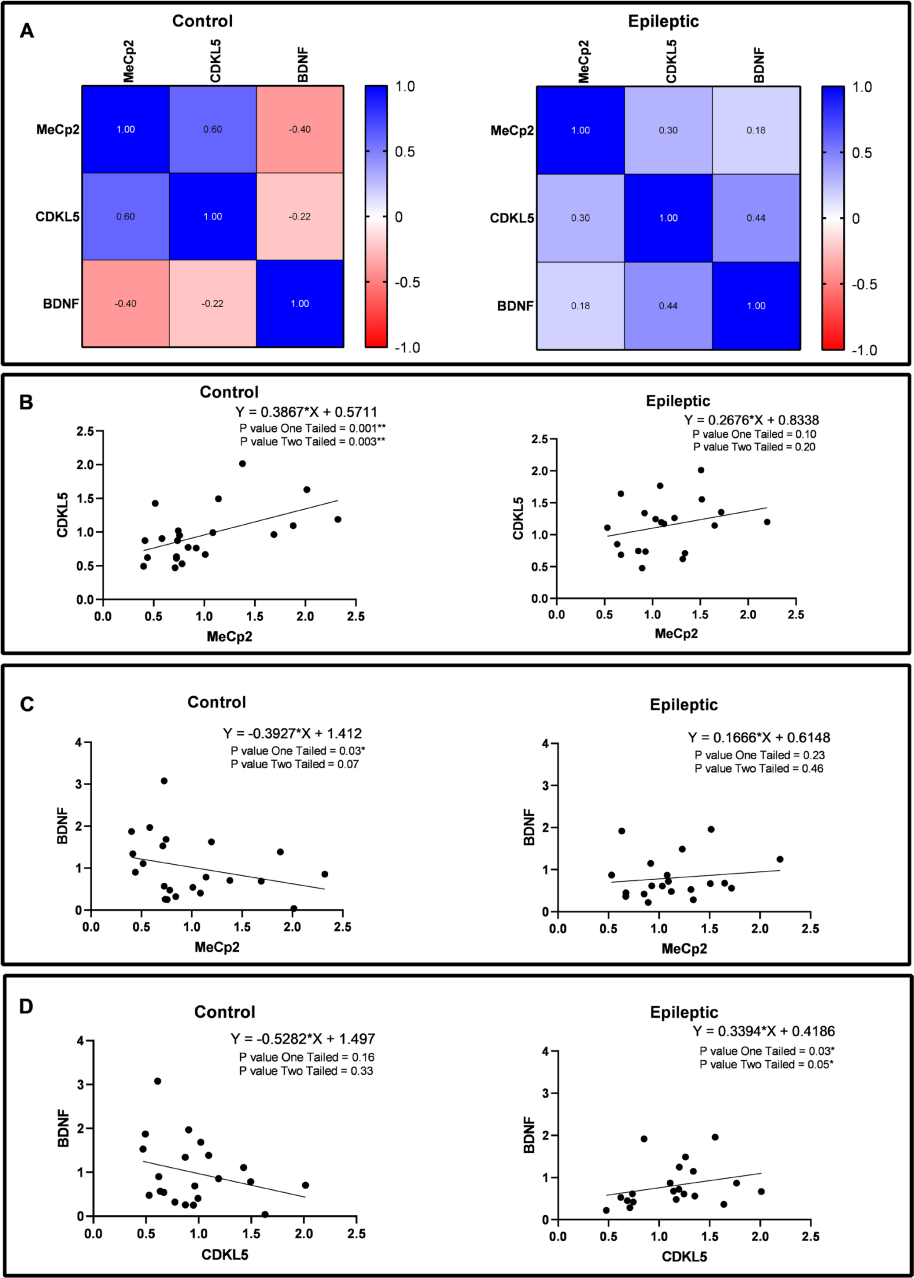
Pairwise comparisons of the MeCp2, CDKL5, and BDNF gene expression in the hippocampal specimens obtained from epileptic patients and autoptic controls. (A) The heatmap of correlation between the expression of MeCp2, CDKL5, and BDNF in the hippocampal tissues obtained from autoptic controls and epileptic patients. Correlation between the expression of MeCp2 and CDKL5 genes (B), MeCp2 and BDNF (C), and CDKL5 and BDNF (D) in the hippocampus of autoptic controls and epileptic patients (*n* = 20–23).

The mRNA expressions of *MeCp2*, *CDKL5* and *BDNF* genes in the epileptic hippocampal tissues and control specimens were assessed. No significant differences have been observed between the mRNA expression of these genes in the epileptic and control groups (Figure S3). However, various significant correlations have been observed between the expressions of these genes in the epileptic and/or control hippocampal specimens. The results of correlation analysis indicated a positive correlation between mRNA expression of MeCp2 and CDKL5 in the autoptic hippocampal samples (*r* = 0.6, *p* = 0.001; Figure 4A,B). Moreover, there was a negative correlation between the mRNA expression of MeCp2 and BDNF (*r* = −0.22, *p* = 0.03 Figure 4A,C) in control hippocampal tissues and, a positive correlation between the mRNA expression of CDKL5 and BDNF (*r* = 0.44, *p* = 0.03; Figure 4A,D) in epileptic hippocampal tissues. Using the R to Z transform Fisher test, the correlation patterns of the mRNA expression between the epileptic and control tissues were compared. We found significant differences in the mRNA expression correlation pattern of MeCp2 and BDNF between epileptic and control hippocampal samples (*p* = 0.04; Table 3). Furthermore, a significant difference was observed in the correlation pattern of the mRNA expression of CDKL5 and BDNF between the epileptic and control hippocampal samples (*p* = 0.02; Table 3).

**TABLE 3:** Pairwise comparisons of the BDNF, CDKL5, and MeCp2 gene and protein expression in the hippocampus of epileptic patients and autoptic controls.

	Marker a	Marker b	*Z*	*p* (one tailed)	*p* (two tailed)
Gene expression	BDNF	CDKL5	−2.07	0.0192	0.0385
	BDNF	MeCp2	−1.79	0.0367	0.0735
	CDKL5	MeCp2	1.18	0.119	0.238
Protein expression	BDNF	CDKL5	−0.47	0.3192	0.6384
	BDNF	MeCp2	0.4	0.3446	0.6892
	CDKL5	MeCp3	0.49	0.3121	0.6241

*Note:* The data of the *R* to *Z* transformation test are presented.

### 3.5 The Expression of MeCp2, CDKL5 and BDNF at the Protein Levels

**FIGURE 5:**
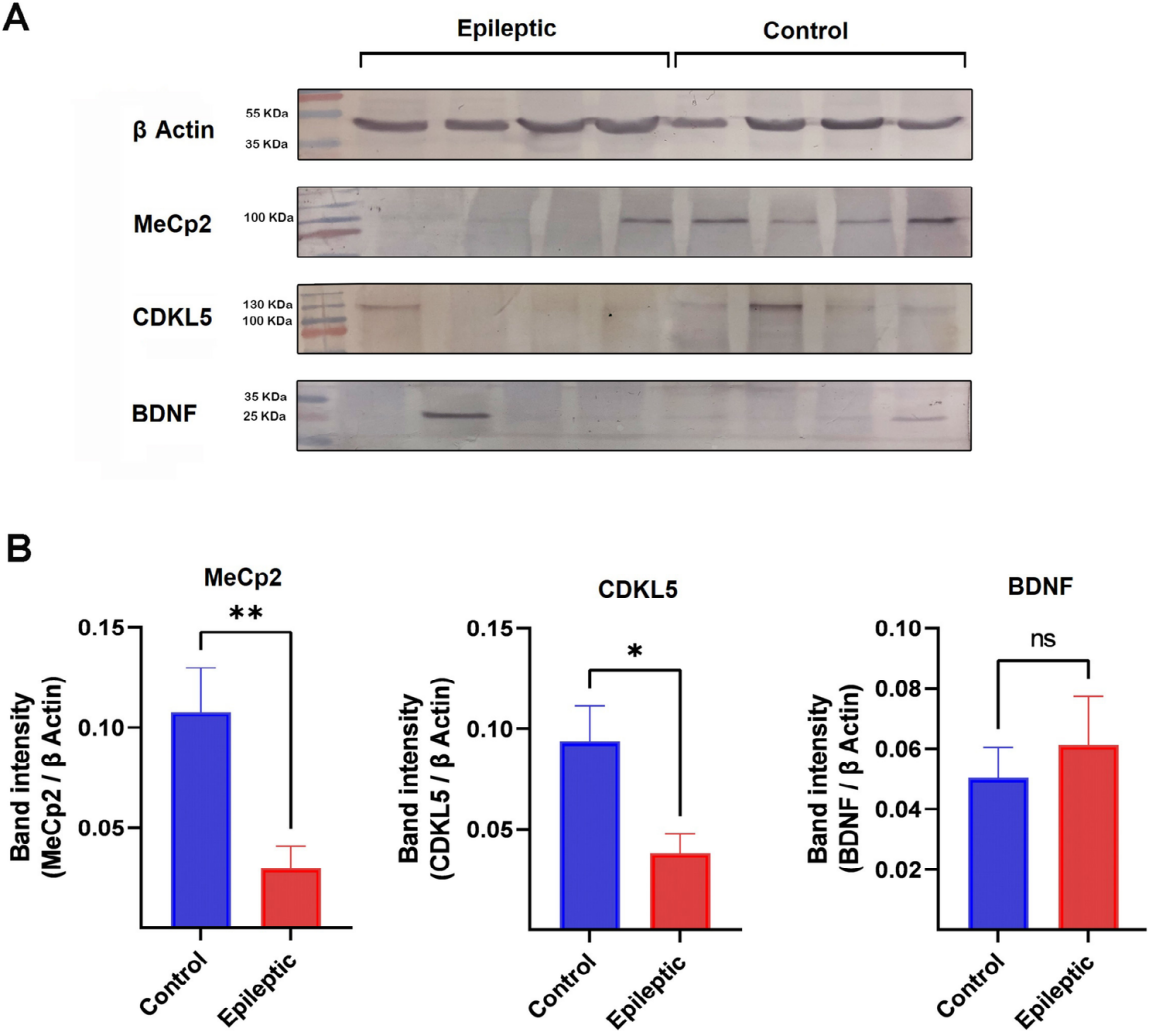
Western blotting analysis of MeCp2, CDKL5, and BDNF in the hippocampal tissues of epileptic patients and autoptic controls. (A) Representative images of western blotting for MeCp2, CDKL5, BDNF, and its respective loading control protein β‐Actin for control and epileptic groups are shown. (B) Bar diagrams represent the band intensity of MeCp2, CDKL5, and BDNF protein values of epileptic and control hippocampal tissues. Each bar represents the mean ± SEM. *, **, and ns indicate *p* ≤ 0.05, *p* ≤ 0.01, and not significant, respectively (*n* = 18–25).

**FIGURE 6:**
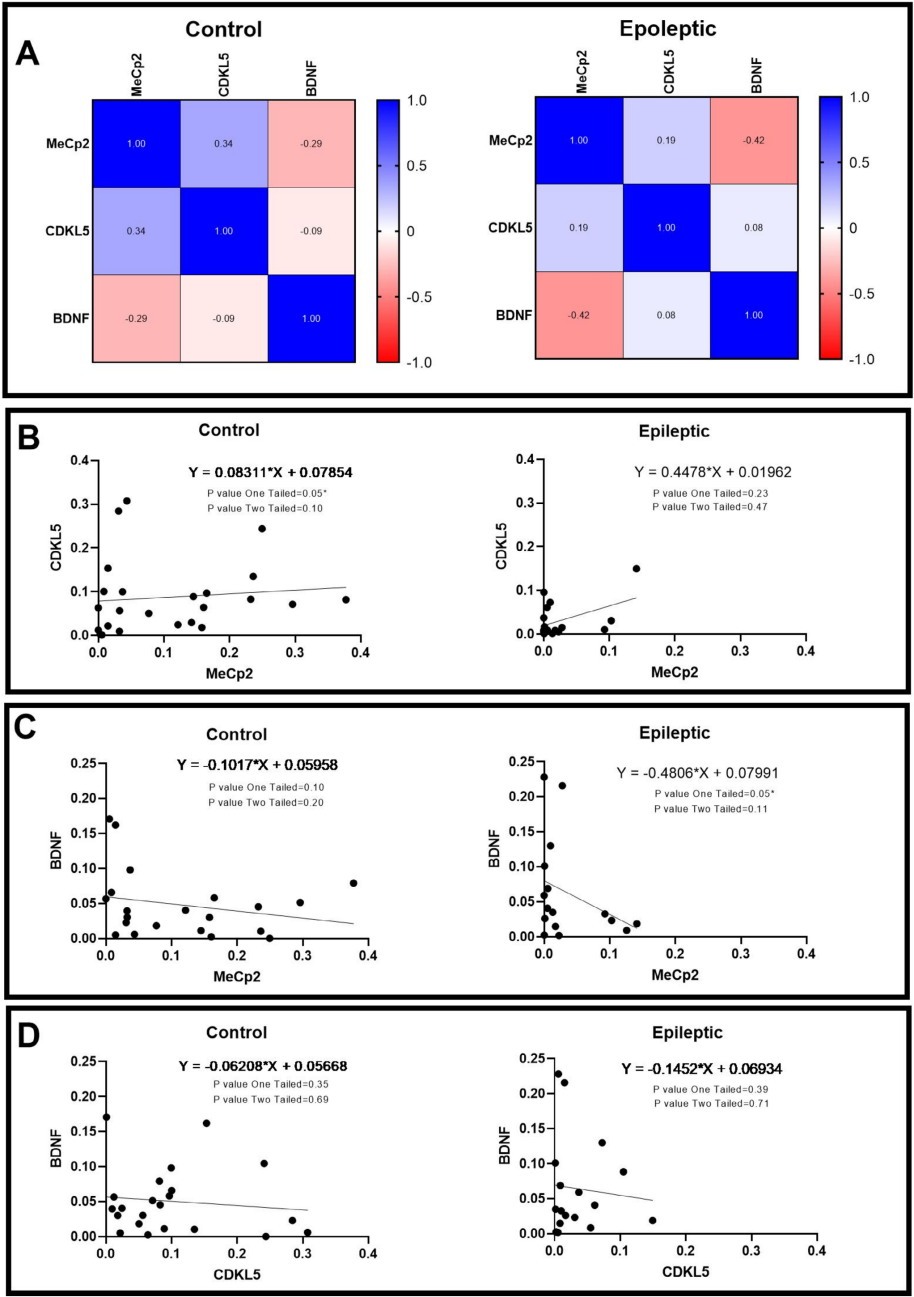
Pairwise comparisons of the MeCp2, CDKL5, and BDNF protein expression in the hippocampal tissues obtained from epileptic patients and autoptic controls. The heatmap of correlation between the protein expression of MeCp2, CDKL5, and BDNF in the hippocampal tissues obtained from autoptic controls and epileptic patients (A). Correlation between the protein expression of MeCp2 and CDKL5 (B), MeCp2 and BDNF (C), and CDKL5 and BDNF (D) in the hippocampus of autoptic controls and epileptic patients. * Indicate *p* ≤ 0.05 (*n* = 18–25).

Western blot analysis was used for detecting protein expression of MeCp2, CDKL5 and BDNF in the epileptic and control hippocampal specimens. Our findings revealed a significant reduction in MeCp2 values in the epileptic hippocampus compared to the control tissues (*p* = 0.01; Figure 5A,B). Moreover, there was a significant reduction of the CDKL5 protein expression levels in the hippocampal tissues of epileptic patients compared to the control group (*p* = 0.05; Figure 5A,B). The correlation analysis revealed a significant positive correlation between MeCp2 and CDKL5 protein expression in the control hippocampal tissues (*r* = 0.34, *p* = 0.05; Figure 6A,B). Furthermore, a significant negative correlation between MeCp2 and BDNF protein expression in the epileptic hippocampal tissues (*r* = −0.42, *p* = 0.05; Figure 6A,C). There were no significant differences in the correlation patterns of other studied proteins between the epileptic samples compared to the controls (Figure 6A–D).

### 3.6 Clinical Relevance of Changes in Gene Expression

**FIGURE 7:**
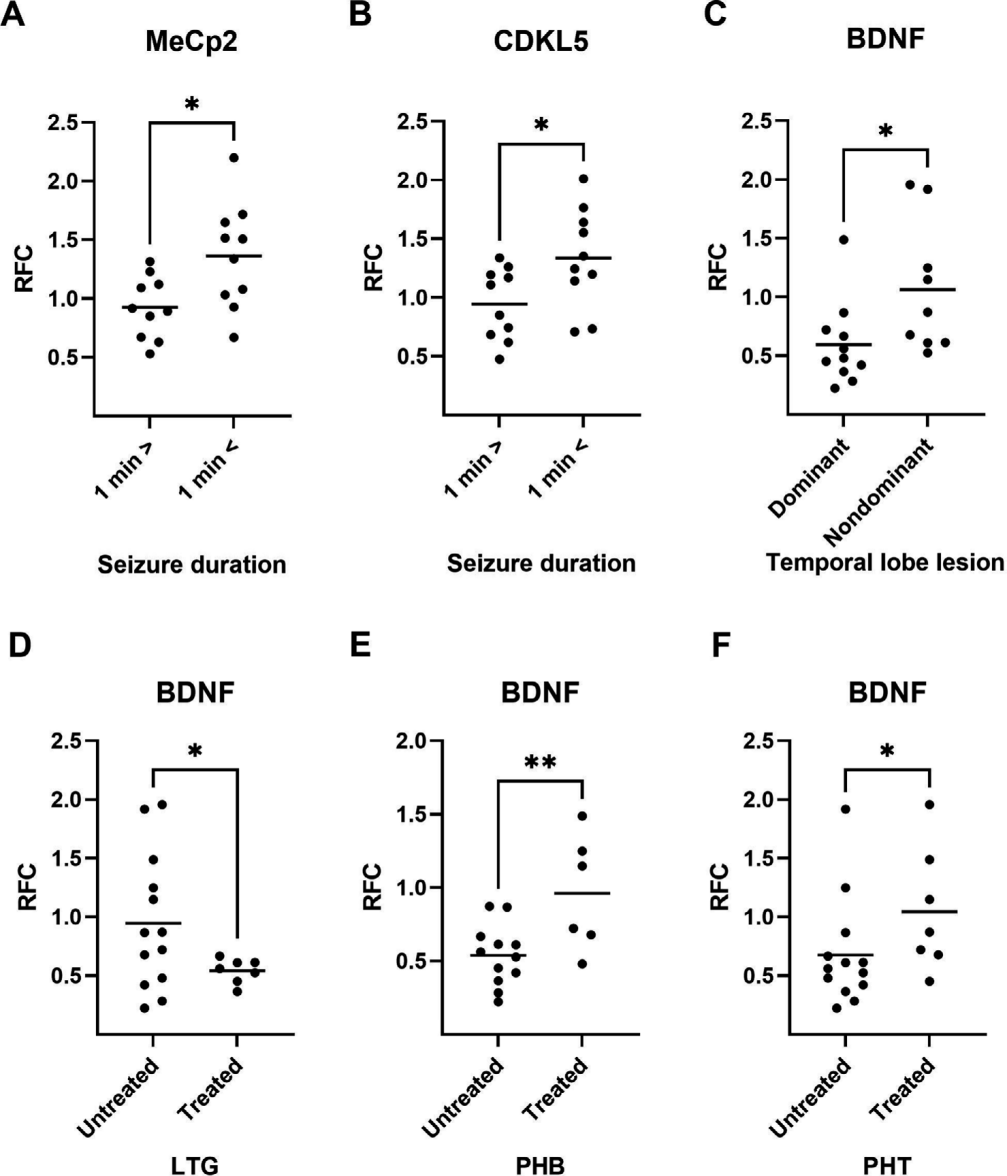
The effect of anticonvulsant therapy and seizure duration on the mRNA expression of MeCp2 and BDNF gene profiles in the epileptic hippocampus. (A, B) The mRNA expression level of MeCp2 and CDKL5 was significantly greater in patients with a seizure duration of less than 1 min compared to subjects with seizures longer than 1 min. (C–F) The mRNA expression of BDNF was significantly different in patients treated with lamotrigine (LTG), phenobarbital (PHB), and phenytoin (PHT) than in subjects who received other drugs. * and ** indicate *p* ≤ 0.05 and *p* ≤ 0.01, respectively (*n* = 18–20).

We evaluated the potential association between the expression values of MeCp2, CDKL5 and BNDF in the epileptic hippocampus and the clinical characteristics of patients. The mRNA expression of the *MeCp2* and *CDKL5* genes was significantly higher in patients with seizure duration of > 1 min than in subjects with seizures shorter than 1 min (Figure 7A,B). Furthermore, the level of mRNA expression of *BDNF* in patients with brain lesions located in the dominant site was significantly lower than in subjects with non‐dominant hemisphere (*p* ≤ 0.05; Figure 7C) Anti‐epileptic drugs may increase the risk factors affecting epigenetic molecular mechanisms, such as DNA methylation [40]. Our analysis revealed that the mRNA expression of *BDNF* in the hippocampus of epileptic patients treated with lamotrigine was significantly lower than in subjects who received other drugs (*p* ≤ 0.05; Figure 7D). Furthermore, the mRNA levels of *BDNF* in the hippocampus of the patients treated with phenobarbital (*p* ≤ 0.01) or phenytoin (*p* ≤ 0.05) were significantly higher than patients treated with other drugs (Figure 7E,F).

**FIGURE 8:**
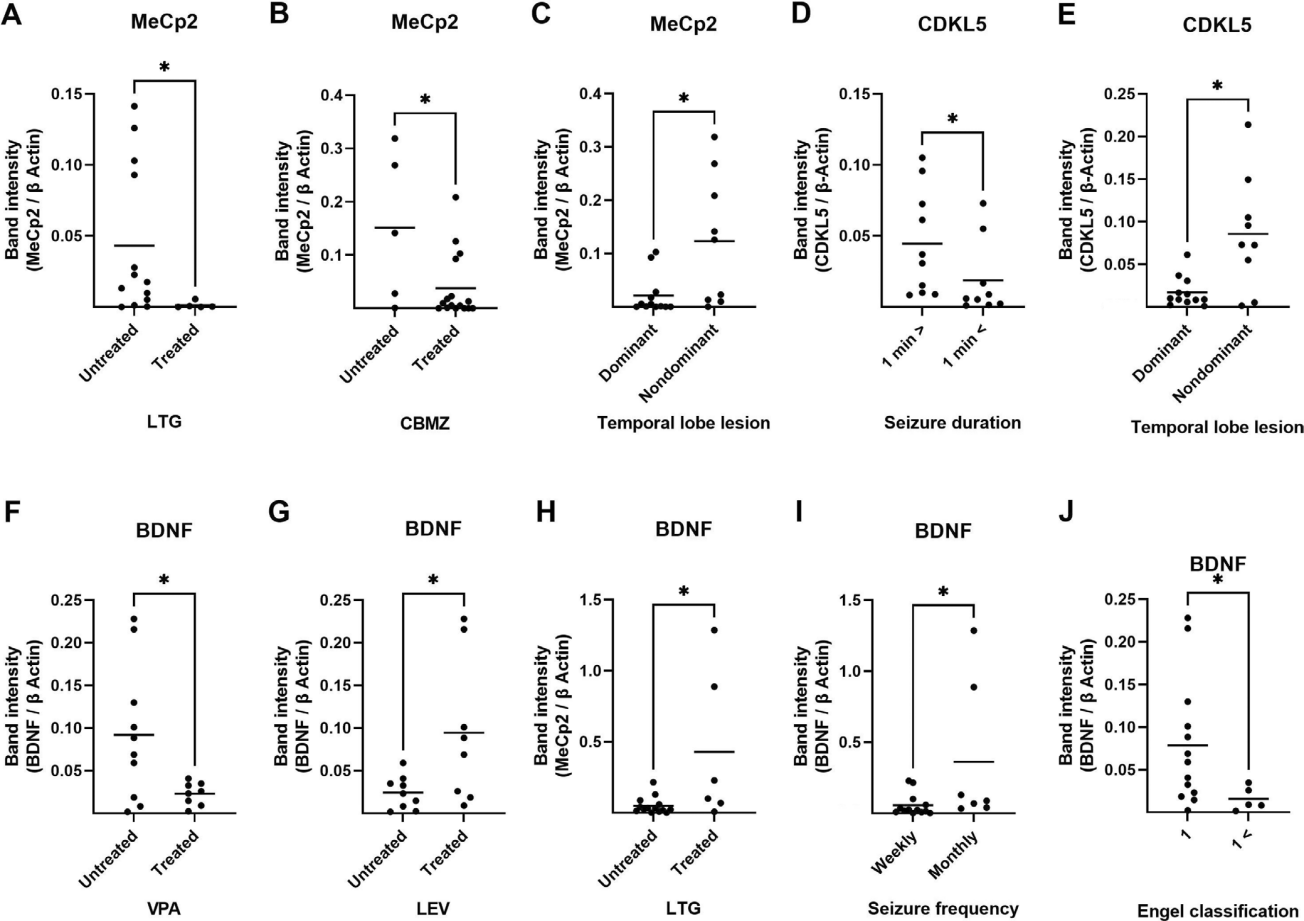
The comparison between the protein expression of MeCp2, CDKL5, and BDNF in the epileptic hippocampus with different clinical features. Association of MeCp2 protein expression in the epileptic hippocampus with the application of lamotrigine and Carbamazepine (LTG; A, CBMZ; B), and the location of the brain lesion (Dominant vs. non‐dominant hemisphere; C) are presented. The expression of CDKL5 in the epileptic hippocampus is significantly associated with seizure frequency (D), and the location of the brain lesion (E). BDNF protein expression in the epileptic hippocampus has a significant association with the application of valproate (F), levetiracetam (LEV; G), and LTG (H), as well as with seizure frequency (I) and outcomes of epilepsy surgery (J). Note that the administration of LTG and long‐term outcomes of epilepsy surgery have a similar correlation pattern with the expression of MeCp2 and BDNF. * indicates *p* ≤ 0.05 (*n* = 17–21).

A significantly lower protein expression of MeCp2 in the hippocampus has been observed in patients receiving lamotrigine and carbamazepine compared to subjects treated with other drugs (*p* ≤ 0.05; Figure 8A,B). Moreover, the level of MeCp2 and CDKL5 protein expression in patients with brain lesions located in the dominant site was significantly lower than in subjects with lesions in the non‐dominant hemisphere (*p* ≤ 0.05; Figure 8C,E). Patients with seizure duration of < 1 min exhibited significantly lower protein values of CDKL5 in the hippocampus compared to subjects with longer seizure duration (*p* ≤ 0.05; Figure 8D). Moreover, the hippocampal expression level of BDNF protein was significantly lower in subjects who received valproate than in patients who received other drugs (*p* ≤ 0.05; Figure 8F). Patients treated with lamotrigine and levetiracetam exhibited higher levels of BDNF protein expression than individuals who received other anticonvulsants (*p* ≤ 0.05; Figure 8G,H). The value of BDNF protein expression in the hippocampus was significantly lower in patients with weekly seizures compared to individuals with monthly seizures (*p* ≤ 0.05; Figure 8I). A higher expression of BDNF in the epileptic hippocampal specimens is also significantly associated with better outcomes of epilepsy surgery (*p* ≤ 0.01; Figure 8J).

## 4 Discussion

The fate of newly generated neurons, synaptic structures, neurotransmitter synthesis and protein abundance of ion channels are tightly regulated by epigenetic mechanisms [41, 42]. The role of pathogenic variants of MeCp2 and CDKL5 in epileptogenesis is complex and still not fully understood. The impaired function of MeCp2 leads to extensive transcriptional disruptions and affects GABAergic and glutamatergic synaptic transmission, axon guidance, calcium signalling pathways and inflammatory processes [43]. Several experimental studies have shown that MeCp2 dysfunction leads to a reduction in GABA concentration, a decrease in GAD_65_ and GAD_67_ protein levels and GABA uptake, as well as enhancement of glutamate release, induction of oxidative damage and dysfunction of cholinergic neurons [44, 45, 46, 47]. MeCp2 perturbation alters excitation/inhibition balance in the hippocampal network through increased tonic inhibition in the thalamocortical system and reduced synaptic GABA_A_ receptor activation, which could lead to neural circuit hyperexcitability and the occurrence of epileptiform burst potentials [46]. Furthermore, spike–wave discharges could be triggered by MeCp2‐induced changes in the neuronal membrane surface potential and a consequent abnormal negative shift in the voltage‐dependencies of hyperpolarisation‐activated cyclic nucleotide‐gated and Ca^2+^ channels in the hippocampal neurons [48]. There are several similarities between the pathological role of MeCp2 and CDKL5 mutations in CNS. CDKL5 mutations lead to reductions in dendritic arborization and spine density, alteration of synaptic morphology, dysregulation in the expression of glutamate AMPA sub‐receptors, and dysfunction of voltage‐gated ion channels [49, 50]. Dysregulations of the expression of GluN2B in postsynaptic densities with irregular Ca^2+^ permeability result in greater seizure susceptibility in CDKL5 knockout mice [51].

Our bioinformatics findings indicate that MeCp2, CDKL5 and BDNF genes are implicated in the regulation of genes associated with epilepsy and disruptions in these genes may play a contributory role in the development of epilepsy. Furthermore, our study revealed a significant reduction in protein expression of MeCp2 and CDKL5 as well as an altered correlation between BDNF, MeCp2 and CDKL5 expression in the hippocampus of patients with MTLE. These results indicate a link between MeCp2 and CDKL5 expression and support the possible involvement of these two proteins in the same molecular pathways underlying seizures in MTLE. Previous studies suggest a common molecular link between MeCp2 and CDKL5 in Rett syndrome, which is responsible for the early‐onset seizure variant of this disorder [19], presumably via interaction with the DNA‐methyltransferase 1 enzyme [52]. MeCp2 is a target molecule for the regulation of DNA methyltransferase activity [53]. MTLE patients with hippocampal sclerosis have shown increased DNA methyltransferase values [54] and enhanced methylation in gene promoters [55] in the hippocampus. Different correlations between MeCp2 and CDK5 in control and epileptic hippocampal tissues in our study suggest the possible disruption in the regulatory mechanisms of gene expression at the mRNA level in epileptic patients. MeCP2 and CDKL5 play a coordinated role with Reelin‐dependent signalling in the laminar structure of neurons as well as in synaptic neurotransmission in the hippocampus [56, 57] Dysfunction of the gene encoding the secreted protein Reelin has been reported in patients with some epilepsy syndrome, such as autosomal dominant lateral temporal epilepsy [58]. A complex malfunction among MeCP2, CDKL5 and Reelin in dysregulating the extracellular matrix protein‐encoding genes may contribute to epilepsy [59].

In keeping with a previous study [20], our findings revealed a discrepancy between the expression of MeCp2 and CDKL5 at the mRNA and protein levels, probably caused by the post‐transcriptional regulatory mechanisms. Combinatorial post‐transcriptional circuitry, implicating differential modulation of RNA‐binding proteins and miRNAs, controls MeCp2 protein levels in neuronal tissues [60]. MeCp2 could repress transcriptional initiation via the interaction of intragenic enhancers with promoters [61]. However, it should be noted that an enhancement of the mRNA and protein expression of MeCp2 has been observed in the temporal neocortex of patients with MTLE [25]. It has been shown that DNA methylation alterations are more striking in the neocortex than in the hippocampus of patients with MTLE [62]. We observed significant changes in the mRNA expression correlation patterns of MeCp2 and CDKL5 with BDNF between epileptic and autoptic hippocampal samples. In keeping with our results, it has been shown that MeCp2 differentially regulates BDNF transcription in normal and epileptic conditions via the modulation of the specific phosphorylation of the methyl‐binding protein [63]. CDKL5 also plays a modulatory role in the expression of the BDNF gene [64].

Our data revealed that altered mRNA or protein expression of MeCp2, CDKL5 and/or BDNF was correlated with the seizure duration and frequency, the outcome of surgery, localization of the seizure onset zone, or the use of various antiepileptic drugs. Different patterns of DNA methylation in experimental epilepsy models [65] and human focal cortical dysplasia [66] are associated with seizure phenotype and aetiology. DNA methylation could contribute to the frequency and intensity of seizures [67]. Experimental studies indicate that the pattern of DNA methylation alteration relies on the epileptogenic aetiology and the stage of seizure development [65]. Furthermore, it has been shown that various mutations of MeCp2 can modulate the frequency of seizures, seizure types and disease severity in patients with Rett syndrome [68].

Several studies suggest that antiepileptic drugs modulate DNA methylation, influencing the expression of genes associated with epilepsy pathogenesis, therapeutic outcomes and neurotrophic factors. These epigenetic changes can impact pathways involved in neuronal excitability, synaptic plasticity and inflammatory signalling. A DNA methylation signature may serve as a biomarker for MTLE and, when combined with clinical data, enhance predictions of anticonvulsant treatment response [69]. Valproate and lamotrigine induce specific regions of DNA methylation in epileptic patients, presumably via their effects on micronutrients [70]. Valproate induces broad epigenetic alterations in CNS through various mechanisms, mainly DNA demethylation and histone acetylation [71]. Furthermore, clinical and experimental studies revealed significant changes in both mRNA and protein BDNF levels after lamotrigine, phenobarbital, phenytoin and valproate application [72, 73, 74, 75]. The effect of anticonvulsants like valproate on BDNF expression appears to be influenced by the severity and nature of pathological changes, as well as cognitive dysfunction, with a significant negative correlation observed between BDNF mRNA levels and the percentage of methylated DNA in the hippocampus of valproate‐treated subjects [76, 77]. Understanding the role of anticonvulsants in DNA methylation could aid in identifying epigenetic biomarkers for treatment response and provide new therapeutic targets to enhance drug efficacy or develop innovative treatments [78]. Furthermore, the observed discrepancy between RNA and protein levels for MeCP2 and CDKL5 in this study may result from post‐transcriptional regulation by miRNAs. TargetScan analysis of their 3’ UTR sequences identifies multiple miRNA‐binding sites, suggesting translational suppression rather than mRNA degradation as a potential mechanism for reduced protein levels [79, 80]. This finding highlights the need for further studies to explore the role of miRNA‐mediated regulation in modulating protein expression in this context.

In conclusion, we have identified alterations in the values of MeCp2, CDKL5 and BDNF, as well as their correlation patterns, in the hippocampus of patients with MTLE. Furthermore, various correlation patterns between changes in the expression of these genes and clinical characteristics have been observed. These alterations might represent a part of the pathogenic and/or compensatory responses in patients with MTLE. This study enhances our understanding of epilepsy by identifying novel potential diagnostic and prognostic biomarkers, as well as therapeutic targets.

## Author Contributions

**NoorMohammad Meshkinkhood:** conceptualization (supporting), data curation (lead), formal analysis (lead), methodology (lead), visualization (lead), writing – original draft (supporting). **Parastoo Barati Dowom:** investigation (supporting), methodology (supporting). **Farshid Noorbakhsh:** validation (equal), writing – review and editing (supporting). **Masoud Ghadipasha:** investigation (supporting), methodology (supporting). **Jaber Gharehdaghi:** investigation (supporting), methodology (supporting). **Christoph Kellinghaus:** investigation (supporting), writing – review and editing (supporting). **Erwin‐Joseph Speckmann:** investigation (supporting), writing – review and editing (supporting). **Maryam Khaleghi Ghadiri:** investigation (supporting), writing – review and editing (supporting). **Walter Stummer:** investigation (supporting), writing – review and editing (supporting). **Ali Gorji:** conceptualization (lead), supervision (lead), validation (lead), writing – review and editing (lead).

## Ethics Statement

The study adhered to the ethical standards outlined in the 1964 Declaration of Helsinki, along with subsequent amendments. The local ethics committee of Shefa Neuroscience Research Center, Tehran, Iran approved the experimental protocol. Informed consent was obtained from all individual participants included in the study.

## Consent

The authors have nothing to report.

## Conflicts of Interest

The authors declare no conflicts of interest.

## Supporting information


Data S1.
**Table S1**. Downregulated genes in epileptic temporal lobe tissue compared to autopsied tissue.
**Table S2**. Upregulated genes in epileptic temporal lobe tissue compared to autopsied tissue.
**Table S3**. Number of genes belonging to each module.
**Table S4**. Genes coexisting in the turquoise module (MeCp2 co‐expression genes), brown module (CDKL5 co‐expression genes) and pink module (BDNF co‐expression genes) groups, along with differentially expressed genes (DEGs).
**Table S5**. The data of the topological analysis using CytoHubba on the protein–protein interaction network.
**Figure S1**. Box plots illustrating gene expression data. The horizontal axis denotes the sample, while the vertical axis indicates the gene expression values.
**Figure S2**. Gene ontology (GO) biological process enrichment analysis of co‐expression genes and differentially expressed genes (DEGs) involving MeCp2, CDKL5 and BDNF. The network shows groups of terms or pathways associated with these genes.
**Figure S3**. Expression values of the MeCp2, CDKL5 and BDNF genes in epileptic and autoptic control hippocampus. Bar diagrams depicting the relative fold change (RFC) in the relative expression level of the MeCp2, CDKL5 and BDNF genes at mRNA levels. Bars represents the mean ± SEM. ns indicate not significant (*n* = 20–25).

## Data Availability

The datasets generated during and/or analyzed during the current study are available from the corresponding author upon reasonable request.
