# Supplementary material for: Unveiling Molecular Dynamics of MeCp2, CDKL5 and BDNF in the Hippocampus of Individuals With Intractable Mesial Temporal Lobe Epilepsy
Source: J Cell Mol Med. 2025 Jan 31;29(3):e70373. doi: 10.1111/jcmm.70373 (PMC11783159; doi:10.1111/jcmm.70373)

***Legends of supplementary figures and tables***

**Table** **Suppl. 1.** Downregulated genes in epileptic temporal lobe tissue compared to autopsied tissue.

**Table** **Suppl. 2:** Upregulated genes in epileptic temporal lobe tissue compared to autopsied tissue.

**Table Suppl. 3**: Number of genes belonging to each module.

**Table Suppl. 4.** Genes coexisting in the turquoise module (MeCp2 co-expression genes), brown module (CDKL5 co-expression genes), and pink module (BDNF co-expression genes) groups, along with differentially expressed genes (DEGs).

**Table** **Suppl. 5.** The data of the topological analysis using CytoHubba on the protein-protein interaction network.

**Figure** **Suppl. 1.** Box plots illustrating gene expression data. The horizontal axis denotes the sample, while the vertical axis indicates the gene expression values.

**Figure Suppl. 2.** Gene ontology (GO) biological process enrichment analysis of co-expression genes and differentially expressed genes (DEGs) involving MeCp2, CDKL5, and BDNF. The network shows groups of terms or pathways associated with these genes.

**Figure Suppl. 3.** Expression values of the MeCp2, CDKL5, and BDNF genes in epileptic and autoptic control hippocampus. Bar diagrams depicting the relative fold change (RFC) in the relative expression level of the MeCp2, CDKL5, and BDNF genes at mRNA levels. Bars represents the mean ± SEM. ns indicate not significant.

**S-Table 1.**

| Gene ID | Adjusted P-Value | P-Value | log2 Fold Change | Gene Symbol | Description |
| --- | --- | --- | --- | --- | --- |
| 22999 | 3.52E-06 | 6.26E-09 | -0.62199 | RIMS1 | regulating synaptic membrane exocytosis 1 |
| 11069 | 3.53E-06 | 6.50E-09 | -0.99609 | RAPGEF4 | Rap guanine nucleotide exchange factor 4 |
| 58476 | 5.22E-06 | 1.05E-08 | -1.2517 | TP53INP2 | tumor protein p53 inducible nuclear protein 2 |
| 4155 | 5.10E-05 | 1.40E-07 | -1.60295 | MBP | myelin basic protein |
| 400961 | 9.13E-05 | 2.79E-07 | -1.36397 | PAIP2B | poly(A) binding protein interacting protein 2B |
| 4539 | 0.000146 | 4.76E-07 | -1.03713 | ND4L | NADH dehydrogenase subunit 4L |
| 79957 | 0.00017 | 5.64E-07 | -0.90552 | PAQR6 | progestin and adipoQ receptor family member 6 |
| 8502 | 0.000584 | 2.67E-06 | -0.70201 | PKP4 | plakophilin 4 |
| 1.08E+08 | 0.000899 | 4.74E-06 | -0.84188 | LOC107985902 | replaced by ID 1496 |
| 4512 | 0.000899 | 4.72E-06 | -0.98877 | COX1 | cytochrome c oxidase subunit I |
| 4519 | 0.000939 | 5.00E-06 | -0.97367 | CYTB | cytochrome b |
| 3336 | 0.000973 | 5.33E-06 | -0.9404 | HSPE1 | heat shock protein family E (Hsp10) member 1 |
| 1.03E+08 | 0.00115 | 6.64E-06 | -1.20032 | LINC02552 | long intergenic non-protein coding RNA 2552 |
| 5053 | 0.00152 | 9.73E-06 | -0.74698 | PAH | phenylalanine hydroxylase |
| 83259 | 0.00161 | 1.09E-05 | -1.53305 | PCDH11Y | protocadherin 11 Y-linked |
| 54492 | 0.00164 | 1.12E-05 | -0.79092 | NEURL1B | neuralized E3 ubiquitin protein ligase 1B |
| 255631 | 0.00167 | 1.16E-05 | -0.61983 | COL24A1 | collagen type XXIV alpha 1 chain |
| 4538 | 0.00283 | 2.14E-05 | -0.89122 | ND4 | NADH dehydrogenase subunit 4 |
| 353189 | 0.003 | 2.34E-05 | -0.8063 | SLCO4C1 | solute carrier organic anion transporter family member 4C1 |
| 4725 | 0.00301 | 2.36E-05 | -0.50526 | NDUFS5 | NADH:ubiquinone oxidoreductase subunit S5 |
| 6128 | 0.00309 | 2.44E-05 | -0.51256 | RPL6 | ribosomal protein L6 |
| 4570 | 0.00374 | 3.21E-05 | -1.09301 | TRNN | tRNA-Asn |
| 8537 | 0.00386 | 3.42E-05 | -1.3524 | BCAS1 | brain enriched myelin associated protein 1 |
| 4303 | 0.00386 | 3.44E-05 | -0.5881 | FOXO4 | forkhead box O4 |
| 4550 | 0.00388 | 3.51E-05 | -1.02591 | RNR2 | l-rRNA |
| 4513 | 0.00388 | 3.50E-05 | -0.64293 | COX2 | cytochrome c oxidase subunit II |
| 222166 | 0.00475 | 4.43E-05 | -0.67189 | MTURN | maturin, neural progenitor differentiation regulator homolog |
| 1E+08 | 0.00522 | 4.95E-05 | -0.74507 | ZEB2-AS1 | ZEB2 antisense RNA 1 |
| 1.01E+08 | 0.00544 | 5.21E-05 | -0.65637 | MSANTD3-TMEFF1 | MSANTD3-TMEFF1 readthrough |
| 6152 | 0.00634 | 6.28E-05 | -0.51985 | RPL24 | ribosomal protein L24 |
| 1.05E+08 | 0.00641 | 6.38E-05 | -0.85158 | LOC105376121 | uncharacterized LOC105376121 |
| 440279 | 0.00661 | 6.72E-05 | -0.57883 | UNC13C | unc-13 homolog C |
| 8707 | 0.00664 | 6.89E-05 | -0.80144 | B3GALT2 | beta-1,3-galactosyltransferase 2 |
| 56892 | 0.00664 | 6.83E-05 | -0.92505 | TCIM | transcriptional and immune response regulator |
| 8577 | 0.00715 | 7.61E-05 | -0.67851 | TMEFF1 | transmembrane protein with EGF like and two follistatin like domains 1 |
| 81848 | 0.00723 | 7.73E-05 | -0.82352 | SPRY4 | sprouty RTK signaling antagonist 4 |
| 6160 | 0.00748 | 8.04E-05 | -0.60456 | RPL31 | ribosomal protein L31 |
| 6173 | 0.00823 | 9.10E-05 | -0.57548 | RPL36A | ribosomal protein L36a |
| 521 | 0.00833 | 9.26E-05 | -0.65313 | ATP5ME | ATP synthase membrane subunit e |
| 4536 | 0.00938 | 0.000105 | -0.81743 | ND2 | NADH dehydrogenase subunit 2 |
| 1.05E+08 | 0.0108 | 0.000127 | -1.06678 | LOC105375716 | uncharacterized LOC105375716 |
| 441951 | 0.0112 | 0.000134 | -0.72117 | ZFAS1 | ZNFX1 antisense RNA 1 |
| 3303 | 0.013 | 0.000161 | -1.01861 | HSPA1A | heat shock protein family A (Hsp70) member 1A |
| 94160 | 0.0148 | 0.000194 | -0.94604 | ABCC12 | ATP binding cassette subfamily C member 12 |
| 22829 | 0.0152 | 0.000202 | -2.44509 | NLGN4Y | neuroligin 4 Y-linked |
| 79622 | 0.0159 | 0.000215 | -0.82185 | SNRNP25 | small nuclear ribonucleoprotein U11/U12 subunit 25 |
| 123803 | 0.016 | 0.000217 | -0.62131 | NTAN1 | N-terminal asparagine amidase |
| 6201 | 0.016 | 0.00022 | -0.64918 | RPS7 | ribosomal protein S7 |
| 254778 | 0.0174 | 0.000248 | -0.71363 | VXN | vexin |
| 27087 | 0.0179 | 0.000258 | -0.56551 | B3GAT1 | beta-1,3-glucuronyltransferase 1 |
| 6206 | 0.0179 | 0.000261 | -0.69749 | RPS12 | ribosomal protein S12 |
| 54769 | 0.018 | 0.000265 | -0.82364 | DIRAS2 | DIRAS family GTPase 2 |
| 85397 | 0.0191 | 0.000285 | -0.83888 | RGS8 | regulator of G protein signaling 8 |
| 54543 | 0.0204 | 0.000314 | -0.73936 | TOMM7 | translocase of outer mitochondrial membrane 7 |
| 1846 | 0.0207 | 0.000322 | -0.77504 | DUSP4 | dual specificity phosphatase 4 |
| 5188 | 0.021 | 0.000328 | -0.57592 | GATB | glutamyl-tRNA amidotransferase subunit B |
| 1.02E+08 | 0.0219 | 0.000346 | -0.74275 | LOC101929473 | uncharacterized LOC101929473 |
| 65992 | 0.0219 | 0.000348 | -0.70765 | DDRGK1 | DDRGK domain containing 1 |
| 4540 | 0.0221 | 0.000352 | -0.64653 | ND5 | NADH dehydrogenase subunit 5 |
| 1.05E+08 | 0.0229 | 0.000369 | -1.1198 | LOC105378751 | uncharacterized LOC105378751 |
| 4336 | 0.0235 | 0.000382 | -1.72963 | MOBP | myelin associated oligodendrocyte basic protein |
| 2788 | 0.0236 | 0.000384 | -0.53345 | GNG7 | G protein subunit gamma 7 |
| 375057 | 0.0237 | 0.000396 | -0.63326 | STUM | stum, mechanosensory transduction mediator homolog |
| 1.02E+08 | 0.0237 | 0.000394 | -0.98051 | LOC101928491 | uncharacterized LOC101928491 |
| 7295 | 0.0237 | 0.000392 | -0.59745 | TXN | thioredoxin |
| 6129 | 0.0239 | 0.000401 | -0.53541 | RPL7 | ribosomal protein L7 |
| 539 | 0.0241 | 0.000406 | -0.57547 | ATP5PO | ATP synthase peripheral stalk subunit OSCP |
| 11224 | 0.0243 | 0.000414 | -0.74623 | RPL35 | ribosomal protein L35 |
| 9377 | 0.0267 | 0.000463 | -0.63038 | COX5A | cytochrome c oxidase subunit 5A |
| 219578 | 0.0267 | 0.000466 | -0.73792 | ZNF804B | zinc finger protein 804B |
| 1.01E+08 | 0.0285 | 0.000504 | -0.54311 | DLGAP1-AS4 | DLGAP1 antisense RNA 4 |
| 51019 | 0.0304 | 0.000546 | -0.54616 | WASHC3 | WASH complex subunit 3 |
| 1.08E+08 | 0.0324 | 0.000597 | -0.50367 | LOC107984658 | uncharacterized LOC107984658 |
| 4694 | 0.0324 | 0.000601 | -0.70081 | NDUFA1 | NADH:ubiquinone oxidoreductase subunit A1 |
| 22844 | 0.0336 | 0.000626 | -0.61208 | FRMPD1 | FERM and PDZ domain containing 1 |
| 2987 | 0.0344 | 0.000648 | -1.00156 | GUK1 | guanylate kinase 1 |
| 4535 | 0.0347 | 0.000655 | -0.65716 | ND1 | NADH dehydrogenase subunit 1 |
| 2495 | 0.0358 | 0.000685 | -0.61333 | FTH1 | ferritin heavy chain 1 |
| 29937 | 0.0379 | 0.000738 | -0.75587 | NENF | neudesin neurotrophic factor |
| 28996 | 0.0392 | 0.000772 | -0.68387 | HIPK2 | homeodomain interacting protein kinase 2 |
| 1592 | 0.0397 | 0.000783 | -1.13214 | CYP26A1 | cytochrome P450 family 26 subfamily A member 1 |
| 643246 | 0.0398 | 0.00079 | -0.64068 | MAP1LC3B2 | microtubule associated protein 1 light chain 3 beta 2 |
| 79658 | 0.04 | 0.000798 | -0.72658 | ARHGAP10 | Rho GTPase activating protein 10 |
| 1329 | 0.0412 | 0.000835 | -0.58032 | COX5B | cytochrome c oxidase subunit 5B |
| 7432 | 0.0414 | 0.000845 | -0.83958 | VIP | vasoactive intestinal peptide |
| 6230 | 0.0414 | 0.000848 | -0.60993 | RPS25 | ribosomal protein S25 |
| 1.05E+08 | 0.0444 | 0.00093 | -0.91826 | LOC105373899 | uncharacterized LOC105373899 |
| 389073 | 0.0474 | 0.00103 | -0.61724 | C2orf80 | chromosome 2 open reading frame 80 |
| 7439 | 0.0486 | 0.00106 | -0.55437 | BEST1 | bestrophin 1 |
| 1.05E+08 | 3.11E-17 | 1.64E-21 | 4.973657 | LOC105376380 | uncharacterized LOC105376380 |

**S-Table 2.**

| Gene ID | Adjusted P-Value | P-Value | log2 Fold Change | Gene Symbol | Description |
| --- | --- | --- | --- | --- | --- |
| 1.05E+08 | 3.11E-17 | 1.64E-21 | 4.973657 | LOC105376380 | uncharacterized LOC105376380 |
| 79875 | 1.15E-12 | 1.21E-16 | 1.767395 | THSD4 | thrombospondin type 1 domain containing 4 |
| 1.05E+08 | 3.54E-12 | 5.60E-16 | 1.797915 | SLC2A9-AS1 | SLC2A9 antisense RNA 1 |
| 5054 | 1.71E-11 | 3.60E-15 | 3.202406 | SERPINE1 | serpin family E member 1 |
| 1.12E+08 | 7.16E-11 | 1.89E-14 | 3.59324 | LOC112267956 |  |
| 1.05E+08 | 8.46E-11 | 2.67E-14 | 1.940434 | LOC105377016 | uncharacterized LOC105377016 |
| 55244 | 1.48E-10 | 5.44E-14 | 2.168319 | SLC47A1 | solute carrier family 47 member 1 |
| 2289 | 1.49E-10 | 6.29E-14 | 2.265581 | FKBP5 | FKBP prolyl isomerase 5 |
| 1832 | 3.52E-10 | 1.67E-13 | 2.452949 | DSP | desmoplakin |
| 1601 | 6.58E-10 | 3.47E-13 | 1.050077 | DAB2 | DAB adaptor protein 2 |
| 1278 | 1.36E-09 | 7.87E-13 | 2.448815 | COL1A2 | collagen type I alpha 2 chain |
| 4969 | 8.06E-09 | 5.09E-12 | 2.501316 | OGN | osteoglycin |
| 387700 | 9.86E-09 | 6.75E-12 | 3.171445 | SLC16A12 | solute carrier family 16 member 12 |
| 155038 | 2.58E-08 | 1.90E-11 | 1.016421 | GIMAP8 | GTPase, IMAP family member 8 |
| 1.05E+08 | 3.31E-08 | 2.61E-11 | 1.478891 | SCPPPQ1 | secretory calcium-binding phosphoprotein proline-glutamine rich 1 |
| 730 | 5.24E-08 | 4.42E-11 | 2.260318 | C7 | complement C7 |
| 7097 | 6.18E-08 | 5.53E-11 | 1.124049 | TLR2 | toll like receptor 2 |
| 81035 | 7.76E-08 | 7.35E-11 | 1.271473 | COLEC12 | collectin subfamily member 12 |
| 10276 | 1.03E-07 | 1.03E-10 | 1.248304 | NET1 | neuroepithelial cell transforming 1 |
| 3791 | 1.26E-07 | 1.33E-10 | 1.60981 | KDR | kinase insert domain receptor |
| 9332 | 1.44E-07 | 1.59E-10 | 2.083884 | CD163 | CD163 molecule |
| 4792 | 1.49E-07 | 1.72E-10 | 1.208864 | NFKBIA | NFKB inhibitor alpha |
| 54796 | 1.62E-07 | 1.96E-10 | 2.038505 | BNC2 | basonuclin 2 |
| 1.08E+08 | 5.24E-07 | 6.62E-10 | 2.23418 | LOC107984770 | uncharacterized LOC107984770 |
| 54716 | 6.17E-07 | 8.12E-10 | 2.349553 | SLC6A20 | solute carrier family 6 member 20 |
| 1.01E+08 | 8.26E-07 | 1.19E-09 | 3.911273 | MIR5690 | microRNA 5690 |
| 1.05E+08 | 8.26E-07 | 1.22E-09 | 1.9982 | LOC105375532 | uncharacterized LOC105375532 |
| 3698 | 8.26E-07 | 1.18E-09 | 1.743765 | ITIH2 | inter-alpha-trypsin inhibitor heavy chain 2 |
| 1908 | 9.18E-07 | 1.40E-09 | 1.31908 | EDN3 | endothelin 3 |
| 1293 | 1.06E-06 | 1.68E-09 | 2.354551 | COL6A3 | collagen type VI alpha 3 chain |
| 1.05E+08 | 1.82E-06 | 2.96E-09 | 2.444153 | LOC105372270 | uncharacterized LOC105372270 |
| 688 | 1.96E-06 | 3.30E-09 | 1.341609 | KLF5 | KLF transcription factor 5 |
| 1836 | 3.52E-06 | 6.30E-09 | 1.790874 | SLC26A2 | solute carrier family 26 member 2 |
| 302 | 3.84E-06 | 7.27E-09 | 1.435964 | ANXA2 | annexin A2 |
| 4323 | 4.07E-06 | 7.93E-09 | 1.087327 | MMP14 | matrix metallopeptidase 14 |
| 654 | 5.23E-06 | 1.07E-08 | 1.216732 | BMP6 | bone morphogenetic protein 6 |
| 2683 | 5.94E-06 | 1.25E-08 | 1.385501 | B4GALT1 | beta-1,4-galactosyltransferase 1 |
| 4360 | 6.13E-06 | 1.32E-08 | 1.540122 | MRC1 | mannose receptor C-type 1 |
| 26266 | 1.08E-05 | 2.39E-08 | 2.847446 | SLC13A4 | solute carrier family 13 member 4 |
| 3053 | 1.10E-05 | 2.49E-08 | 1.689387 | SERPIND1 | serpin family D member 1 |
| 55304 | 1.17E-05 | 2.71E-08 | 1.735413 | SPTLC3 | serine palmitoyltransferase long chain base subunit 3 |
| 10346 | 1.31E-05 | 3.11E-08 | 0.888354 | TRIM22 | tripartite motif containing 22 |
| 57194 | 1.84E-05 | 4.47E-08 | 1.205795 | ATP10A | ATPase phospholipid transporting 10A (putative) |
| 84913 | 2.05E-05 | 5.11E-08 | 1.623044 | ATOH8 | atonal bHLH transcription factor 8 |
| 407975 | 2.05E-05 | 5.18E-08 | 1.411938 | MIR17HG | miR-17-92a-1 cluster host gene |
| 6549 | 2.98E-05 | 7.70E-08 | 1.073367 | SLC9A2 | solute carrier family 9 member A2 |
| 27181 | 3.98E-05 | 1.05E-07 | 1.753674 | SIGLEC8 | sialic acid binding Ig like lectin 8 |
| 1.05E+08 | 4.31E-05 | 1.16E-07 | 1.510405 | LOC105374936 | uncharacterized LOC105374936 |
| 114907 | 5.21E-05 | 1.46E-07 | 0.602214 | FBXO32 | F-box protein 32 |
| 6423 | 7.84E-05 | 2.23E-07 | 1.114245 | SFRP2 | secreted frizzled related protein 2 |
| 8436 | 8.66E-05 | 2.54E-07 | 1.769805 | CAVIN2 | caveolae associated protein 2 |
| 169611 | 8.66E-05 | 2.55E-07 | 1.372803 | OLFML2A | olfactomedin like 2A |
| 1.02E+08 | 9.09E-05 | 2.73E-07 | 1.578827 | LOC101927069 | uncharacterized LOC101927069 |
| 716 | 9.89E-05 | 3.13E-07 | 0.885347 | C1S | complement C1s |
| 51279 | 9.89E-05 | 3.10E-07 | 1.099074 | C1RL | complement C1r subcomponent like |
| 1.02E+08 | 0.000109 | 3.51E-07 | 1.16995 | LOC101927516 | uncharacterized LOC101927516 |
| 653 | 0.000187 | 6.31E-07 | 2.959378 | BMP5 | bone morphogenetic protein 5 |
| 7048 | 0.000198 | 6.86E-07 | 1.066805 | TGFBR2 | transforming growth factor beta receptor 2 |
| 3134 | 0.000198 | 6.88E-07 | 0.906096 | HLA-F | major histocompatibility complex, class I, F |
| 1.05E+08 | 0.000203 | 7.16E-07 | 1.945113 | LOC105377979 | uncharacterized LOC105377979 |
| 286133 | 0.000213 | 7.62E-07 | 1.745633 | SCARA5 | scavenger receptor class A member 5 |
| 7832 | 0.000242 | 8.79E-07 | 1.370328 | BTG2 | BTG anti-proliferation factor 2 |
| 89857 | 0.000255 | 9.54E-07 | 1.55193 | KLHL6 | kelch like family member 6 |
| 93166 | 0.000255 | 9.46E-07 | 1.62035 | PRDM6 | PR/SET domain 6 |
| 7100 | 0.000322 | 1.24E-06 | 1.198923 | TLR5 | toll like receptor 5 |
| 187 | 0.000322 | 1.22E-06 | 1.653943 | APLNR | apelin receptor |
| 1.05E+08 | 0.000345 | 1.35E-06 | 1.719222 | LOC105374768 | uncharacterized LOC105374768 |
| 1.08E+08 | 0.00035 | 1.38E-06 | 1.265802 | LOC107985362 | uncharacterized LOC107985362 |
| 2335 | 0.000433 | 1.73E-06 | 0.91951 | FN1 | fibronectin 1 |
| 3488 | 0.000452 | 1.83E-06 | 0.890649 | IGFBP5 | insulin like growth factor binding protein 5 |
| 3059 | 0.00047 | 1.96E-06 | 1.230943 | HCLS1 | hematopoietic cell-specific Lyn substrate 1 |
| 6916 | 0.00047 | 1.94E-06 | 1.395843 | TBXAS1 | thromboxane A synthase 1 |
| 5239 | 0.000485 | 2.05E-06 | 0.619202 | PGM5 | phosphoglucomutase 5 |
| 723961 | 0.000485 | 2.07E-06 | 1.621742 | INS-IGF2 | INS-IGF2 readthrough |
| 316 | 0.000506 | 2.18E-06 | 1.322778 | AOX1 | aldehyde oxidase 1 |
| 8828 | 0.000512 | 2.24E-06 | 0.611458 | NRP2 | neuropilin 2 |
| 26509 | 0.000578 | 2.56E-06 | 0.833392 | MYOF | myoferlin |
| 1.02E+08 | 0.000584 | 2.66E-06 | 1.146689 | KLF9-DT | KLF9 divergent transcript |
| 9152 | 0.000584 | 2.68E-06 | 1.894169 | SLC6A5 | solute carrier family 6 member 5 |
| 5583 | 0.000586 | 2.71E-06 | 0.943925 | PRKCH | protein kinase C eta |
| 400823 | 0.000626 | 2.95E-06 | 1.525233 | FAM177B | family with sequence similarity 177 member B |
| 6540 | 0.00066 | 3.16E-06 | 1.550901 | SLC6A13 | solute carrier family 6 member 13 |
| 5173 | 0.000669 | 3.24E-06 | 2.335244 | PDYN | prodynorphin |
| 1281 | 0.000675 | 3.33E-06 | 1.936527 | COL3A1 | collagen type III alpha 1 chain |
| 3481 | 0.000675 | 3.34E-06 | 1.606612 | IGF2 | insulin like growth factor 2 |
| 4958 | 0.000782 | 3.92E-06 | 1.358499 | OMD | osteomodulin |
| 79056 | 0.000782 | 3.95E-06 | 1.637067 | PRRG4 | proline rich and Gla domain 4 |
| 4599 | 0.000892 | 4.56E-06 | 0.703676 | MX1 | MX dynamin like GTPase 1 |
| 5272 | 0.000893 | 4.61E-06 | 0.656946 | SERPINB9 | serpin family B member 9 |
| 283521 | 0.000951 | 5.11E-06 | 1.589246 | TMEM272 | transmembrane protein 272 |
| 80760 | 0.000973 | 5.33E-06 | 0.827112 | ITIH5 | inter-alpha-trypsin inhibitor heavy chain 5 |
| 3111 | 0.000981 | 5.43E-06 | 1.400956 | HLA-DOA | major histocompatibility complex, class II, DO alpha |
| 10144 | 0.001 | 5.58E-06 | 0.512473 | FAM13A | family with sequence similarity 13 member A |
| 130399 | 0.00102 | 5.77E-06 | 1.1775 | ACVR1C | activin A receptor type 1C |
| 57091 | 0.00103 | 5.88E-06 | 1.199187 | CASS4 | Cas scaffold protein family member 4 |
| 6039 | 0.00115 | 6.59E-06 | 1.522771 | RNASE6 | ribonuclease A family member k6 |
| 80008 | 0.0012 | 7.13E-06 | 1.547844 | TMEM156 | transmembrane protein 156 |
| 255743 | 0.0012 | 7.09E-06 | 0.954842 | NPNT | nephronectin |
| 56245 | 0.0012 | 7.04E-06 | 1.092762 | C21orf62 | chromosome 21 open reading frame 62 |
| 10365 | 0.00126 | 7.68E-06 | 1.194073 | KLF2 | KLF transcription factor 2 |
| 861 | 0.00126 | 7.70E-06 | 1.023806 | RUNX1 | RUNX family transcription factor 1 |
| 11326 | 0.00126 | 7.60E-06 | 1.72939 | VSIG4 | V-set and immunoglobulin domain containing 4 |
| 3106 | 0.00128 | 7.95E-06 | 1.12604 | HLA-B | major histocompatibility complex, class I, B |
| 57214 | 0.00128 | 7.96E-06 | 1.079273 | CEMIP | cell migration inducing hyaluronidase 1 |
| 25959 | 0.00134 | 8.39E-06 | 0.798023 | KANK2 | KN motif and ankyrin repeat domains 2 |
| 715 | 0.00134 | 8.48E-06 | 0.896298 | C1R | complement C1r |
| 6886 | 0.00152 | 9.82E-06 | 1.266317 | TAL1 | TAL bHLH transcription factor 1, erythroid differentiation factor |
| 90 | 0.00152 | 9.79E-06 | 0.625138 | ACVR1 | activin A receptor type 1 |
| 2354 | 0.00153 | 9.98E-06 | 1.735533 | FOSB | FosB proto-oncogene, AP-1 transcription factor subunit |
| 10475 | 0.00161 | 1.08E-05 | 0.991848 | TRIM38 | tripartite motif containing 38 |
| 23533 | 0.00161 | 1.08E-05 | 1.456586 | PIK3R5 | phosphoinositide-3-kinase regulatory subunit 5 |
| 1829 | 0.00161 | 1.10E-05 | 1.763477 | DSG2 | desmoglein 2 |
| 9050 | 0.00161 | 1.09E-05 | 1.461359 | PSTPIP2 | proline-serine-threonine phosphatase interacting protein 2 |
| 2487 | 0.00167 | 1.15E-05 | 1.001554 | FRZB | frizzled related protein |
| 9754 | 0.00167 | 1.17E-05 | 1.269728 | STARD8 | StAR related lipid transfer domain containing 8 |
| 119587 | 0.00168 | 1.19E-05 | 1.464242 | CPXM2 | carboxypeptidase X, M14 family member 2 |
| 2331 | 0.00187 | 1.35E-05 | 2.081073 | FMOD | fibromodulin |
| 93349 | 0.00187 | 1.34E-05 | 0.819505 | SP140L | SP140 nuclear body protein like |
| 474344 | 0.00187 | 1.34E-05 | 1.033383 | GIMAP6 | GTPase, IMAP family member 6 |
| 647174 | 0.00188 | 1.37E-05 | 0.620009 | SERPINE3 | serpin family E member 3 |
| 11213 | 0.00196 | 1.44E-05 | 1.139417 | IRAK3 | interleukin 1 receptor associated kinase 3 |
| 2210 | 0.00212 | 1.57E-05 | 1.173152 | FCGR1BP | Fc gamma receptor Ib, pseudogene |
| 2 | 0.00232 | 1.74E-05 | 0.79364 | A2M | alpha-2-macroglobulin |
| 22925 | 0.00261 | 1.97E-05 | 0.782682 | PLA2R1 | phospholipase A2 receptor 1 |
| 4609 | 0.0029 | 2.21E-05 | 1.123226 | MYC | MYC proto-oncogene, bHLH transcription factor |
| 960 | 0.00298 | 2.31E-05 | 1.399877 | CD44 | CD44 molecule (Indian blood group) |
| 3606 | 0.00311 | 2.47E-05 | 1.12202 | IL18 | interleukin 18 |
| 1.05E+08 | 0.00324 | 2.61E-05 | 1.764347 | LOC105374020 | uncharacterized LOC105374020 |
| 90246 | 0.00324 | 2.62E-05 | 1.377342 | LOC90246 | uncharacterized LOC90246 |
| 11015 | 0.00324 | 2.63E-05 | 0.813071 | KDELR3 | KDEL endoplasmic reticulum protein retention receptor 3 |
| 3953 | 0.00329 | 2.71E-05 | 0.968386 | LEPR | leptin receptor |
| 151887 | 0.00329 | 2.72E-05 | 0.980932 | CCDC80 | coiled-coil domain containing 80 |
| 1.05E+08 | 0.00329 | 2.71E-05 | 1.009482 | LOC105376090 | uncharacterized LOC105376090 |
| 149563 | 0.00331 | 2.76E-05 | 1.238574 | SRARP | steroid receptor associated and regulated protein |
| 7133 | 0.00332 | 2.79E-05 | 1.174806 | TNFRSF1B | TNF receptor superfamily member 1B |
| 285512 | 0.00332 | 2.81E-05 | 0.643748 | FAM13A-AS1 | FAM13A antisense RNA 1 |
| 6542 | 0.00332 | 2.82E-05 | 1.141249 | SLC7A2 | solute carrier family 7 member 2 |
| 1490 | 0.0035 | 2.98E-05 | 0.923812 | CCN2 | cellular communication network factor 2 |
| 1906 | 0.00377 | 3.26E-05 | 1.228505 | EDN1 | endothelin 1 |
| 1.05E+08 | 0.00379 | 3.29E-05 | 1.302726 | LOC105377918 | uncharacterized LOC105377918 |
| 1.08E+08 | 0.00386 | 3.38E-05 | 1.734868 | LOC107986853 | uncharacterized LOC107986853 |
| 54541 | 0.00386 | 3.41E-05 | 0.994313 | DDIT4 | DNA damage inducible transcript 4 |
| 26524 | 0.00386 | 3.46E-05 | 0.896209 | LATS2 | large tumor suppressor kinase 2 |
| 3678 | 0.00396 | 3.60E-05 | 1.278856 | ITGA5 | integrin subunit alpha 5 |
| 7704 | 0.00411 | 3.77E-05 | 0.623725 | ZBTB16 | zinc finger and BTB domain containing 16 |
| 115111 | 0.00416 | 3.83E-05 | 2.147676 | SLC26A7 | solute carrier family 26 member 7 |
| 51267 | 0.00439 | 4.07E-05 | 1.317122 | CLEC1A | C-type lectin domain family 1 member A |
| 1.05E+08 | 0.00489 | 4.59E-05 | 1.805185 | LOC105372093 | uncharacterized LOC105372093 |
| 1.03E+08 | 0.00498 | 4.70E-05 | 1.174937 | PDE4DIPP7 | PDE4DIP pseudogene 7 |
| 1.02E+08 | 0.00531 | 5.06E-05 | 1.662011 | LOC101928875 | |
| 3007 | 0.0056 | 5.40E-05 | 1.286685 | H1-3 | H1.3 linker histone, cluster member |
| 91607 | 0.00578 | 5.60E-05 | 0.608411 | SLFN11 | schlafen family member 11 |
| 89790 | 0.006 | 5.84E-05 | 1.156056 | SIGLEC10 | sialic acid binding Ig like lectin 10 |
| 7545 | 0.00601 | 5.89E-05 | 1.222677 | ZIC1 | Zic family member 1 |
| 717 | 0.00609 | 6.00E-05 | 1.093578 | C2 | complement C2 |
| 1.02E+08 | 0.00642 | 6.43E-05 | 1.169254 | GATA2-AS1 | GATA2 antisense RNA 1 |
| 1842 | 0.00642 | 6.45E-05 | 1.108125 | ECM2 | extracellular matrix protein 2 |
| 720 | 0.00654 | 6.61E-05 | 1.181463 | C4A | complement C4A (Rodgers blood group) |
| 1545 | 0.00664 | 6.82E-05 | 0.87657 | CYP1B1 | cytochrome P450 family 1 subfamily B member 1 |
| 721 | 0.00664 | 6.90E-05 | 1.19714 | C4B | complement C4B (Chido blood group) |
| 6446 | 0.00664 | 6.96E-05 | 0.866087 | SGK1 | serum/glucocorticoid regulated kinase 1 |
| 80833 | 0.00664 | 6.95E-05 | 0.963398 | APOL3 | apolipoprotein L3 |
| 1E+08 | 0.00713 | 7.55E-05 | 1.440908 | FRG2C | FSHD region gene 2 family member C |
| 7465 | 0.00713 | 7.52E-05 | 0.891804 | WEE1 | WEE1 G2 checkpoint kinase |
| 6672 | 0.00761 | 8.21E-05 | 0.895536 | SP100 | SP100 nuclear antigen |
| 10184 | 0.00761 | 8.26E-05 | 0.603021 | LHFPL2 | LHFPL tetraspan subfamily member 2 |
| 140 | 0.00806 | 8.83E-05 | 1.456442 | ADORA3 | adenosine A3 receptor |
| 2740 | 0.00818 | 9.01E-05 | 1.286815 | GLP1R | glucagon like peptide 1 receptor |
| 200424 | 0.00905 | 0.000101 | 0.531234 | TET3 | tet methylcytosine dioxygenase 3 |
| 1075 | 0.00976 | 0.00011 | 0.708125 | CTSC | cathepsin C |
| 58538 | 0.0099 | 0.000112 | 1.184671 | MPP4 | MAGUK p55 scaffold protein 4 |
| 257106 | 0.00994 | 0.000114 | 0.881732 | ARHGAP30 | Rho GTPase activating protein 30 |
| 170690 | 0.00994 | 0.000114 | 0.982633 | ADAMTS16 | ADAM metallopeptidase with thrombospondin type 1 motif 16 |
| 8737 | 0.0103 | 0.000118 | 0.530217 | RIPK1 | receptor interacting serine/threonine kinase 1 |
| 343263 | 0.0105 | 0.000122 | 1.392826 | MYBPHL | myosin binding protein H like |
| 403340 | 0.0106 | 0.000123 | 1.423384 | MGC70870 | C-terminal binding protein 2 pseudogene |
| 1.03E+08 | 0.0107 | 0.000126 | 1.360862 | LIVAR | liver cell viability associated lncRNA |
| 7538 | 0.0107 | 0.000126 | 1.352528 | ZFP36 | ZFP36 ring finger protein |
| 1.08E+08 | 0.0108 | 0.000127 | 0.846169 | LOC107987150 | |
| 22915 | 0.0112 | 0.000135 | 1.264508 | MMRN1 | multimerin 1 |
| 1E+08 | 0.0112 | 0.000135 | 1.943193 | LOC100419170 | toll like receptor 2 pseudogene |
| 2296 | 0.0112 | 0.000133 | 1.216066 | FOXC1 | forkhead box C1 |
| 162073 | 0.0112 | 0.000135 | 0.509323 | ITPRIPL2 | ITPRIP like 2 |
| 55733 | 0.0113 | 0.000137 | 0.692907 | HHAT | hedgehog acyltransferase |
| 8854 | 0.0113 | 0.000138 | 0.958504 | ALDH1A2 | aldehyde dehydrogenase 1 family member A2 |
| 6480 | 0.0119 | 0.000146 | 0.669312 | ST6GAL1 | ST6 beta-galactoside alpha-2,6-sialyltransferase 1 |
| 57664 | 0.0119 | 0.000146 | 1.125795 | PLEKHA4 | pleckstrin homology domain containing A4 |
| 158257 | 0.0122 | 0.000151 | 0.847005 | LINC02603 | long intergenic non-protein coding RNA 2603 |
| 5146 | 0.0122 | 0.000151 | 0.939877 | PDE6C | phosphodiesterase 6C |
| 25890 | 0.0133 | 0.000167 | 0.668715 | ABI3BP | ABI family member 3 binding protein |
| 8857 | 0.0134 | 0.000169 | 1.417394 | FCGBP | Fc gamma binding protein |
| 54502 | 0.0141 | 0.000178 | 1.053729 | RBM47 | RNA binding motif protein 47 |
| 1.08E+08 | 0.0141 | 0.000179 | 0.904132 | LOC107984561 | uncharacterized LOC107984561 |
| 58475 | 0.0141 | 0.00018 | 1.250291 | MS4A7 | membrane spanning 4-domains A7 |
| 6997 | 0.0144 | 0.000184 | 1.265462 | TDGF1 | teratocarcinoma-derived growth factor 1 |
| 9792 | 0.0144 | 0.000186 | 0.667849 | SERTAD2 | SERTA domain containing 2 |
| 3115 | 0.0145 | 0.000187 | 1.084656 | HLA-DPB1 | major histocompatibility complex, class II, DP beta 1 |
| 1.02E+08 | 0.0145 | 0.000187 | 1.616143 | LOC101927627 | uncharacterized LOC101927627 |
| 3908 | 0.0145 | 0.000189 | 0.624508 | LAMA2 | laminin subunit alpha 2 |
| 3597 | 0.0145 | 0.000189 | 0.852235 | IL13RA1 | interleukin 13 receptor subunit alpha 1 |
| 133121 | 0.0145 | 0.00019 | 0.829574 | ENPP6 | ectonucleotide pyrophosphatase/phosphodiesterase 6 |
| 1.05E+08 | 0.015 | 0.000198 | 1.098723 | LOC105369309 | uncharacterized LOC105369309 |
| 90102 | 0.0153 | 0.000204 | 0.931242 | PHLDB2 | pleckstrin homology like domain family B member 2 |
| 6913 | 0.0154 | 0.000206 | 1.702954 | TBX15 | T-box transcription factor 15 |
| 29128 | 0.0159 | 0.000215 | 1.14287 | UHRF1 | ubiquitin like with PHD and ring finger domains 1 |
| 10894 | 0.016 | 0.000218 | 1.092746 | LYVE1 | lymphatic vessel endothelial hyaluronan receptor 1 |
| 944 | 0.016 | 0.000219 | 1.51309 | TNFSF8 | TNF superfamily member 8 |
| 7543 | 0.0161 | 0.000222 | 0.521181 | ZFX | zinc finger protein X-linked |
| 10320 | 0.0164 | 0.000228 | 1.052477 | IKZF1 | IKAROS family zinc finger 1 |
| 116159 | 0.0165 | 0.00023 | 0.685724 | CYYR1 | cysteine and tyrosine rich 1 |
| 7711 | 0.0172 | 0.000241 | 0.527042 | ZNF155 | zinc finger protein 155 |
| 7743 | 0.0172 | 0.000242 | 0.739258 | ZNF189 | zinc finger protein 189 |
| 639 | 0.0173 | 0.000244 | 0.712875 | PRDM1 | PR/SET domain 1 |
| 1.08E+08 | 0.0173 | 0.000246 | 1.387464 | LOC107987183 | |
| 2355 | 0.0174 | 0.000248 | 0.563326 | FOSL2 | FOS like 2, AP-1 transcription factor subunit |
| 284454 | 0.0174 | 0.000249 | 1.874237 | MIR23AHG | miR-23a/27a/24-2 cluster host gene |
| 11309 | 0.0179 | 0.000257 | 0.797403 | SLCO2B1 | solute carrier organic anion transporter family member 2B1 |
| 1.05E+08 | 0.018 | 0.000265 | 0.830308 | LOC105375689 | uncharacterized LOC105375689 |
| 4811 | 0.0186 | 0.000276 | 0.809297 | NID1 | nidogen 1 |
| 55971 | 0.0187 | 0.000278 | 1.069242 | BAIAP2L1 | BAR/IMD domain containing adaptor protein 2 like 1 |
| 6518 | 0.0191 | 0.000286 | 1.050332 | SLC2A5 | solute carrier family 2 member 5 |
| 1052 | 0.0192 | 0.000289 | 1.333685 | CEBPD | CCAAT enhancer binding protein delta |
| 1.08E+08 | 0.0192 | 0.000288 | 0.594989 | LOC107987245 | uncharacterized LOC107987245 |
| 920 | 0.0195 | 0.000295 | 0.93199 | CD4 | CD4 molecule |
| 1.01E+08 | 0.02 | 0.000305 | 0.855591 | GIMAP1-GIMAP5 | GIMAP1-GIMAP5 readthrough |
| 1992 | 0.0201 | 0.000308 | 0.626703 | SERPINB1 | serpin family B member 1 |
| 1.08E+08 | 0.0201 | 0.000308 | 0.91792 | LOC107984616 | |
| 5318 | 0.0202 | 0.000311 | 0.764315 | PKP2 | plakophilin 2 |
| 286464 | 0.0204 | 0.000316 | 0.941017 | CFAP47 | cilia and flagella associated protein 47 |
| 27151 | 0.0209 | 0.000326 | 1.341125 | CPAMD8 | C3 and PZP like alpha-2-macroglobulin domain containing 8 |
| 131405 | 0.0214 | 0.000338 | 0.66191 | TRIM71 | tripartite motif containing 71 |
| 2838 | 0.0219 | 0.000349 | 1.15972 | GPR15 | G protein-coupled receptor 15 |
| 6493 | 0.0223 | 0.000357 | 0.700782 | SIM2 | SIM bHLH transcription factor 2 |
| 54504 | 0.0234 | 0.000379 | 0.653683 | CPVL | carboxypeptidase vitellogenic like |
| 29842 | 0.0237 | 0.000393 | 1.607372 | TFCP2L1 | transcription factor CP2 like 1 |
| 64332 | 0.0237 | 0.000389 | 0.84199 | NFKBIZ | NFKB inhibitor zeta |
| 1.08E+08 | 0.0237 | 0.000392 | 1.10936 | LOC107986148 | replaced by ID 25976 |
| 91010 | 0.0237 | 0.000395 | 0.532978 | FMNL3 | formin like 3 |
| 201799 | 0.0241 | 0.000406 | 0.796952 | TMEM154 | transmembrane protein 154 |
| 1634 | 0.0243 | 0.000413 | 0.943854 | DCN | decorin |
| 9383 | 0.0247 | 0.000421 | 3.810405 | TSIX | TSIX transcript, XIST antisense RNA |
| 1.05E+08 | 0.0251 | 0.000429 | 0.773329 | LOC105374407 | |
| 148103 | 0.0253 | 0.000434 | 0.657486 | ZNF599 | zinc finger protein 599 |
| 64407 | 0.0256 | 0.000442 | 1.404323 | RGS18 | regulator of G protein signaling 18 |
| 6840 | 0.0256 | 0.000442 | 0.664006 | SVIL | supervillin |
| 1.02E+08 | 0.0256 | 0.000443 | 0.658981 | LOC101929185 | uncharacterized LOC101929185 |
| 719 | 0.0268 | 0.000468 | 1.1894 | C3AR1 | complement C3a receptor 1 |
| 3099 | 0.0271 | 0.000475 | 0.805071 | HK2 | hexokinase 2 |
| 1.05E+08 | 0.0277 | 0.000488 | 0.846377 | LOC105370525 | uncharacterized LOC105370525 |
| 3624 | 0.0282 | 0.000498 | 0.95889 | INHBA | inhibin subunit beta A |
| 1.05E+08 | 0.0285 | 0.000506 | 1.421638 | LOC105378575 | uncharacterized LOC105378575 |
| 1.05E+08 | 0.0292 | 0.000519 | 0.938122 | LOC105373581 | uncharacterized LOC105373581 |
| 1.08E+08 | 0.0298 | 0.000533 | 0.931697 | LOC107984079 | uncharacterized LOC107984079 |
| 283298 | 0.0303 | 0.000542 | 1.016053 | OLFML1 | olfactomedin like 1 |
| 1.05E+08 | 0.0308 | 0.000556 | 1.104153 | LINC01709 | long intergenic non-protein coding RNA 1709 |
| 8515 | 0.0312 | 0.000564 | 1.288192 | ITGA10 | integrin subunit alpha 10 |
| 3426 | 0.0317 | 0.000574 | 1.061999 | CFI | complement factor I |
| 8942 | 0.032 | 0.000585 | 1.159424 | KYNU | kynureninase |
| 1.08E+08 | 0.032 | 0.000582 | 0.981445 | LOC107987102 | uncharacterized LOC107987102 |
| 55340 | 0.0321 | 0.000588 | 0.892836 | GIMAP5 | GTPase, IMAP family member 5 |
| 54491 | 0.0324 | 0.000597 | 0.651613 | OTULINL | OTU deubiquitinase with linear linkage specificity like |
| 414152 | 0.0324 | 0.000601 | 1.347345 | C10orf105 | chromosome 10 open reading frame 105 |
| 4939 | 0.0337 | 0.000632 | 0.899571 | OAS2 | 2'-5'-oligoadenylate synthetase 2 |
| 25878 | 0.0337 | 0.000632 | 1.033568 | MXRA5 | matrix remodeling associated 5 |
| 1.05E+08 | 0.0347 | 0.000653 | 0.809509 | LOC105378281 | uncharacterized LOC105378281 |
| 54206 | 0.0352 | 0.000669 | 0.5202 | ERRFI1 | ERBB receptor feedback inhibitor 1 |
| 1.01E+08 | 0.0352 | 0.00067 | 1.041533 | TMEM30A-DT | TMEM30A divergent transcript |
| 4258 | 0.0356 | 0.000679 | 0.609845 | MGST2 | microsomal glutathione S-transferase 2 |
| 3133 | 0.0358 | 0.000687 | 0.649195 | HLA-E | major histocompatibility complex, class I, E |
| 5265 | 0.0364 | 0.000704 | 1.173938 | SERPINA1 | serpin family A member 1 |
| 7503 | 0.0365 | 0.000707 | 3.751184 | XIST | X inactive specific transcript |
| 127833 | 0.0384 | 0.00075 | 0.82287 | SYT2 | synaptotagmin 2 |
| 1326 | 0.039 | 0.000764 | 0.555623 | MAP3K8 | mitogen-activated protein kinase kinase kinase 8 |
| 8324 | 0.039 | 0.000767 | 0.806386 | FZD7 | frizzled class receptor 7 |
| 134265 | 0.0397 | 0.000785 | 0.849257 | AFAP1L1 | actin filament associated protein 1 like 1 |
| 92346 | 0.0398 | 0.000792 | 1.041009 | C1orf105 | chromosome 1 open reading frame 105 |
| 64231 | 0.0411 | 0.000824 | 1.342018 | MS4A6A | membrane spanning 4-domains A6A |
| 712 | 0.0412 | 0.000828 | 1.260301 | C1QA | complement C1q A chain |
| 54852 | 0.0412 | 0.000831 | 0.697775 | PAQR5 | progestin and adipoQ receptor family member 5 |
| 4481 | 0.0414 | 0.000845 | 1.020651 | MSR1 | macrophage scavenger receptor 1 |
| 6926 | 0.0414 | 0.000844 | 0.965765 | TBX3 | T-box transcription factor 3 |
| 2634 | 0.0414 | 0.000852 | 0.655595 | GBP2 | guanylate binding protein 2 |
| 57716 | 0.0415 | 0.000857 | 1.351305 | PRX | periaxin |
| 64581 | 0.0432 | 0.000899 | 1.225679 | CLEC7A | C-type lectin domain containing 7A |
| 8989 | 0.0433 | 0.000903 | 0.766883 | TRPA1 | transient receptor potential cation channel subfamily A member 1 |
| 1.01E+08 | 0.0435 | 0.000909 | 0.992366 | IGSF11-AS1 | IGSF11 antisense RNA 1 |
| 5730 | 0.0444 | 0.000934 | 0.838282 | PTGDS | prostaglandin D2 synthase |
| 55534 | 0.0449 | 0.000948 | 0.506069 | MAML3 | mastermind like transcriptional coactivator 3 |
| 1.02E+08 | 0.0449 | 0.000949 | 1.032436 | ZNF451-AS1 | ZNF451 regulatory antisense RNA 1 |
| 55303 | 0.0452 | 0.000957 | 0.729986 | GIMAP4 | GTPase, IMAP family member 4 |
| 5514 | 0.0459 | 0.000976 | 0.57642 | PPP1R10 | protein phosphatase 1 regulatory subunit 10 |
| 199920 | 0.046 | 0.000982 | 0.785228 | FYB2 | FYN binding protein 2 |
| 8832 | 0.0468 | 0.001 | 1.029182 | CD84 | CD84 molecule |
| 1.01E+08 | 0.0472 | 0.00101 | 0.891441 | LOC100996318 | uncharacterized LOC100996318 |
| 10797 | 0.0474 | 0.00102 | 0.755204 | MTHFD2 | methylenetetrahydrofolate dehydrogenase (NADP+ dependent) 2, methenyltetrahydrofolate cyclohydrolase |
| 1.05E+08 | 0.0474 | 0.00103 | 0.709847 | CHMP3-AS1 | CHMP3 and RNF103 antisense RNA 1 |
| 3575 | 0.0474 | 0.00103 | 1.234131 | IL7R | interleukin 7 receptor |
| 1.12E+08 | 0.0474 | 0.00103 | 0.831758 | LOC112268061 | uncharacterized LOC112268061 |
| 2857 | 0.0496 | 0.00109 | 1.070187 | GPR34 | G protein-coupled receptor 34 |

**S-Table 3.**

| Module color | Number of the genes |
| --- | --- |
| black | 508 |
| blue | 2445 |
| brown | 2431 |
| cyan | 59 |
| green | 898 |
| greenyellow | 129 |
| grey | 24 |
| magenta | 243 |
| midnightblue | 50 |
| pink | 245 |
| purple | 168 |
| red | 835 |
| salmon | 78 |
| tan | 101 |
| turquoise | 2757 |
| yellow | 1976 |

**S-Table 4.**

| **Names** | **total** | **Elements** |
| --- | --- | --- |
| **DEGs *Turquoise*** | 24 | LHFPL2 MOBP SLFN11 ITIH5 RUNX1 HSPA1A PPP1R10 PCDH11Y TRIM71 SP140L TET3 B3GAT1 AFAP1L1 MXRA5 ZNF189 SYT2 NRP2 EDN3 SP100 IGF2 FMNL3 BCAS1 NLGN4Y APOL3 |
| **DEGs *Brown*** | 48 | CPXM2 TMEM156 ATP10A OLFML2A IL18 SLC9A2 PKP2 STARD8 OGN GIMAP4 TAL1 ST6GAL1 RPL31 SLC47A1 ACVR1C SLC6A20 SERPINB1 GPR34 MMRN1 THSD4 C1RL PIK3R5 SERPINB9 ATOH8 ZFP36 IL13RA1 NET1 MRC1 RGS18 COLEC12 B3GALT2 MX1 PRKCH RPS25 MAP1LC3B2 CYP1B1 IKZF1 C3AR1 BEST1 KYNU SLC6A13 MTURN HK2 RIPK1 ITIH2 GATB MYBPHL CTSC |
| **DEGs**  ***Pink*** | 4 | MS4A6A DDIT4 ERRFI1 CFI |
| **DEGs** | 312 | CD44 ITGA5 BTG2 EDN1 SLC2A5 SIGLEC8 A2M LOC100419170 SLCO2B1 LOC105373899 DUSP4 SERPINE3 LOC101927516 SLC13A4 LOC100996318 TRNN LOC107984079 LOC107987150 WASHC3 LOC105374020 SFRP2 KLHL6 NENF LOC101928875 OMD SVIL CPVL ABCC12 KLF2 LOC107985902 HLA-DOA DLGAP1-AS4 MIR17HG ATP5PO TLR5 FKBP5 ZFAS1 NPNT GIMAP1-GIMAP5 GIMAP6 CAVIN2 MYC SPTLC3 ARHGAP10 FAM13A ITGA10 ANXA2 C4B CFAP47 LOC105378751 MMP14 LOC90246 OAS2 C2orf80 KLF5 TNFSF8 LIVAR LOC105378281 VIP TBX3 PRX SLC26A2 C1orf105 GNG7 CD84 MBP SIM2 FRMPD1 PGM5 TMEM272 C10orf105 FOSB NEURL1B COL24A1 NFKBIA SCPPPQ1 C1QA DCN LOC105377016 GATA2-AS1 BNC2 SLC26A7 AOX1 COL1A2 ND5 BAIAP2L1 CASS4 COX5A LAMA2 NID1 ND1 CD4 DSP TCIM LOC107984616 FAM13A-AS1 LOC105378575 PAQR6 FCGR1BP TMEM30A-DT OTULINL B4GALT1 LINC02603 PRRG4 TP53INP2 SERPINA1 ALDH1A2 GLP1R LEPR H1-3 LOC105374407 INS-IGF2 LINC02552 FZD7 MAP3K8 ZBTB16 MAML3 PKP4 ND4 GBP2 ABI3BP RPL6 C7 KDELR3 SERPIND1 MSANTD3-TMEFF1 LINC01709 TMEM154 PSTPIP2 FRG2C SLC7A2 COL3A1 CD163 FOSL2 ZNF155 LATS2 SRARP PTGDS FOXO4 SLC6A5 FAM177B LOC101929185 ZNF451-AS1 CCDC80 RPS12 LOC107987102 C21orf62 TNFRSF1B CLEC1A PAQR5 TRIM38 LOC107987183 TDGF1 COX5B IL7R LOC107985362 TGFBR2 HLA-B HIPK2 ENPP6 FRZB COL6A3 TMEFF1 PLEKHA4 GIMAP5 ND4L LOC105369309 UHRF1 LOC107987245 TOMM7 FBXO32 LOC105376090 STUM PDYN CYP26A1 FYB2 LOC105372270 CYTB RPL35 KLF9-DT PDE6C LOC105377979 MPP4 ADAMTS16 NTAN1 FCGBP PLA2R1 ITPRIPL2 CPAMD8 FN1 SERTAD2 BMP6 FTH1 CEBPD LOC105376121 SGK1 MGC70870 NFKBIZ RGS8 TRPA1 PRDM6 MSR1 FMOD RIMS1 RPL24 IGFBP5 WEE1 PRDM1 ZFX C1S ND2 TSIX IGSF11-AS1 MIR5690 LYVE1 CLEC7A CEMIP HLA-DPB1 XIST LOC112268061 MGST2 ACVR1 ADORA3 SNRNP25 NDUFA1 HSPE1 C2 ZNF804B KANK2 LOC105377918 GUK1 LOC107984561 SPRY4 TLR2 LOC107986853 PHLDB2 BMP5 PDE4DIPP7 LOC105372093 SLC2A9-AS1 LOC105370525 LOC101927627 C1R RPL36A GIMAP8 TRIM22 RNASE6 ARHGAP30 SLC16A12 RNR2 HLA-E FOXC1 TFCP2L1 LOC105374936 LOC105375689 LOC101927069 C4A DSG2 APLNR MTHFD2 CCN2 RPL7 MYOF ZIC1 LOC105373581 TBXAS1 COX1 IRAK3 VSIG4 DIRAS2 LOC105376380 CYYR1 LOC107984658 ZNF599 LOC101929473 ATP5ME ZEB2-AS1 TBX15 RAPGEF4 HHAT HLA-F KDR ECM2 DAB2 SCARA5 INHBA DDRGK1 MIR23AHG RBM47 CHMP3-AS1 VXN LOC107986148 SIGLEC10 PAIP2B MS4A7 LOC112267956 SERPINE1 LOC101928491 COX2 RPS7 UNC13C NDUFS5 LOC105375532 TXN LOC105374768 SLCO4C1 LOC105375716 OLFML1 LOC107984770 PAH HCLS1 GPR15 |
| **Turquoise** | 2733 | AACS ELMO2 ABCA3 SLMAP TMEM216 PAX7 SMARCD3 CMTR2 ERCC5 ATF6B PFKP GFOD2 AEN NAGPA ZNF646 NHLRC3 LMAN1 ESYT1 TOP1MT MYO9B NTSR1 XAB2 PRKCG UTY MON1B CHORDC1 RP4-635E18.9 ZSWIM1 PNPT1 MIER1 CCDC97 KCNK3 AQP9 SEMA4D MMS22L AGPAT1 LYAR GRM2 KNDC1 LONP1 RRP7A TMEM132E PTCD3 CHD1 TCEANC2 UBA1 C20orf27 DHRS11 SLC12A4 GEN1 KIAA1033 LPAR4 TMEM180 TIGAR TRIQK RELT LMNB2 ABCD4 SNX16 FOXK2 RNF32 GTPBP1 GRK5 HES1 RBM17 MANEA GDPD5 PDCD4 CEP350 WHSC1 RAPGEF6 WDR45 SHROOM1 LTBP4 RBM10 KCNC1 MFF NCOR2 SIAH3 CNPY4 ZDHHC6 ZBTB48 SMUG1 CORO2A ETNK2 PABPC5 ING5 CHST15 CETN3 CCAR1 RPS28 GPN2 SLC6A7 CDIPT PLXNA1 TMEM255A SYT12 PMM1 BCHE SETDB1 GCLM INTS1 RNF166 TJAP1 MYO15B GMPPA PTP4A2 TAF12 COL18A1 TBC1D17 STPG1 ERBB2IP NIPSNAP3B COG1 MTMR14 ELAC2 CEP120 RICTOR TMEM104 HSF2 POMT1 NUCB1 SNAPC1 GNA11 CAPN1 FAM204A RASIP1 ZNF687 ANKRD49 ZNF552 CACNB1 QKI MAN2B1 EML3 ACADL PRDX4 ABHD12 CHRM1 UFL1 COL7A1 NFIL3 SEZ6 DNAJA3 GCA GLG1 CENPU ZNF227 ARAP1 AGPS QRICH1 COMMD5 DCUN1D5 ARHGAP15 FAM218A PRR5 DOCK11 STAT5A ZNF350 NFYA HCFC1 PBLD GORAB YIPF2 RTN4RL1 DUSP18 ARMC6 AHR E4F1 ACO2 ELFN1 WAPL IFITM10 PAQR4 OPA3 SKAP2 KRCC1 TRAF7 MAGOHB MOB1B PARG MICALL1 HID1 GPBP1L1 DUS4L STRIP1 ZNF738 ETFA CHMP6 PDAP1 POLRMT ZNF324 SCAMP3 CCM2L TNRC18 PRR36 RDX FO538757.2 CELSR3 FBXO46 EHBP1L1 CELF5 CAMK2B TNKS2 CXorf23 TRIM45 ATP2B2 LXN KLHL21 KIAA0930 KIF4A FAM161A ZMYM5 RCAN1 PROSER3 NTN4 SYDE2 SHH SH2B1 ACAP3 RAB20 TARSL2 POLR1A DPYSL4 TM9SF4 LRRC16B METTL5 USP9Y ACADVL JRK TCTA SLC29A3 RAB11FIP4 TAOK2 DAGLB EHD2 KIAA1551 SPG7 NUP133 CLK3 44991 CLIP2 FAM129B BEGAIN VPS16 IGFBP2 SULF2 ADD1 KDM6B ZSCAN12 MAPK7 NFAT5 TAF1D PCED1A FBXO3 CCAR2 VPRBP NOL4L ZBTB47 SDK2 BAIAP3 ZNF681 PTP4A3 PODXL KMT2B CDK16 FBXL5 BSDC1 RABL6 CPNE9 STX5 CDKN2B PROS1 XRCC1 SDR39U1 NYAP1 PARVB CSRNP1 PPP6R2 ELL APOL1 ZBTB41 AP2S1 VAT1 SHANK3 MAN2C1 SNRNP48 SGSM3 ARSA PHYH MUS81 UNKL AC069368.3 TMEM132A C19orf44 OTUB2 CHPF2 MCAT PIANP SPNS1 RPAP1 C21orf2 EPN1 RAVER1 SH3GL1 EIF3H MBD2 PIGT C17orf62 NDUFV2 CCDC28A TRIP10 PIAS1 TESK1 SFXN3 FSD1 GCLC HMGXB4 MAN1A2 SEC61A1 FKTN TWSG1 UPF1 ZSWIM4 BLVRB AC003005.4 CCZ1B ADCY1 EBP MRPL22 EFR3B TRPV1 WFS1 TBC1D16 CC2D1B GBA2 FBRS FMNL1 DEPDC1 SPATA24 ADGRG1 USP38 ZDHHC17 KIAA1429 QSOX2 KDM4A URB2 MCF2L SPEG TBC1D24 AAAS PTPN23 DGCR8 ZNF676 IGHMBP2 FAM73B KCNH3 PLBD2 UCKL1 OBSL1 GOLGA7B ZNF84 THAP4 MDC1 GRAMD1B NKAIN1 RB1CC1 POLR3A CSDC2 ABHD5 ZNF114 TCHP HEATR5A TRMT1 ZNF385C KMT2D SLC29A4 SLC35F5 CBWD1 RP11-618P17.4 ATP2C2 ACADM KCNQ4 XPO6 PIK3C2B USP15 ZDHHC7 RNF31 FAHD2B ERCC4 SLC31A1 ESAM PPP5C ELFN2 SCAMP4 NDUFB7 TMEM182 MIER2 AGRN FAM171B RTN2 PRPH2 APBB3 CHD7 RNF126 CEP63 GPSM1 CLIC4 TLE3 LRP1 THUMPD3 ELP6 SAMHD1 PTBP3 ATP13A2 NAGLU TMEM38A SNX18 MDM2 SDF4 KCNK9 AKT2 RP11-212D19.4 MRPL13 EEF1D AFAP1 BCR DZIP1 FXYD5 ZNF317 NR4A1 TRIM17 PURA DNPEP TMEM196 CREB1 ABCB9 FANCM TRAK2 DDX3Y OSBPL7 TARS2 SKIV2L2 FCF1 SMARCA4 ZNF624 RPS6KA4 LRP8 TGFBRAP1 TAGLN3 FAHD2A CENPW ZBTB21 PSD4 RGS11 TMEM43 HS6ST1 SGTA VIMP STAT5B C14orf79 SH3D21 NUDT19 SKI SHISA4 RPL28 TRIM11 MPP6 TBL1XR1 FAIM SP140 G6PD DDIAS DPH7 FBLN2 PPP2R2A ROBO4 FAM134C ZNF865 LPCAT4 THBS1 FARSA PLEKHG5 FAM206A CMTR1 CCNE2 TAZ CRIP2 MYDGF MPLKIP ABHD8 MAP3K12 TAF11 CCDC136 ZNF121 SLC3A2 MBTPS2 MVK HIST1H2AC UVSSA WDR44 CRELD1 C5orf30 RNPEP GCN1 TGS1 ZBTB10 BHLHE40 IST1 KANSL3 CHST12 CHMP1A HDAC11 PLXNB2 GRIK3 DGCR14 TNIP1 TCTE3 RHOG NSFL1C PDHX MTA1 ZNF720 LIN9 ZER1 INTS2 SEC14L2 POLR2A SDC3 TMEM218 ZFYVE16 CIRH1A MAPK11 CAMTA2 HIP1R SCN7A NAB1 ASIC4 DGKA TSPAN18 NADK TRMT10A ZNF263 TACR3 SLC35C1 LAMTOR4 NEURL4 KIF1A HERC5 C16orf58 EIF1AY JUP C2orf68 PRRG3 CHIC1 ACAP2 CARNMT1 FGF13 PPP4R2 CXorf40B NBL1 DAGLA RAB11FIP5 ZNF496 SCAF1 ZNF550 CA5B HCN4 MIS12 LIPC SLC6A12 PAK4 TBC1D15 FAM214B PHKG1 JMJD8 ISLR2 ITCH MGAT3 NCBP3 KLHL26 GTF2H2C GJC1 CELSR2 KIRREL STAM2 GPC3 TAF1C CIAPIN1 HELZ ZMIZ2 ZNF668 STAMBP ZNF737 CCNF SRM ANKRD33B SOCS5 SNX8 PLA2G16 DLG5 PER1 KLC2 SMARCAD1 ATG12 MICAL1 BLZF1 SWI5 ASB5 AREL1 KDM2B HARS2 UPF3B TRIM46 ADNP DMWD MBOAT7 SCRT1 EIF3E LRRC27 RAB10 ZBTB7B MRPL23 CNOT3 GAK SORBS3 CD101 LMO1 KIF22 UBAP2 AMPD2 RSF1 MKS1 PDE1B MYLK3 KCTD3 C2orf88 ANKRD13D COMMD6 DENND6B GALNS ZNF391 ZSCAN20 MAP1S SLC45A1 KRBA1 METTL17 LBH ZFAND2B TBC1D23 SAMD4B MAP2K3 ATXN2L SESTD1 FLII PRCC CCDC126 ZNF675 CHPF CALCOCO1 CWF19L2 RFC5 ZNF214 DDX56 RMND1 AKT3 ADCK5 UBR4 KTN1 ABTB1 CTTN RELL2 ARL5A TBC1D8B USP8 BAIAP2 45178 NR1D1 ARID1A HIST2H2BE SHMT2 ZBTB40 SDHA NCLN LYPLAL1 ALPL SMAD6 SAV1 TRIM62 MAP3K9 RPP38 MAST3 TFAP2E ZNF445 NRXN2 UQCRC2 RYBP WBSCR16 FAM171A2 DRG2 NSUN4 ARL6 TRMT2A DUSP8 LBR CBFA2T3 CDIP1 CCDC51 PLXNA3 FZR1 ANKRD37 DPH1 ST8SIA5 ZNF225 ZNF485 RNF122 FAM65A LRP3 IER2 MRPL4 PCMTD2 MBNL2 ADGRB2 AC064829.1 ATP6V0D1 SLTM RAP1A LZTR1 ARVCF POLR1C ZNF783 ZNF85 RECQL POLI CCDC12 ECHDC1 RNF170 NDST1 COL20A1 TPD52L2 PTK2B UNC5B SOX4 THOC5 SMARCD1 USP20 L3MBTL2 ZNF347 TPP2 PKD1 KIAA1522 PHKG2 SLC39A14 CLN8 GS1-114I9.3 CRY2 MECP2 RXRA TMEM60 NOS1 CIB2 STAG2 ARHGEF12 SCYL1 CTDSPL2 YARS2 TBC1D12 ZCCHC4 THAP10 ZNF513 MEPE PLCG1 ZNF43 ITGB4 MRPL19 ZBTB38 CAMSAP3 C14orf28 FLYWCH1 MINK1 SLC25A1 RAP1GAP2 MYO1C KIFAP3 PI4KA C19orf25 WIZ ACBD5 MGAT1 CCDC137 MIER3 COPA RASD2 IPO8 ZNF175 LRRC75A MAP3K14 PCDHB8 NOVA2 GOLPH3 PDE6D ZNF117 CLK2 FANCL HSD17B11 SPRTN HMGN3 FAM133B KCTD18 PAG1 SOS2 ZNF774 LETM1 APBB1 LIPE MPPED1 CXXC1 CD40 ZNF81 GATAD2A N4BP2 SIRT4 AC010642.1 EHMT2 INTS8 WTIP ERMP1 THAP8 ARHGAP33 TCF12 PACS2 MYOT SNRPF C3orf20 SCD5 ATG4B FBRSL1 GRIN2C MYNN DMPK TECR DSC2 LPXN IDNK TRIM65 SMYD5 BCL9L PEX1 RCC2 ALG12 KHK SLC12A2 RIPK2 NLGN2 ZNF566 ABCC4 PDRG1 MSI1 PPP4R3B CC2D1A MAGT1 FAM188A LLGL1 ARIH2 MTF1 HUWE1 FAAH PROSER1 LYRM5 KIAA1524 TM7SF2 FLOT2 SH3BGRL 45179 ADGRL1 VPS51 ILK ZNF724P GTF2F2 CDC42SE1 CDADC1 EMID1 ENDOV MORC3 MFSD2A RAPGEF1 GATS TTLL7 GSN DGKZ MYH3 SULT1A1 PLA2G4A MAPRE3 SENP6 AKR7A2 GGPS1 MAP2K2 HIST1H2BB ELN MPI P4HTM TMEM209 LZTS1 CSNK1G2 PEX13 FAM229B CACUL1 SHANK1 PDK2 LIN7A SVOP VPS9D1 RUVBL1 ADAM9 APAF1 SLC36A4 UBA3 DPYSL5 MPP2 TNK2 NFKBID STX7 BCAR1 TMEM214 ATG4D TGFBR1 WDR4 HEXB GALNT12 PPP1R16A PTER POMGNT1 CRLF1 MYO18A SEMA7A CCBL2 ATM TIE1 TRMT13 ARRB1 SCAMP5 TMED5 POLR3E AQR USP45 GABRA2 ADAMTSL2 TNFSF18 PCGF2 CALY TNS1 MDM1 RBL2 SRGN PYGO2 EIF3F EDEM2 LENG8 TOM1 DHX37 USP53 CAMK1G STYX LYPLA2 SMAD4 OSGEPL1 SLC35B3 RRP1B STAT6 ZNF195 PRPSAP1 C10orf10 PPP1R12C GRAMD4 ATG9A VARS2 NFIC ZNF787 PELI1 CELF3 UBE2B CCNT2 MED16 GOSR1 PLOD3 ZBTB39 TMEM63B ELMOD2 SHC3 KAT2A ARRDC4 MTUS1 TRAPPC10 KLHL25 RNF138 PIEZO1 DIMT1 GTF3C2 SOAT1 ATG16L2 EXOSC8 F8 KPNA5 ARHGAP23 IFRD1 PRR14 KCTD9 ZNF267 PRRT3 ATAD3A FBXL19 ZSCAN1 DFNA5 GABPA ZNF260 TLR3 CAMK2G HMGB2 USP37 PTBP1 CDC7 ZNF335 TRIM58 IVD GLMP GLRA3 SPRED3 RIPPLY2 C9orf114 LSS MVP ZMYM1 ZC3H18 HNRNPC SF1 FXR1 RAB11B CASP3 ZNF429 ZNF461 SUCLG1 CNEP1R1 PRPF40A EML2 DUSP11 SKA3 DHFRL1 SRCIN1 P3H4 TMA16 TIGD7 FOXP4 MOB3A PRDM11 GFM2 NAPA PHRF1 GNL3L KIAA1324L C1QTNF4 DUSP19 PRKAR2B RHPN1 LRIF1 EDIL3 INSIG2 FUT1 C19orf66 B3GALT4 COQ6 SELK TMEM9 SCYL2 TYW3 MYH14 OSTC GPATCH1 NT5C3A TMEM65 PCDH1 TTC31 TRIM67 EXOSC1 CDYL2 CHM SH3BP5L LYN GMPPB GDAP2 TIMM9 CDO1 ZNF491 FRMD8 WBP5 NTMT1 CNIH2 KIAA0907 SNPH NUCB2 B3GNT4 FPGS ANAPC2 KCNJ4 SFT2D3 ZNF354B CLEC14A SLX4 PALB2 UBL3 SEPSECS MED17 BCORL1 SASS6 BRAT1 ZSCAN2 TFDP1 SEC24C AXIN2 LSM5 ZBTB1 TUBGCP2 SPNS2 MYO15A CIC GIMAP7 PIH1D2 SAMD14 ATP6V1E2 HCN2 HTT TRAPPC8 CCDC53 DAB2IP GRIK5 DCUN1D4 USP21 MUL1 NUP58 AP5B1 ZNF766 KIFC2 PLAG1 COX18 OGFOD3 CCDC82 XPO1 WDR35 KCNN1 LATS1 ZNF777 BAZ2A CAMK2A KCNQ2 FADS3 FXYD7 PRODH ATG4C PHYHIPL RBM11 STKLD1 ZDHHC5 TRAF4 OPRD1 SACM1L CX3CL1 ARAP2 ASB6 FAR1 KLRC4-KLRK1 SPTB PCM1 GNPDA2 TSG101 HELLS IQSEC1 NAV1 AEBP1 DBF4B TUBG1 PLAGL2 HMGCL CORIN ACAA1 AC002310.13 PLEC SMG5 ZNF319 TMEM109 ANK1 PPP4R3A POLR1D GIN1 TMEM177 PSMB5 CDKN2AIP USB1 SETDB2 NIT1 TNFRSF10B FRK DNTTIP1 RELA RASD1 DBN1 FAM98C TMED7-TICAM2 DUT SLC25A19 SPATA2L SEMA4G PANX2 CFTR PPP4C NFATC2IP NOMO2 FAM210A PKN1 MED9 AFMID TMEM201 ETAA1 PHF14 FAM3A DGCR6 RRP12 BAZ1A SUPT20H ACBD4 MRS2 RHBDL2 SIRT3 FAM133A TMEM198 KIAA1614 DMAP1 LYNX1 SUGP2 ANKRD12 DDX51 ZNF626 MKRN3 SLC30A5 TUBG2 ACVR2B ZNF517 C3orf18 ZNF587 EXTL1 WDR5 RARG MYO7A C11orf24 TIMM44 SLC41A3 DGCR6L ILVBL MLH3 DNAJB5 CSNK1G3 FAM83D IFNGR1 RLTPR SRFBP1 MBIP ANGEL2 FAM162A RBMX FGL1 DEAF1 CIZ1 GPR137 MSLN DDX60L MARK4 CBY1 SLC35A3 ADPRM PEMT MCM3AP NIT2 PLCD3 PTBP2 ARHGEF17 CHD5 C19orf60 ZNF431 C7orf25 TBC1D25 ZNF800 TOR1AIP2 GPANK1 DDX58 CDC34 ANKIB1 CUEDC1 GNPAT MYPOP ZFHX3 DPY19L4 HEMK1 ERICH6 SCRIB TBC1D2 GRM4 ANKRD27 EIF3K TCP11L2 PLB1 PRR12 CPSF4 ULK3 WDR86 KCNMB2 POP5 GTF2IRD1 ARRDC1 ZNF613 ZNF714 PGLS COL6A1 ZFP69 MIOS DTWD1 KITLG SV2C GKAP1 SETX RNF19A WWP2 ZNF326 RGPD1 FBXL18 ZNF592 PPFIA4 KCTD21 GTF3C1 PLEKHM2 FAM208A KIF21B LMNA SLC9B1 IGIP ZNF250 MIEF2 WNT5B APLF PANK4 WNK1 DPAGT1 SSBP1 TESC GTF2H3 YTHDC1 PTH1R NOTCH3 TMEM101 NF2 PNN RP11-529K1.3 ANP32B PLXDC1 TMEM168 MPG FJX1 NUDCD1 CCDC112 USP44 NDC1 RP11-474G23.1 GOLGA4 ECD MTMR11 STXBP3 GOLT1B NEK3 MIB1 SUSD1 CARKD ARHGAP27 PKD2L2 URM1 TMEM106B LRIG2 PISD SLC27A5 MTERF2 TRAP1 GPC1 BTG3 PAK2 WASF2 CDK10 FCHSD1 PTGIS C12orf73 DPCD SLC25A53 RANGAP1 CCDC88A SGPP1 HELQ ITPR3 ZNHIT6 RXRB ACTN4 RAB2A CEP250 DLG1 RP11-505K9.4 UTP20 APOL2 BYSL TKT INPP5E U2SURP TMEM151A ITPK1 POLK ATP13A1 DAZAP1 PRKRA KIAA0895L STK32C CLUH SLC33A1 RP11-437B10.1 ANKRD13B AATK TOP3A SLC4A8 FGFR1 UNC5A ZNF181 PIKFYVE PI4KB ANP32E YEATS4 SYNGAP1 BET1L HMGN1 ZNF718 BZRAP1 RHOT1 45176 SPPL2B SHC1 ARL4A GPR153 TANGO2 HERC2 ZNF48 CACTIN HOOK3 LIMS2 GINS1 BAHCC1 BSN TBL3 CPNE5 OGDH ZFP14 C15orf52 CHRNA5 FAM63B ZNF529 DDB2 KATNB1 SPIRE2 GOLGA3 NKD1 MTRR PPIL4 MGAT4B PLEKHO1 MIB2 TMEM63C RING1 RBPJ ZNF484 DAPK3 IP6K1 RP11-343C2.9 BOP1 MAPK12 SLC38A10 DNM1 OTUD4 KBTBD3 PCBP4 RPLP1 SCP2 WBSCR22 ASUN IFT140 ALCAM FAM76B TRAM1 RACGAP1 SLC6A6 UBXN4 NUP37 CDS1 TMEM151B FKRP CELF1 NIPBL PDE4C PPFIA3 ESRRA PBDC1 FRA10AC1 IRF3 PRRC2B DEPDC5 RYR1 DLGAP4 PHLDA1 INO80D USP19 ACOT7 SUV39H2 ZNF728 ATAD3B TMEM87B HINT3 LSM12 ARIH1 POLDIP3 POU6F1 XRCC6BP1 SAPCD2 VASH1 AKNA MIS18BP1 HPS4 ZNF79 ZCCHC9 DIDO1 DHX15 PICALM DRAM2 STK40 PIGP DPF2 PKIB SLAIN1 MED12 TLE2 AURKA FRYL RHOBTB2 TBC1D9B PNPLA6 NOC3L RABEP2 DNAAF1 UBL7 CHRNA2 PPP1R12A GGA1 RBM38 ALYREF IQSEC2 ZNF672 TAB1 SETD8 ACVR2A ANKRD52 FAM83H GAA GDF9 MITD1 PFKL FAM189B ZC3H6 GABBR1 USP4 MOGS DEF8 PLA2G6 MRPS28 MTRNR2L10 GRWD1 BCKDK PXN CCDC77 RILPL1 COG4 CYHR1 SIRT2 DNAJC11 ZNF354C MEIS1 BMP8A FAM114A2 ZGRF1 HAGH TMEM179 GTF3C5 ITGB1 BVES DUSP14 AP4M1 KCNH4 SNX13 RAB9B CHST10 TRAPPC2 SLC50A1 C1GALT1C1 DPP7 GATSL2 SENP8 MED25 ZNF442 C19orf47 ASPSCR1 WIPI2 CCDC90B UBE2D3 REXO1 EME2 TMEM69 TMEM185B TRAPPC5 HAS2 TMEM229A GAS8 IMPACT ORMDL1 C1GALT1 USP35 TRIM35 ZCCHC10 LAMC3 MVD UBE2Q2 ARHGAP12 ZFPL1 SCCPDH OLIG1 NUDCD2 PLCXD3 IRS2 DENND2A KAT8 DMXL1 SH2D3C SRRT SNRPG ACOT9 RITA1 ADCK2 POFUT2 FRS3 ZNF354A GDPGP1 PCBD2 RASGEF1A TMC6 XYLT2 FLYWCH2 CDR2L GAL3ST1 CRAT PDZD7 POR SREBF2 FAM89A KIF9 PIK3CD PITPNM2 UXT ARPC1A SPATA2 ZNF530 C1orf131 NATD1 ORC5 RPS4Y1 TMEM259 NLGN3 PIGK RP11-701H24.9 SLC9A3R2 PYROXD1 TSPAN2 ZFAND6 NUF2 PWWP2A PPP1R3F FAM91A1 BROX PARS2 PIP5K1C TSPAN14 NAA40 FUT8 GFRA2 ZC2HC1A ZNF74 WWP1 ZNF90 ERGIC3 PTGER4 RECQL5 GCDH RGS1 NLRX1 PLXND1 ZFP91-CNTF BSG BBX FN3K DTX4 BIN1 PPP1CA TRIP11 CACNA2D2 DOCK6 SLC25A42 FKBP8 PCDHB6 OLFM2 UCHL3 IFT74 ZNF727 CNTROB HDDC2 INPP5K CCDC174 SLC25A40 IFFO1 MDGA1 VAC14 COL5A3 SOGA1 WDTC1 ZFP62 HEBP1 LRRC63 TTC19 ME2 ZCCHC6 DTX2 FAM126A SIRT7 PFAS PPIL3 MKNK2 CCS NXN FAM200A PRKCZ EIF5B YAE1D1 AAR2 ASL METTL14 PLD2 PALM PUM2 ARX LDLRAP1 EMC2 NAMPT SSBP3 TBCEL ZNF582 FAM102A MMD2 CTNNBIP1 MZT2B YAF2 BRPF1 CA4 LIAS PYGB NHLRC2 SBF1 GTF2A2 ZNF793 CCNJ PSD DHX8 NAE1 STIM2 NUPL2 UBE3B IQCE COA4 CD99L2 ATG2A PPP2R5B KIF12 PTPRF IKBKB TMED8 ARHGAP1 GPAT4 MEX3A ZNF706 RRP1 TFAP4 FCHO1 PGAP1 KIF17 POLR2F SIRPA TSC22D2 C10orf2 PRPF31 ABHD13 CPSF6 CLSTN3 NISCH DTYMK MARK2 BLCAP AMMECR1L ZNF501 ATF2 GABRD QTRT1 TNKS1BP1 UBXN11 PAOX RBBP8 CBX7 DNAAF2 ESCO1 PHF2 JADE2 FAM175B MAP2K7 ANKRD44 SYTL2 BMP1 DPF1 DNAJC21 MRPS31 SART1 MAPK13 RSBN1 RPS6KL1 DPP9 SLC43A2 EXTL3 LIG1 DNM2 ZNF616 PLA2G7 SUZ12 PDE6B CDK5RAP3 IL17RA TCEA1 SPA17 CAMK4 MRPS36 MZF1 PTPRN MPV17 WFDC2 COL16A1 LYSMD3 SLC29A1 TP53I11 PDXDC1 SRC CYB5R3 SBK1 SETD1B ZNF606 NFS1 CRMP1 FAM163B NUP107 ZNF507 PPRC1 PCDH17 TCTE1 RABEPK SMTN TMEM87A SCG3 CTD-2349B8.1 DDX59 ZNF143 MAN1B1 P4HA1 SP3 ABHD17B SEC11A C18orf32 MNT PLEKHD1 GINM1 PRPSAP2 STK11 ZCCHC11 C16orf87 FIZ1 LAMB2 FNTA IQSEC3 SUCO MED24 ZNF512B SRA1 PPIP5K2 ZNF469 ICE2 RP11-166B2.1 SLC7A1 ZNF746 AGAP3 KDM1B F5 CPNE1 ZC3H4 NUDCD3 FAM118B ABCA2 RABL2B DCAKD AGPAT5 LSM2 RAB39A DOC2B HINFP MAPK8IP3 IMPDH1 ETFB ELMSAN1 MXD1 TRPV3 SEMA4A ANO8 RBM39 KPTN VRK3 SIRT1 TSNAX-DISC1 LDB1 ZNF671 TUBB8 ADGRA1 CAPN15 ZKSCAN3 CTSA TPRKB VGF WIPF1 PTPRU COLGALT1 APEX2 TMEM107 DEDD2 PSMB3 FAM228A ITGA7 RIMS4 CPT1C CCDC159 KCNIP2 ZNF207 EXOC7 MPRIP NLE1 AP5S1 C11orf84 SREBF1 PWWP2B GPR68 ALKBH6 BEND3 CLTB HACD4 TMEM184C BSPRY CCDC152 ELK3 ENGASE P2RX7 SNAPC3 DHX58 MECR SEC24A B4GALT5 TRMT1L ZBTB25 FBXO28 MCMBP DTX3 ADAM11 ERGIC2 CCDC7 MGRN1 TCN2 ZFY HAP1 CENPT RNF123 MAP3K11 ZUFSP OLIG2 CLASRP FAM101B HACD2 SRRM2 LRTM2 GIGYF1 POLA2 EDEM3 RUFY2 SLC16A1 CPNE7 C4orf46 SYNGR1 REEP2 CAPZB PDE4A NKIRAS2 COMT CACNA1G AMER1 KCP SPHK2 GNPTG SCARF1 TMEM81 APC2 SLC6A17 GOLPH3L STT3B PVR ZBED5 LTA4H CCDC24 CERS4 ADGRB1 PITPNM1 CCDC13 FAM50A RLF PLOD1 MTFR1 NPDC1 SMNDC1 TEX264 DONSON SUPV3L1 YIF1B NBN PVRL1 RCBTB1 ZSCAN18 MICU3 MATK PTEN SUSD5 AGFG1 TBC1D14 PNKP ROGDI ATXN7L2 RDH13 SPTLC1 KCNF1 KCNAB3 GPR180 RP11-12J10.3 NOSIP MAGEC3 SOX13 KLC4 EXOC5 CHID1 ALPK3 IKZF5 VSTM2L ACAD8 GPR162 B4GALNT4 FASN ZNF219 MRI1 SMPD4 ZNF500 ACTL6A SLC23A2 GRK6 MANBAL LIN54 SNRNP70 MTHFR ZNF197 PTOV1 CSGALNACT2 CMIP KDM5C ZNF397 ZC3H7A RTN4R ZDHHC16 ST3GAL1 PHF3 EVI5L DVL2 KLHL23 DICER1 45184 ZNF148 SSBP4 ZNF141 GGA3 CTD-3088G3.8 CHST1 THOC2 HDAC5 TAF7L GON4L TMEM222 MOSPD2 PM20D2 REEP3 LMBRD1 PRPF19 NDUFS8 LSM14A TRIM3 PAK6 MXD4 THBS3 SMARCB1 CEP170B ULK1 MRPS11 MYO5B STOML1 FOPNL POLM NUP153 CPLX1 MYH9 TTYH3 CPSF3L RAP1GAP MEF2A NOP2 GPI RNF215 PACRGL SPOPL RP11-321N4.5 SLC4A3 TAF2 AGPAT3 CACNG7 KCNH2 TSEN15 DHX33 CCDC122 CCHCR1 TMEM161B NTNG2 ADCY5 LRFN3 FKBP14 E2F1 NPAT MORN1 GPATCH2L RASGRP2 HAUS6 RAB11FIP2 TSC2 NRIP1 TRO DHX30 ZNF707 CPSF7 HYI ACP2 CATSPERG ATP7A SIPA1L2 PLCH2 ISOC2 RAPGEFL1 RAB26 44992 DTX1 CACNG4 GPALPP1 SUN1 MAPT MRE11A CASD1 HSPA4L SMC4 ZNF527 ZNF771 FOXO6 ZNF441 MTTP ZNF138 KCTD5 OGG1 DUSP26 LITAF C12orf29 RPS19BP1 PBRM1 VILL MPND ZBTB43 C4orf36 BRF1 SMIM10 TCEA2 ZNF92 MTOR INO80E SLC2A8 STK38L SLC27A1 TTC25 C17orf107 SS18L2 SNAPC4 STAC2 CSK CTSS TUFT1 ZXDA TMTC3 PRKAB2 ZNF76 INF2 ANO1 CRB2 FURIN SOX12 KIF20B ZYX SF3A2 SMAD7 RHBDD2 GATAD1 RGL3 XRCC3 ITPKA BTBD2 DOLPP1 ASIC1 HR CCDC134 TTBK1 DENND4B PRR25 MPP1 CD2AP AK9 ZFP30 ADAL KCNC3 HSF4 POMK ZSWIM8 ARMC7 NSL1 SPRED1 CYTH2 CNNM4 ADAM10 GALC NAP1L4 TBKBP1 TTC32 ZBTB26 BAD LINGO1 ZNF546 ZNF644 CYP2E1 SMPDL3A OPRL1 IQCG MEGF9 CRTC2 PLEKHA1 CYB5D2 SUGP1 PFKFB3 ECSIT CLCN7 SLC12A9 MAP3K10 PDCD2L CRYBG3 ISG20L2 CABLES2 RNF115 USP2 OBSCN KIAA2026 GBA RFESD RRBP1 UBXN2B CSNK1D MED6 TPRN RALGDS RBM12B NDUFB6 WRAP73 NXF1 ERI2 SEMA3B NELFB ADSSL1 MSANTD1 VAV2 NEDD1 PRPF38B BCOR RAB36 KCNK12 PNPLA8 MEIS3 RP2 ARAP3 ZNF444 PICK1 CSNK1A1 SNX14 RPS6KB2 PEX6 TXLNA TMEM187 SLC35E2B MAP3K1 ZFP82 ZBED8 MB21D1 TXNDC16 VPS13D LAMA5 TMEM184B HNRNPH3 ADPRHL1 SKIV2L ZNF829 SURF2 KPNA1 SLC7A8 FAF2 FADS6 TMEM41B FAM149B1 DOT1L CLASP2 PIN4 PDCD10 RSBN1L TAF6L PVRL2 MED30 ALKBH4 GRAMD1A TFG CORO6 GMEB2 PSMD14 KDM3B FOXK1 MAST1 ADGRA2 EMX1 ICK ZNF316 DYRK1B PHLPP1 TMSB4Y SSPO CPA6 JAG2 DACH2 KIFC3 SLC25A25 ZC3H13 WDR81 ENG PRKCSH LETM2 ZNF140 BRD9 GIT1 ST3GAL2 SREK1IP1 SMYD4 HMGN5 ODF2 AARS2 NBEAL2 FAM195B MEN1 RP11-729L2.2 RARA MED4 IKBIP CARM1 SS18 PTPRS TAF6 ITGB1BP1 TAF1A EBAG9 ZC3H7B PDE2A YPEL4 ZNF276 RRP8 TTLL12 STARD6 ZDHHC8 KRIT1 ACTL6B SLC22A17 TBC1D13 SGK223 PRIM1 TVP23B ACADSB HTR1F AFAP1L2 GRASP ACAD9 RP11-514O12.4 CEP78 CPSF1 UGDH NUP188 PPEF1 MTG2 CPEB2 PHF6 JKAMP HOMER2 DNMT1 EPB41L2 ZNF571 SMARCD2 WIPF2 ZNF283 PLXDC2 CADM3 NLRC5 SUV39H1 PCF11 SLC7A5 MBD6 ACKR1 UQCC2 PACSIN1 TRIM13 NAT9 ZC3H12D MGA KCNAB2 FBXW5 MAP3K2 ANKRD13C CEP97 JMY UBA6 ADORA1 ZNF451 SEMA6C RTTN POLE2 ARRDC3 TBPL1 MRPL39 ZNF844 GRIN2D HPCAL1 HOMER1 PLA2G15 GRIN1 ZNF302 ITPA CHRNA4 RP11-724O16.1 JUNB WNT7A SSH1 CAD CKAP2 XPA FAIM2 CCNG1 HEXDC ABCC10 PPP2R1A ARL14EP ESF1 ZNF14 AIMP1 PPIL2 VPS18 SLC27A4 MTRF1L HSPBP1 LRRC4B ATP8A1 BCL2L1 ZNF674 HECTD3 BRSK2 CRBN PRR18 ATPAF1 NCBP1 FBXO41 ZNF367 SLC9A1 IGLON5 CRYZ FGD1 EDC4 MPZL3 SLF2 NOL6 STEAP2 TMEM234 CD81 WDHD1 SH2B3 KIF1C TMCO1 SIDT2 TRAF1 TRRAP H1FX SEMA3D ADCY6 OGFR TGFA MMP17 MYL5 ZNF569 HYKK LAS1L HSF1 ADSL C18orf42 MSH2 NCKIPSD NECAB3 ZNF180 NFKBIB MTMR2 PRRC2A CCNH STK35 CAPN5 OLFML2B MFSD12 RUSC2 NOTCH4 NSRP1 ZNF708 ARHGEF1 KLHDC10 LGI3 C6orf136 RAB6C NKAPL B4GALT7 RAB30 GBF1 TCF3 CCDC88C B4GALT3 ATAT1 PLPPR2 NUP54 CUL9 STAT2 ATP6V1C2 REL BEND6 RNF40 CREBRF PDCD11 IFT81 ZMYND11 CDH23 EXOG C19orf57 PDK1 NAF1 TBC1D10B LMTK3 SNAP47 GXYLT1 BCCIP MTSS1 DNAJB12 NCAPH2 JAK3 ETV5 CDK19 PITPNM3 PPP2R3C TKFC QSOX1 UBIAD1 CDC42BPB CYB561D1 ZNF488 RNMTL1 PLD3 SCAMP2 SGSM2 SLC6A9 POFUT1 EPHB6 RGMA TMEM145 FAM114A1 FDXR CDCP1 EFCAB13 PRKAB1 ALG10 ITPKC ST8SIA2 SNRPB MICALL2 SGOL2 ZNF253 CGN DUSP15 SYVN1 TUBGCP6 FAM160B2 SPATA33 WDR55 DUSP16 NUMA1 GIPC1 ELL2 KDM5D PSMB1 VIPR1 AC084219.2 ADPRHL2 RNF157 FBXO18 ZZZ3 ZNF112 ZNF385A IGFBP4 ATE1 CEPT1 RNF13 TDRD1 ETNK1 TSNAXIP1 CLNS1A NUP43 PRR26 C18orf54 MED22 CHAC2 RRNAD1 ZNF398 METTL3 VPS13C HMGA1 GEM REXO2 ZSCAN22 ZFYVE19 IRAK4 ECI1 ATIC ADAM15 STRN UEVLD PGM3 ZBTB49 DGCR2 C3orf38 SCAF11 JPH3 SLC38A7 OGDHL SPINT2 HSPG2 BAX CD274 FDPS RP11-176H8.1 POC1B NDUFS7 GGA2 TNS2 CTC1 SRF C2orf74 PGPEP1 STX4 SEMA4C SIN3B FANCB CACNA1I ZNF383 C15orf40 SNX21 ERCC2 BET1 HDAC6 FLRT1 ASB2 GRIP2 CDHR2 PCIF1 |
| **Brown** | 2382 | CLMP RPS17 PCDHB15 FARP1 FAM219A DPY30 FAM153A GLT1D1 CADM4 SLC25A3 BRIX1 PXYLP1 IFT122 SUMO1 ANGPTL5 NAP1L2 EPB41L4B CRNKL1 HS3ST3A1 HIC2 NOS2 TRIB3 DDX5 C2orf49 ATP5G2 KBTBD8 FAT4 TMX4 COX15 GDA THRAP3 INIP NINL WSCD1 TNS3 NR4A2 PPCS C4orf3 EVC DUS2 SNX33 FAHD1 EPT1 C9orf43 CCDC34 ACTR8 ITGA3 CMPK2 ZNF510 CDC25B TMEM19 WDR89 SLK C5AR1 NOL8 BAZ1B BBS12 PPP1R15B SLC25A46 PDCD5 AP1S2 RAB33B ACBD3 SEL1L3 RNF103-CHMP3 FAM73A ANKRD6 C16orf45 GFPT2 PYY PPIL1 DNAJC15 NOA1 STAU2 FAM98A VWA5A RAD23B CTDSP2 TIAL1 WHSC1L1 ROR2 UFM1 USP14 FAM174A DSTN FBXO31 FBXO11 ST8SIA4 RNF4 GMPR ALG2 HSPD1 POLR2B CCDC43 COQ9 GCC2 WBP4 COPS4 CBLL1 EIF1AX CAMTA1 SAMSN1 KIAA1324 C12orf75 PIK3CB TNXB SF3B6 FAM110C LIMD1 IPP TMEM59 SEC23IP DDX18 ZBTB11 LUC7L2 SRP72 PTPN5 LRRC8A THAP1 GBGT1 CDK17 TTLL4 SLITRK4 PDP1 SLC22A14 PILRA KDM4B ASNSD1 TM6SF1 CD53 NANOS3 MUTYH FAM234A NQO2 CH507-9B2.9 MAK16 LZTFL1 SUV420H1 HDHD2 GMFB GNB4 AGTPBP1 PCSK6 RPRD1B PALM2-AKAP2 IL18BP TRMT6 PSMD5 MAD2L1 ATP6V1C1 C16orf62 CHMP2B PLPP7 COQ10B ARID5A UBE2N SH3PXD2A LMO4 TAGAP MILR1 IDS HS1BP3 TFEC PPM1H EXOC1 BRD4 ZNF146 VMP1 ZDHHC11B MTMR6 UBTD1 S100Z KIAA0226L CUL4B AKIRIN1 PDGFRB IL17D NOXA1 ADCY3 APBA2 TTC3 SGF29 DLST OSER1 MYCBPAP TCP1 VWCE TLK1 TIGD2 FAM177A1 FAM160B1 MPC2 RNF7 PGM1 CPD KCTD13 TMEM256-PLSCR3 AC004076.7 KLHL28 FBF1 SET LRP5L LYPD8 ATP5H LAMTOR3 NUP85 EIF2S1 TSHZ1 RAB5A CASP1 SUSD6 GTF2H5 AP000350.10 TNNT2 RGPD2 CECR5 ARHGAP25 XIAP THOC7 IFNAR1 ATP6V0A2 DOPEY2 LAMTOR5 IMPAD1 C5orf56 SQLE OLR1 PLRG1 NPIPB5 INPP5B SNTA1 UBE2J1 TCFL5 GALNT9 CD83 SLC16A2 HHIPL1 SC5D ZNF136 PSMD12 HSCB ANP32A HPCAL4 KIAA0232 NUB1 SRGAP2C FAM199X ECHDC3 TMEM106C BTBD10 RABGGTB CUL3 C1QTNF2 FAM122B ESRRB ZFHX2 GEMIN6 JAGN1 NDUFAF1 PHF12 TMEM263 OSTN MORC1 RTP1 C2orf69 GOLGA6L9 MAST4 EEF2K TRMT2B WDR26 DNAAF5 SUPT16H ZNF839 IL10RB ALPK1 MRPS18C ISCA1 ZFP1 SLC35A1 GPR160 COCH PCYT1B RALB RPL36A-HNRNPH2 ATG3 KLHDC2 PANK3 PSMA5 MAPK1IP1L USP51 PYURF TSEN54 ZNF611 CEP104 ZC3H14 CTDP1 WNT16 DDX50 SNW1 STYXL1 RPP30 SLIT1 SETD3 MKKS ACOX3 MMP24 RPS6KA2 GPBP1 FAM84B ZNF287 C11orf57 RFK ZFYVE9 FOXG1 TCTN3 PABPN1 DNAJB11 SLC22A5 GRIN3A PTCD2 SMOC2 ARMCX3 MAGEF1 PPARD YBX1 PHAX FH STK10 ARMT1 PAQR7 FAM20B FAM3C DCTN4 CCSER2 ZNF775 EIF2AK2 HNRNPH1 ALDH1L1 CITED2 FCGR2A METTL18 EIF4E CFAP36 ZDHHC1 CRHR1 EMB ZFAND1 RINT1 BRCA2 PPP5D1 GIPC2 FMR1 CCDC117 FAM8A1 C7orf50 C9orf72 TCEAL2 IFT172 GHITM ATPIF1 PSMB7 KLK7 API5 ANKH RFT1 SH3PXD2B ARL17B FAM117A SLC17A6 DYNC1LI1 EID2B IPPK BIVM KLHL15 RAB2B GNAI1 VPS4B SLC25A4 CYB5B NDRG1 FBXO9 ACOT11 RAB6A PRKD2 PAPOLG PUS3 PAPD7 CYSLTR1 TMX3 SEC22C GPR161 TWISTNB RNF146 WDR74 PCNP ATP5G3 TRAPPC11 LRRTM2 NAP1L5 BTBD1 ZNRF3 ZNF211 QRICH2 NFE2L3 TTLL9 TMEM181 CCNB1IP1 ZBED9 PSMD6 SSB TMC5 FKBP3 SLC22A13 ZNF544 SEC61G C2orf72 D2HGDH BIN2 DEGS1 ARHGEF4 SZT2 RPRD1A EIF3J TMEM167A BCAT2 TMEM39B CAMSAP2 PPP1R3E ARMCX1 HCFC2 NCOR1 CMAS RP11-411B6.6 MOCOS C14orf93 ZNF433 TXNRD1 KLHL18 BTBD11 CWC22 USP33 SLC24A4 PIWIL2 TRIOBP CABIN1 RBM5 NCL GUF1 HRH1 PRKRIP1 NEURL1 MED21 CDAN1 SYF2 DCAF13 RPL9 RAB32 RAB22A ID2 DDX21 LRSAM1 PWP1 DHX29 CACNB2 METAP2 TM9SF2 PSKH1 MED15 CLCN3 NABP1 VSTM2A CCDC173 SNX22 MSMO1 MECOM EMC6 TOMM70A SAMD15 ZNF559-ZNF177 MED31 MCF2 ZNF532 RAP2A ATP6AP2 AZIN1 ABHD10 UBE2A NOL7 BRMS1L TM9SF3 RNF139 MAFG SHANK2 RAB40C CBX3 SLC39A10 KIF3A KCNV1 PDIA6 ACP1 PTGFRN CDC23 ZMYM2 TAB3 KIAA1586 NEUROD6 FBXL3 PUDP CEACAM19 ZNF830 MRPS21 ZNF567 IMPA1 B3GNTL1 TCEAL3 KLHL42 SOSTDC1 GABRA5 CCDC71L TMEM133 PIK3R1 CFAP97 MAF HOOK1 MRPS6 NFYB FZD3 ZNF135 ZFYVE27 CNIH1 BMI1 RNMT LMCD1 JAKMIP3 SARAF MIA3 NEK9 ZNF789 DCHS1 RPL23A TUBGCP5 IGSF6 C14orf142 VIPAS39 BTD SNRPB2 POM121C APBA3 SKIL HMGCS1 ZNF814 POLR3F ZNF570 MARS2 DHRS1 ZNF821 SFT2D2 CRTC1 UPF2 EFR3A SEC61A2 UCP3 FOXF2 DEXI CDKN2AIPNL OST4 CKMT2 ACTN1 CYB5A NRBF2 CX3CR1 LIN7C NXPE3 FZD4 STX6 FAM20C DNAJB14 WRB TPR ABCC8 LYRM7 NOXRED1 TTC33 STMN2 HSD17B14 PTDSS2 ALG1 MAPK4 FAM13B SFXN5 SETD4 HNRNPU DYNLT3 OMG ZNF486 NARS TMEM215 SMAP2 CSNK2A1 TRAM1L1 AAK1 RPL14 JRKL EEA1 LTBP3 DDX20 UTP15 HSBP1 PASK CCT6A ZNF776 LAPTM4B SYT4 PARD6B NEK2 RCN2 MLYCD NDUFA5 TMEM206 RCBTB2 ACKR3 ZNF22 CPSF2 SRSF12 C15orf61 PIGM ZADH2 GNB3 ACTR2 MEF2C PSPH DARS2 COG7 MMS19 B3GNT5 PTGER3 ERGIC1 USP30 ARHGEF10L ACTR3 UGCG STT3A ATRX THEM6 ARV1 MACF1 NUFIP2 AXIN1 MEX3B KIF7 CCDC110 FAM127C CLGN METTL23 NLRC4 ENPP4 KCNE1 DEPDC7 MMRN2 PTPRE FAM20A AGAP5 NXNL2 ZNF230 NUDT10 C5orf24 IFI27L1 MFSD6 SLC9A5 SERINC3 RHOF CALB1 GTF2E1 ZFP3 ARHGAP17 KCNIP3 KCNT1 MSL3 ACACA ABCG1 GLUD2 RPS23 B3GNT9 RPP40 SCOC YOD1 SMAD3 RALY NUP93 LNPEP NAPG ZNF669 YIPF4 COQ7 CHMP5 FAM192A CCDC141 YTHDF3 SKP1 PGRMC2 PAFAH1B1 OSTF1 FYCO1 CTIF GLRX2 DOCK8 ARL5B AKIRIN2 ARMC1 SLC15A4 PRKCI CYSLTR2 RPL12 ZC3H3 COMMD2 CRYAB CHEK1 TRNAU1AP ARFGEF3 KIF11 SLC40A1 FAM102B COA6 UBLCP1 CCT2 C6orf203 ARF4 LMF1 KBTBD6 URI1 USP16 LARS GPCPD1 ZBTB2 RAB14 HMCN1 LY86 DYNC1LI2 TCEAL8 ZMYND8 VSNL1 CCDC186 PTGES3 CCM2 ZNF311 IFFO2 PRMT2 MED10 STRIP2 EXOSC10 COX17 COA5 GPR22 WDR5B BAG1 VPS36 CORO2B PAN2 RP3-461F17.3 TMEM74 CRHR2 RBM19 SNX10 ZNF366 IREB2 PDCD7 GRSF1 DNAH1 EXOSC9 PSMC6 GPR12 FAM171A1 BATF3 RNF2 DNAJC7 TM2D3 NOL3 ASAP3 BHLHE22 TMPO ACCS ARPC5 MAP10 PDE9A PRPF4B NRBP1 LEF1 PRR14L CCNL2 EIF2S3 VBP1 MOCS3 TBC1D2B RPAP3 TMEM8B SMARCA5 WWC1 PLEKHA3 CPSF3 RAB23 ARPP19 RIF1 ZNF516 DBT CGGBP1 RFX1 SAMD8 UQCRB SUMO3 ANO2 LNP1 HPS3 NAA50 MRFAP1L1 ANGEL1 KIF21A ABCE1 UNK RHBDL3 RSPO4 SCD EGLN1 MAN1C1 PHF19 ADAMTS14 NR1H2 STAT3 C3orf17 PPP2R5C SLC25A29 WDR36 TPCN2 TRIM26 MBD4 TSSK3 SPIN4 DDX52 GPR176 NDUFA4 PTCHD2 EIF5A2 ATXN3 NDUFB5 ADO N6AMT1 FBXO5 DPYS C1orf52 NPIPA1 LRPPRC EBF1 MRPL35 SNX2 RBX1 COL4A3BP WAS ZBTB7A LTN1 RP11-444E17.6 CENPA NDFIP2 THG1L C14orf166 GOLGA5 TMEM183A SSUH2 SMPX OVOL2 VDAC3 CSE1L CENPN C3orf80 MRPL50 C4orf33 DOK6 DFNB31 SLC4A7 CCDC40 GART SCARB1 RP11-468E2.1 ARMC10 TTC7A DNAJB6 DHX34 CAMK2N1 RP11-514P8.6 ELP3 QPCT HIAT1 SLC35C2 PLK4 RFC4 NUP50 CCDC130 SPCS1 MRPS30 PLAA EGR3 ZNF184 SEH1L ORAOV1 WDFY1 ZBTB6 PRR11 B4GAT1 PDIK1L ZRANB2 RPL36AL RAP2B POLB BRD1 CSRNP3 MTMR9 MTX3 TOPORS RP4-559A3.7 BCL7C DHRS9 ZCCHC14 MVB12B DNAH2 NUP210 FAM200B DHX9 NCKAP1 WDR47 ZNF333 CCDC149 WDR31 RGS2 RTN4 GTPBP8 IVNS1ABP NDUFV3 PPP1R2 UBXN8 MRPL18 GRB10 ADGRD1 ZDHHC11 P4HA2 RP11-302M6.4 NIPSNAP3A SMC2 NOCT TMEM51 LURAP1 SLU7 VPS37C NIFK MRPL32 CAPZA1 DNAJA2 ZNF512 ATP5L NUDT9 MRPL3 IGSF10 ZNF732 NAP1L3 BMPR2 B3GALNT1 EAPP C17orf58 MAPK6 MGAT2 GPR183 HIVEP2 SDCBP NUDT11 CUL5 MTA3 CERS5 TAF13 NOD1 CDH22 INA POLR2K PABPC1L TMPRSS5 STIL ENPP5 RPL5 TOX4 HSPA9 HCCS SLC35E4 GTPBP4 SLITRK5 CXorf57 SYT3 METTL22 TEX35 AP1G2 UTP3 EFHB C16orf91 BRI3 SYK HNRNPDL DHX57 MYCBP2 FIGNL1 CCDC65 C14orf1 PAFAH2 ZNF300 SECISBP2L 9-Mar LEMD1 PDZD8 NEMF PABPC4 DDX6 DIAPH1 AP3M1 CEP76 SLC4A1AP COPS8 AKAP9 KLHL12 SF3B1 IPO7 PATZ1 TTF1 THNSL1 SHROOM3 CCL28 LRRC40 TM2D2 NCAPD2 CCBL1 DCP2 SMU1 KRR1 PC GPN1 FAH UBE4B RABEP1 ACAD10 WNK3 SMIM14 TXNDC9 MAP2 EDEM1 IBTK RP11-178L8.4 FAM43A GSTM3 CAMK1D LOXL3 KMT2A GDI2 SPON2 FGD5 ACSF3 SELT ARL3 MTCL1 BPHL C2CD2 S100A10 WDR76 MEAF6 ARID3B MAP9 CDKN1C HSPH1 MBLAC2 NIPA1 KIF24 UBA2 RAB12 RBM18 RGAG1 ZNF185 TTYH1 LMO3 UBQLN1 RP11-231C18.3 RANBP10 PSMD1 DSEL UBA5 TMEM242 COPB1 RNF219 MRPL21 SMG6 INSR CDC123 GLIPR1 ITGA11 CRACR2A PPP1R16B NFX1 CIT CLEC16A MMD NOG RAB11A KLF15 PCDHA1 CACYBP CDV3 ZFR VANGL1 ACSL6 CSRP2 UGP2 EIF4A2 RRM2B PABPC1 STARD9 GPRIN3 CRTAC1 RSRC2 FBXO45 CDC42SE2 EIF2S2 UCHL5 MLXIPL SMIM15 SLFN5 ST13 PTP4A1 ARSE CARF PPIE GTF3C6 CCDC22 RARS HIST2H2BF RLIM SAMD9 KCTD12 UBE2V2 PPP2R5E COL5A1 TRMT10C ZNF266 DLAT CES4A CDC5L TMEM119 KHDRBS1 ZNF12 SRSF5 OLA1 MFHAS1 PPA1 ATP5E SERBP1 CYP4F11 PDHB LHX2 VTA1 CRISPLD2 MTMR3 SESN2 RASA3 CDKN3 RSPH9 SEPN1 DHX36 NDFIP1 APPL1 WAC ADCY9 ATPAF2 TMF1 PPP4R4 CELF4 ST8SIA6 TDP2 P3H3 ATG14 ZNF600 RASSF1 PITPNB LRP11 RP11-463D19.2 USP1 ATP2B1 TCEAL1 CMTM7 MS4A14 CENPF GARS KBTBD7 PNKD ZNF200 FBLN5 PDE12 ARNT2 TRAPPC6B BIRC3 SPOCK1 PIGX PYROXD2 IFT52 TACC2 CNBP AHDC1 ZNF785 CSPG4 NUP155 RBM15 CYCS GNAL GYS1 LYG1 PEBP4 PAIP1 NSMCE4A TAF9B TRIM33 NDUFS1 FBXO42 NDUFAF4 SLC16A14 COX6C GPR143 RCL1 RAB40B ARF6 CXADR BLOC1S2 RIOK1 ITM2B STK26 CAND1 ACTR10 ACSL3 SDAD1 TLR1 SEC14L5 SMC3 PARP16 PDIA3 MACROD1 NT5M ZCRB1 TAF5 KPNA3 HSPA1L CES2 GNAS TSPAN13 RP11-286N22.8 BCLAF1 TRA2B ARHGAP39 KIAA1715 RTF1 DHRS7 IL17RC LRRC8D METTL2B CEP170 CABP1 LMOD3 SRSF6 QDPR SLC6A15 PRDM16 METTL20 EP400 TRIM2 ATAD1 FABP5 ZNF25 YWHAQ FAM98B PPM1K YME1L1 HNRNPR TPCN1 TMEM120B ZNF75A KL SON ABHD2 MZT1 RHEB C4orf27 OTUD6B TVP23A NPM1 PRPS2 TMEM200A CCT8 ADCK4 FYB EIF3M RANBP6 PTMS FUCA2 PHF7 PIK3AP1 CRIPT MAPRE1 ZMAT5 TRIP12 UQCC1 KCNE3 APPBP2 CCDC181 KIAA0355 CNTFR ABI2 WNT3 TMEM14C SEC11C CLSTN2 OPRK1 WDR20 C12orf4 MOB4 ITFG2 SLC35A5 LEMD2 NDUFC1 NBR1 KIF2A GLA UBXN7 AGGF1 HLF ADAMTS2 ARPC3 WDR12 PJA2 COLEC11 ABCC11 FXN TIAM1 RPP14 FAM126B ZNF711 ASH1L VAMP4 BCAP29 ZNF583 TPMT FRMPD3 NEK6 SORCS2 C5orf47 YWHAZ PGM2L1 ASTE1 ZNF805 ACSF2 OPA1 ECE1 C6orf62 ASAP2 LSM3 VIT CTC-554D6.1 CCDC6 SPCS3 RASSF4 TPT1 SEC63 VWA2 TDRD10 C14orf178 ADRB1 MOSPD1 POC1A RAD1 NFRKB FANCA PHYKPL TIMM17A PCBP3 CHAMP1 HNRNPA0 EEPD1 ATP8B4 TTC30B SEC13 WDR91 YPEL5 KCND3 CREBBP RABGEF1 SVIP SLC39A9 RHCE NKIRAS1 GPATCH11 SPP1 EPS15 WNT5A TRABD2B AC003002.4 CBX1 PTGS2 LRP5 WBP11 C17orf80 ZNF561 GALNT2 HPGDS COX7B WNT9A ZNF362 FCGR3A H2AFY2 VEZT CNOT7 DNMT3A NKAP EIF4E1B PSIP1 NOL11 BMP2 IPO5 ZNF565 BCAS2 ICT1 CEBPZ SIK2 FAM53A GAP43 SAYSD1 PRKACB GNG2 HCG27 NTPCR CAND2 TRANK1 UBE2K SPRYD7 RCN1 GXYLT2 TEX14 XPOT SLC19A1 RP6-24A23.6 TRUB1 PAK1IP1 DERL2 ACAN ZXDC USMG5 ENOPH1 FHAD1 RUFY1 TXNDC12 NAA15 ATP6V1E1 SAR1A BIRC2 TMEM53 PLS3 DAG1 PTPN9 RPS24 KLF9 TPST2 CD86 ZDBF2 RP13-512J5.1 TSPAN31 FBXO25 NNMT NUFIP1 FAM104A SNX29 LRRC39 STAT1 TBC1D10A KIAA0556 C8orf59 LRRC36 GABRA1 SMARCA1 KCNQ1 EPCAM NR2F2 SPAST CCDC47 ZNF862 SRRM1 ZNF33B MOCS1 COMMD8 NAP1L1 IGSF21 ECT2 GALNT15 PTPRC HMGCR VPS29 KLHDC8A GPR155 FAM21A RWDD2B SHC2 DUSP12 BLNK VASH2 BUB3 COPB2 CTR9 CDC27 SIKE1 KDM5B BPGM TCF7L1 UBASH3B GFPT1 FAM207A KPNA4 GLO1 NUCKS1 IP6K2 HNRNPD TMEM68 LEO1 CENPE CCR6 BPNT1 ACTR3B TFAM PHC1 KCTD4 GTF2H1 PCNXL4 HECTD1 HDAC4 RPS6 KIDINS220 MPHOSPH10 G3BP2 GADD45A TSC1 UTP18 TMEM70 C1orf50 RCHY1 C10orf128 TACC3 DNAJC5G FAM57A TCEAL7 DCAF6 EID1 ZC3HC1 SPTY2D1 SPOP RASL12 DAZAP2 ZNF808 FRG1 TYK2 ADAM28 NRL ZNF268 CBR3 PRICKLE4 SRD5A1 RIOK2 UFSP2 HSPA14 ATP1A4 MUT AF011889.5 METTL2A C1QTNF6 BRD7 PSMG1 TCEB1 BOD1L1 HMG20A ARL6IP5 WDR53 EVI2B ZNF880 RP11-577H5.5 NCKAP1L SYNCRIP DNAJC8 SRP54 OSBPL1A ZNF589 FAM179B RFC1 TUBA1C TRNT1 RAI2 KIF1BP SRD5A3 ZNF423 MTDH ARSG ZCCHC17 LMBR1L IKZF4 GUSB PMPCB NID2 HYLS1 EHMT1 GLDC LSM8 DLGAP3 CEP19 KCMF1 TMEM74B ZNF415 WWC3 YTHDF2 ST5 MPP5 CLPX NPLOC4 ROBO3 FAM76A GGCT HTATSF1 ST3GAL4 DCPS SHOC2 MINA SETD9 SLC25A36 ARFGAP3 RPA3 AK6 C12orf45 PHF8 ZNF396 RDH14 HKR1 IFT27 TWIST2 PKIA SEC62 DNTTIP2 SBDS TIPRL GPR85 DDX1 AKAP8L LRRC20 FLRT3 GAB3 COL4A1 SEZ6L ISOC1 TBC1D7 GLI2 ZFAND5 PPP6C SPAG8 DZIP1L PURB ERVMER34-1 BAG5 FAM71F2 TATDN3 CASP9 EID2 ZNF615 BAG4 LAMP2 DGKE RP11-894J14.5 CPOX IGSF9B CNOT8 GORASP2 USP36 PIAS4 DNAJC17 S100PBP BNIP3L LCP1 IGBP1 GALNT8 WDR48 ACACB USP12 CUTC PREPL MRPS25 ZMAT2 CD47 C9orf40 RAP2C NMU ASXL1 WDR37 AZI2 XRCC5 RAB1A FAM104B SHISA7 ZNF620 WHAMM USO1 NUDT4 RUFY3 MRPL42 FBXO38 ABCC1 TMEM167B MFAP1 CDKL5 COQ4 C5orf22 MMADHC IGF2R AFTPH ZMPSTE24 CWC15 PFDN4 UBE3A C11orf96 C1orf228 TOP2B DNM1L ORC4 MTIF2 TEX29 DNAL1 PFKFB4 MED19 LRRC57 RNF11 PLPPR4 HTR2A RAB18 POLR3GL ZNF860 TPH2 ZNF91 LPIN1 JDP2 SERP2 MORF4L1 MLLT11 PEX12 ZC3H15 ZNF233 TRIM16L SEC61B RRP15 SBNO1 RSL1D1 GNA12 ARCN1 AKAP11 GLB1 GAS6 EPHA10 IGSF22 MOCS2 EPHB2 PLEKHA6 CALM1 COL27A1 BHLHE41 LACTB EPM2AIP1 SYT16 RBM28 ANKS3 PCSK7 CEBPZOS TRIM36 RALBP1 GATC RANBP9 EVL NUDT21 ACSL4 TRMT5 PPP1R7 C18orf21 ZFAT SYAP1 ZBTB17 B3GNT2 AP1AR ZNF780A DCAF17 IFIT3 L3MBTL1 RPL39L MLXIP RRN3 C3orf14 CLPB RYR3 SLC30A9 CCDC25 NDUFB1 RP11-382A20.3 FGL2 WASL ADAMTS18 TOMM20 FBXL12 EEF1E1-BLOC1S5 COL22A1 GTF2A1 GUCY1A3 TC2N MRPS35 MATR3 PACSIN2 LGR6 DCK ZFP28 HLTF TOPBP1 C17orf67 VCPIP1 KLHDC4 DOCK2 TRIM23 ARL1 FABP7 GOLM1 KCTD15 NR2F1 PLEK LYRM2 RAB8B TAF7 CROCC PSMA3 FARSB SOX21 LYRM1 RNF141 TBK1 VPS26A NR1H3 PAIP2 ACAT1 PHKA2 C7orf26 RSL24D1 PLK1 TTLL6 MIS18A ATP8B2 P3H1 AP000304.12 BTG1 ARMCX5 HIF3A CAPZA2 NDUFC2-KCTD14 LPGAT1 SFR1 HENMT1 SEC16B NPAS1 GPR173 PCMT1 SOCS6 RAB37 YWHAE SGPL1 FRRS1L CREG2 C5orf15 LILRB4 MGST3 CDKN1B SLC25A16 TNFSF10 EDC3 MBOAT1 IGSF1 SLC2A3 FEM1B ACOX1 TWF1 CSDE1 CENPH MYEF2 ZNF836 TRMT10B FAM216A SGCB COIL TMEM217 CASP8 TBC1D22B GTF3A MAU2 KRAS TMEM261 C15orf65 TMEM44 HSPA4 DHX40 ZWILCH ANKS6 ZNF639 GNB1L PPIG SMIM10L1 NRAS RPL22L1 MGAT5B TOM1L2 ABHD15 MNDA RAB21 C10orf76 MYT1 UHMK1 USP9X VAPA TIGD1 FAM169A GUCY1B3 USPL1 CTD-2331H12.8 BORCS5 ACTR6 CHCHD7 PPP1CC YY1 TARS AASDHPPT SLC19A3 CDC37L1 FBXW4 BNIP3 C2orf82 SNRPE NBPF20 CARD6 IL13RA2 TXNRD2 HTR3B C11orf71 KSR1 NME6 VAPB SNAP25 MORF4L2 LRRC29 FBLN1 MRRF RAF1 SUB1 SGSM1 ZNF281 SMIM12 ANKRD46 JAKMIP1 SNX4 CNRIP1 LMBRD2 SYNRG SMOX DRD2 COPS2 RTFDC1 SUGT1 KIAA1107 CTD-2006C1.13 ADRA1B LPCAT2 BZW1 CLDND1 PIP5K1A PCNA PCDHB2 PTK7 B4GALT6 ZRANB1 SARM1 NYNRIN EPRS SGTB CHUK GPN3 PCDHB4 STARD3NL ZNF234 MYLK4 CLVS2 C14orf159 DNAJA1 ZNF124 ASCC2 FBXO17 GFM1 RAB11FIP3 PLEKHA7 AP5M1 FAM168B SIPA1L3 CCSAP MRPS22 CAMLG PPP2R2C P2RY12 CDH26 VWF PDS5B DIEXF FLNB WDFY4 MTIF3 TECPR2 PKNOX1 LIG4 SYCE1 OSTM1 TXNL1 AGFG2 HEATR4 RNF168 NAPEPLD CISD2 STRAP RND3 C20orf196 ESD COQ3 NDOR1 CDHR3 ERLEC1 PRMT6 CIR1 TAF4 GABPB1 PRSS3 HPGD DNAJC12 ANKRD24 CXXC5 PTCH1 TADA2A SMIM19 RHCG OCIAD1 TCEAL4 DDX46 AK2 BHLHB9 HAUS8 SEMA4B FANCF BEX4 CUX2 TADA1 DCAF16 FAM92A1 AP3B2 DIS3 ABHD17C SPIN1 ZNF813 DROSHA EMC7 PSMC1 LAMC1 SRP9 ZBTB8A GPR88 MKRN2 TLDC1 EPHX2 ELOVL4 ZMAT3 RAN VIPR2 PDCD2 MRPL44 LIMK2 PRLHR ETF1 GAR1 RPF2 MTPAP ING2 INTS3 TRPM4 IDH3A HSP90AA1 ZFR2 RANBP3 B3GAT2 UBE2G2 TRAPPC13 ACSM5 ZNF618 GOLGA7 SEC23A CLOCK SLC35A4 LGALSL PSMA4 SLCO4A1 MCTS1 TAGLN STK11IP ARPC2 TAX1BP1 ZDHHC18 WBP1L C10orf88 UHRF1BP1L RNF207 MMP28 EFCAB10 CNNM1 PRKRIR HSPA13 TMEM158 FASTKD3 ANKRD16 PML BRAP MARCKS SACS RBCK1 ZYG11B TLE1 PLXNA2 MGLL PNRC2 CD24 DENND2C NFASC ERICH2 HERPUD1 TFB2M METTL9 PXDC1 SEPP1 RPS20 RAI1 LOXL2 NPEPPS DENR RPL21 SENP2 XPNPEP1 KLF8 CDK5RAP1 GPR27 CAPRIN1 SORBS1 KREMEN1 GSPT2 ZBTB33 SPTLC2 SRSF10 EXPH5 SYMPK LEPROTL1 CLINT1 REEP5 FGF9 STX12 C11orf87 ENY2 PTGR1 KIAA1671 ANKFY1 EMC4 CREG1 IFIT1 4-Mar CMPK1 RWDD1 TSHZ3 RALA H6PD GNPNAT1 IGDCC4 MMAA ETHE1 GNPTAB RPAIN LPAR5 HNRNPM PLEKHM1 ZNF346 KLHL8 DNAJC2 NECAB1 MICAL2 ERO1A PLPP6 MICALCL MTHFS ANKRD50 ICAM1 RAPGEF3 EIF2A JARID2 KIAA1549 STK4 MICAL3 PCDH10 TMEM164 FGFR1OP2 HDAC2 DNAJC28 PAFAH1B2 HSP90B1 TCEAL5 SCRN3 TRIM44 SDHAF4 ATP6V1G1 BANP NPR2 TMEM170B ZFYVE26 AP3S1 PTPRA LYPD5 DR1 TMEM161A CYLD WTAP WSCD2 SERTM1 MGAT4A NSA2 EIF4E3 1-Mar GABRA4 RAD17 TMEM17 CFAP161 TSPAN9 ABCA4 RNF214 THUMPD1 ARID4A KCNC4 RGS9 ACIN1 FAM84A CEP41 SDHC CCDC61 IL16 MSTN CLEC19A VCL FYTTD1 ZNF470 MAP1B AKAP5 GGNBP2 LPAR6 CTBP2 OSCP1 LLPH ZNF525 NCALD OAF ALS2CL C8orf76 DLD ILKAP HBS1L CUL2 HSPA1B SLC39A11 GSTO1 HNRNPA3 LAPTM5 CETN2 IARS2 CAB39L COX11 PCP4 SYT17 CISD1 ZNHIT3 SNRPD3 CXXC4 FBXO48 TJP2 SERINC1 KRT10 PSMD7 SSX2IP RAD21 KSR2 C1orf204 MROH7-TTC4 ZNF654 POMT2 SS18L1 ABLIM3 CABLES1 ZNF791 UBP1 MORN2 ADAMTS10 CERS2 PRDM15 ZNF330 FAM193B ZNF770 FZD1 GMPS ATP6V1D CARD19 RPF1 SNRNP40 NDUFA10 NUDT15 SNRNP27 TEX30 EPS15L1 MGEA5 BCAT1 SLC2A9 CHN1 TMSB4X RANBP2 APBB1IP MZT2A FANK1 UBE2D2 SNAI2 TAOK1 VSIG10 UNC45A ZNF33A SMG9 ABHD6 FLNA FAM155B UTP11L TIFA ZNF658 RPL37 G6PC3 SUCLA2 GALNT16 ABCB8 PRMT7 ZNF562 TTC1 COQ2 MAL2 RNF6 SMYD1 BEX5 PEPD WDR66 VDAC2 TMA7 LANCL1 PCNT DNAJC13 PTCH2 SMIM18 TTC30A OSBPL5 DNAJC19 GPLD1 DNAJC10 SLC39A6 RPS27 C12orf54 |
| **Pink** | 241 | CLK4 HOMEZ EP300 TOB2 DMC1 LUZP1 FBXL14 HCRTR2 FAM198A ZNF35 TLCD2 RP11-548K23.11 AGT ENHO UNG XPC SLC9A3R1 AHNAK DCX NDRG2 HERC6 CD8A CASC10 NRAP KIAA0368 VEGFA CRTAP ZNF165 ASB4 TMBIM1 AXL F13A1 MT1E IRAK2 OLFM4 SPRY1 MSRB2 CAPN2 ITPKB TMEM50B SLC30A1 NTSR2 HTRA1 HBB CCZ1 SLC41A1 SULF1 QRFPR RP11-371E8.4 PPP1R21 FAM46C NDP NEMP1 CHST3 MAEA HOPX ATOX1 EIF4EBP2 POLG2 MSN LRTOMT RGS3 ABHD4 DGKH DDX27 RAPH1 PLEKHB1 RASAL1 NEFM SMC1A ZNF543 PCLO VANGL2 PLS1 VIM FAM167A CAPG ATP1B2 KCNK1 CDH6 S100A1 MR1 ACTN2 CTGF TMEM100 LYPD1 UHRF1BP1 PRDM8 CD59 SERPINE2 TPP1 NQO1 SP1 CLU PCDHB10 KCTD6 C15orf57 C16orf89 STRBP ZMYND12 GBP3 ROM1 RNF212 FAM107A GPRC5B CTSH ZBTB5 IGFBPL1 MFSD9 B3GALT1 PPP1R3G SELENBP1 NPFFR1 ELMOD1 ATRN MLC1 NWD1 ENDOU DAD1 GPR37L1 RHOC NPC2 ALDH2 PLIN2 ADI1 QPRT ERLIN2 CST3 CHI3L1 AHCYL1 ZNF275 SLC25A18 CSRP1 SVEP1 PLIN3 MAP1A ZFP90 NPY1R CHDH BMS1 LRIG1 ABRACL B4GALT4 HIST2H2AC SLC2A1 ANGPTL1 ARHGAP20 PTTG1IP GPC4 WDR61 CGNL1 FEM1C IL17RD TBCE HNRNPA2B1 EIF3A NKAIN4 BTN2A2 MYLK ACSBG1 CD163L1 NOX5 F2R XPNPEP3 CHST7 SLC25A20 ARSD DDX10 SLITRK2 PHTF1 STK19 SMG7 PLPP3 VASP UNC80 RHPN2 FXYD1 RAB9A IL18R1 HSD17B6 TMEM176B ZNF440 S1PR3 KHNYN HPN ARHGAP11A MID1IP1 SYNPO2 FGF11 GPX7 MRGBP BDNF SEMA3E TMEM233 COPZ2 CCNG2 JAG1 ASB3 TTBK2 FGFR2 GM2A SRGAP2 SPEN DHTKD1 UBAP1 DHRS3 DDX4 NOMO3 ECI2 FGFRL1 PGM2 RXRG ASPM PPARA KCNJ1 ATP1A2 ELAVL4 FLT3 SLC1A4 TRABD2A RAB29 LAMTOR1 EPHX1 SLC25A33 NBPF26 PRLR ITM2A C1orf35 XXbac-BPG246D15.9 SLC26A5 PEX11A COL10A1 TMEM254 GPT2 KLF10 UAP1 KDM3A HEATR3 ATP6V0E1 COL6A6 S100A13 |

**S-Table 5.**


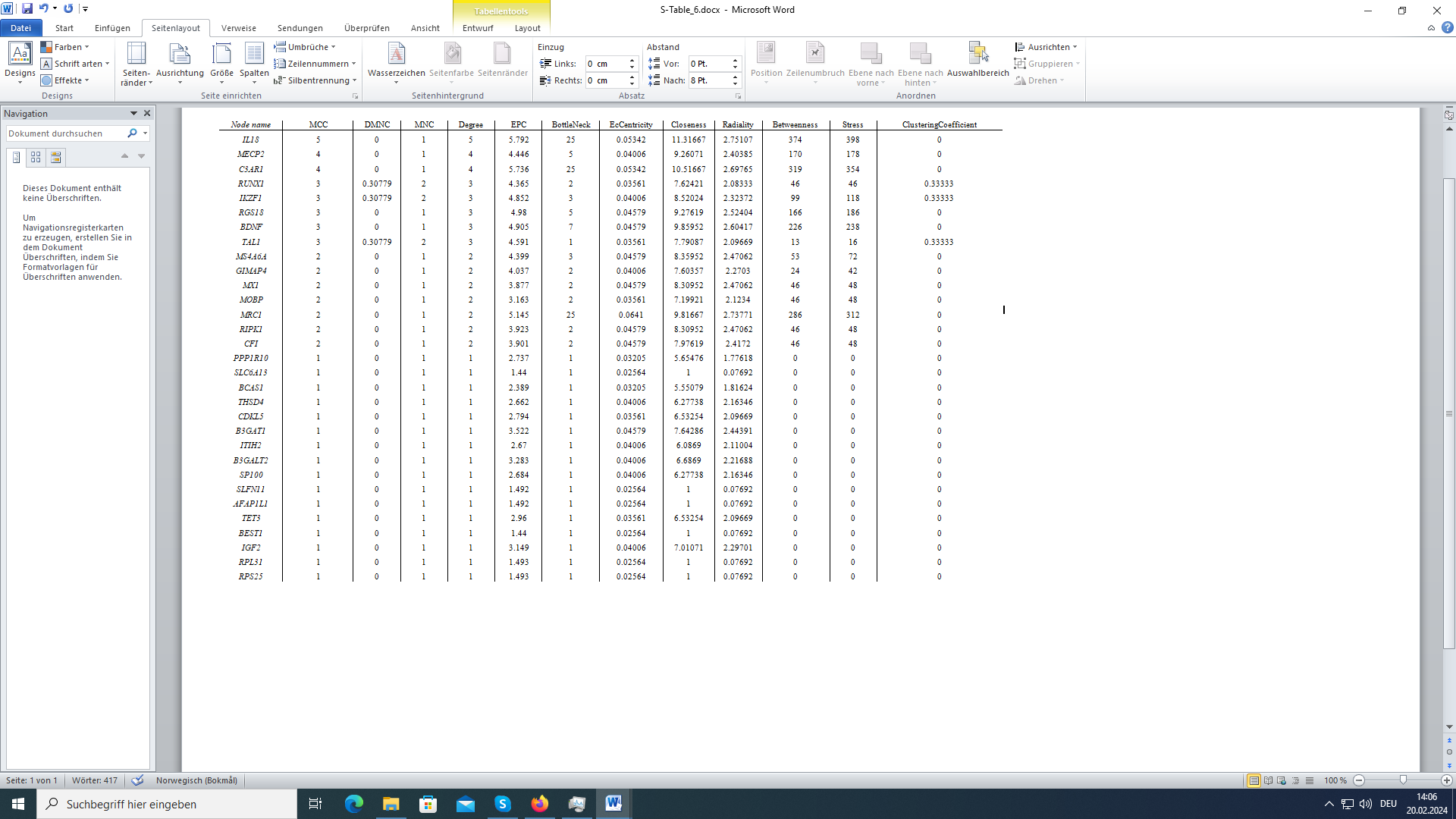


**S-Figure 1.**


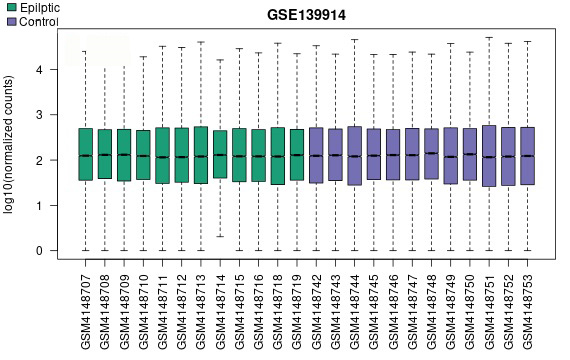


**S-Figure 2.**


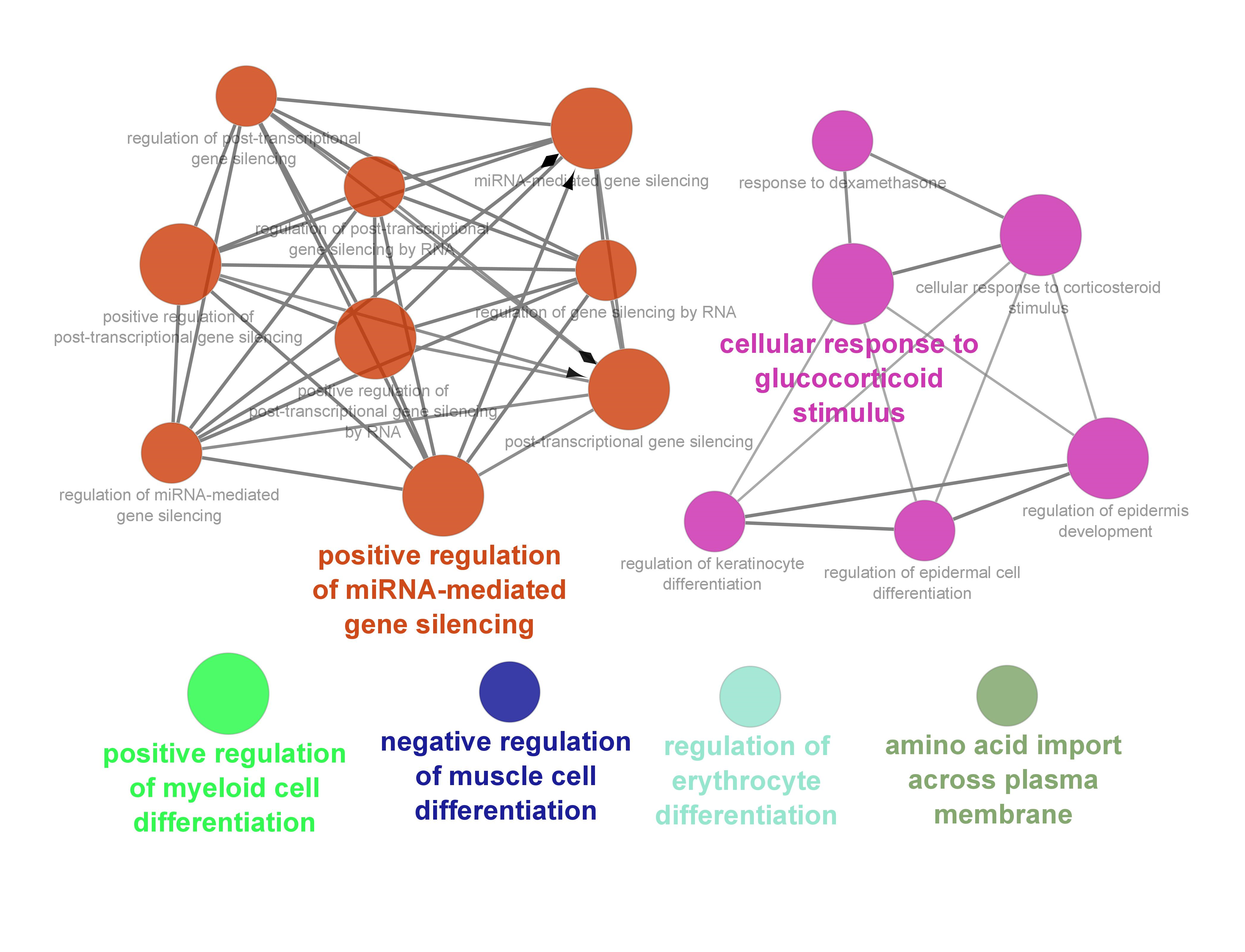


**S-Figure 3.**


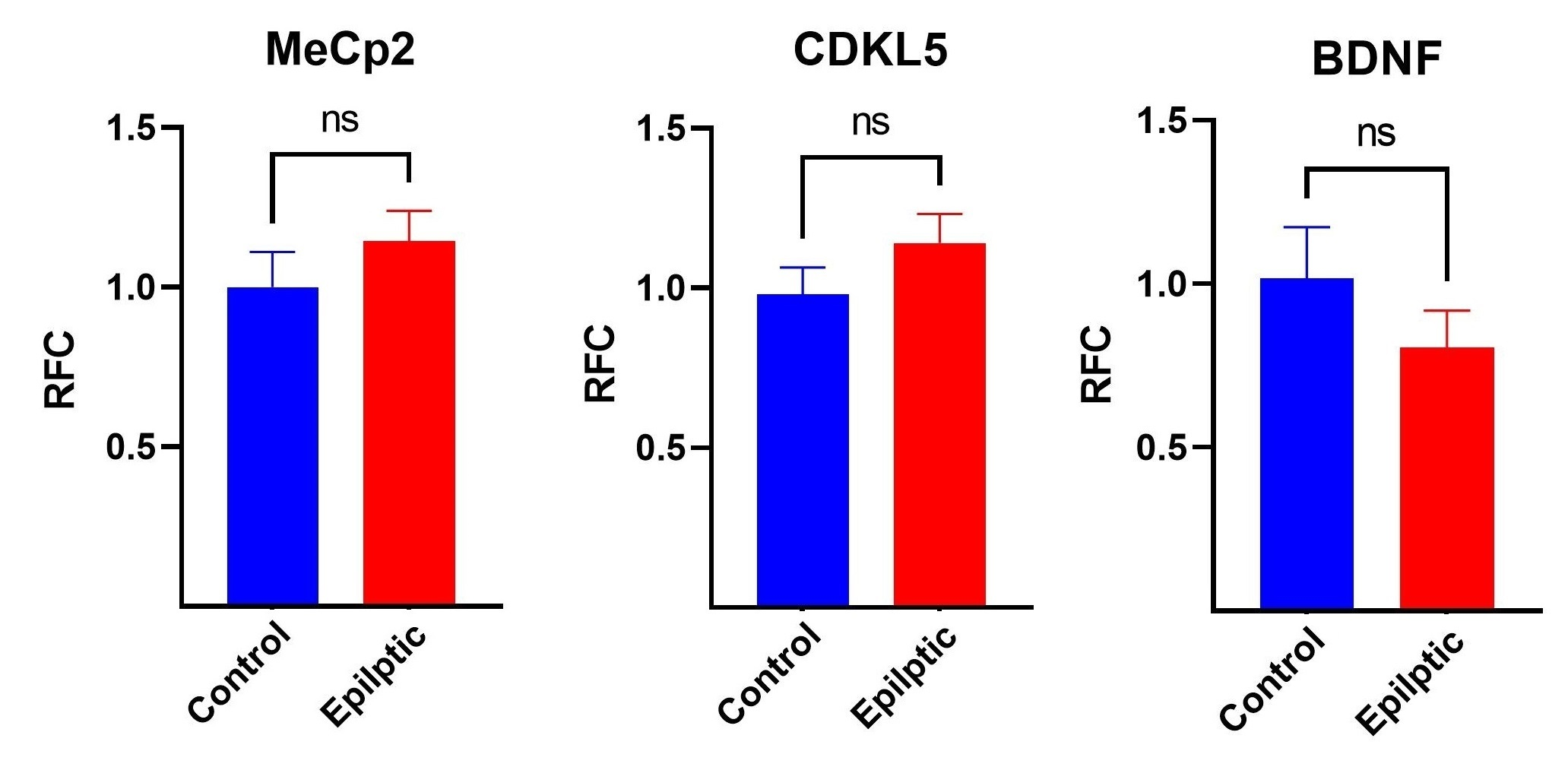

Supplement: Supplementary file 1 — Data S1. Table S1. Downregulated genes in epileptic temporal lobe tissue compared to autopsied tissue. Table S2. Upregulated genes in epileptic temporal lobe tissue compared to autopsied tissue. Table S3. Number of genes belonging to each module. Table S4. Genes coexisting in the turquoise module (MeCp2 co‐expression genes), brown module (CDKL5 co‐expression genes) and pink module (BDNF co‐expression genes) groups, along with differentially expressed genes (DEGs). Table S5. The data of the topological analysis using CytoHubba on the protein–protein interaction network. Figure S1. Box plots illustrating gene expression data. The horizontal axis denotes the sample, while the vertical axis indicates the gene expression values. Figure S2. Gene ontology (GO) biological process enrichment analysis of co‐expression genes and differentially expressed genes (DEGs) involving MeCp2, CDKL5 and BDNF. The network shows groups of terms or pathways associated with these genes. Figure S3. Expression values of the MeCp2, CDKL5 and BDNF genes in epileptic and autoptic control hippocampus. Bar diagrams depicting the relative fold change (RFC) in the relative expression level of the MeCp2, CDKL5 and BDNF genes at mRNA levels. Bars represents the mean ± SEM. ns indicate not significant (n = 20–25). [file JCMM-29-e70373-s001.docx]
